# Dairy intake and cognitive function in older adults in three cohorts: a mendelian randomization study

**DOI:** 10.1186/s12937-025-01083-y

**Published:** 2025-01-31

**Authors:** Natalia Ortega, Nick J. Mueller, Abbas Dehghan, Tosca O. E. de Crom, Armin von Gunten, Martin Preisig, Pedro Marques-Vidal, Marco Vinceti, Trudy Voortman, Nicolas Rodondi, Patricia O. Chocano-Bedoya

**Affiliations:** 1 Institute for Primary Health Care (BIHAM), University of Bern, Bern, Switzerland; 2 Population Health Laboratory (#PopHealthLab), University of Fribourg, Fribourg, Switzerland; 3 Graduate School for Health Sciences, University of Bern, Bern, Switzerland; 4 Department of Epidemiology and Biostatistics, School of Public Health, Imperial College London, London, UK; 5 Dementia Research Institute, Imperial College London, London, UK; 6 MRC Centre for Environment and Health, School of Public Health, Imperial College London, London, UK; 7 Department of Epidemiology, Erasmus MC University Medical Center Rotterdam, Rotterdam, The Netherlands; 8 Center for Primary Care and Public Health, University Center of General Medicine and Public Health, Lausanne, Vaud, Switzerland; 9 Service of Old-Age Psychiatry, Department of Psychiatry, Lausanne University Hospital, Lausanne, Switzerland; 10 Department of Psychiatry, Lausanne University Hospital and University of Lausanne, Lausanne, Switzerland; 11 Department of Medicine, Internal Medicine, Lausanne University Hospital, Lausanne, Switzerland; 12 Department of Biomedical, Metabolic and Neural Sciences, University of Modena and Reggio Emilia, Modena, Italy; 13 Department of Epidemiology, Boston University School of Public Health, Boston, MA USA; 14 Meta-Research Innovation Center at Stanford (METRICS), Stanford University, Stanford, CA USA; 15 Department of General Internal Medicine, Inselspital University Hospital Bern, Bern, Switzerland; 16 Mittelstrasse 43, Bern, 3012 Switzerland

**Keywords:** Lactase persistence, Dairy, Cognitive function, Mendelian randomization, CLSA

## Abstract

**Background:**

Meta-analyses of observational studies on the effect of dairy on cognitive function have yielded inconclusive results, potentially due to unmeasured confounding. To avoid the no-unmeasured confounding assumption, we used lactase persistence genetic variant as an instrumental variable, for which the CC genotype is associated with lower lactase production and, consequently, lower dairy consumption. We used it to assess the effect of long-term consumption of total and non-fermented dairy on cognitive function.

**Methods:**

We included 43,836 individuals over 55 years old with genotyping, dietary data, and cognitive function measurements from three population-based studies: CoLaus|PsyCoLaus (Switzerland), the Rotterdam Study (the Netherlands) and the Canadian Longitudinal Study on Aging (CLSA - Canada). We performed a one-sample Mendelian randomization using two-stage least-squares regression. First, we estimated total and non-fermented dairy consumption by T-allele frequency. Second, we used the estimated dairy consumption in linear regression models on general cognition, assessed by the Mini-Mental State Examination (MMSE) and the Mental Alternation Test, executive function, verbal fluency, verbal learning, and memory.

**Results:**

Per T-allele, total dairy intake and non-fermented was 24.8 and 15.3 g/day higher in PsyCoLaus, 57.9 and 49.8 g/day in the Rotterdam Study, and 0.31 and 0.29 times/day in CLSA, respectively. We found no association between the genetically predicted difference and the MMSE in PsyCoLaus and the Rotterdam Study. However, lactase persistent individuals scored 3.4 (95% CI 2.1− 4.7) and 3.5 (95% CI 2.3–4.7) points more in the Mental Alternation Test for total and fermented dairy, respectively, in CLSA. Similarly, lactase persistent participants in CLSA had higher verbal fluency, verbal learning and executive function, but no differences were found in the other cohorts. Such inconsistencies might stem from different FFQs across cohorts and consumption ranges. Nonetheless, the generally small magnitude of effect sizes may suggest that there is no real effect between total or non-fermented dairy intake and cognitive function.

**Conclusion:**

The evidence for a causal effect of dairy consumption on general cognitive function is weak, consistent with previous results from classic analysis from observational studies. Interventions targeting dairy are unlikely to have a relevant effect on cognitive function.

**Supplementary Information:**

The online version contains supplementary material available at 10.1186/s12937-025-01083-y.

## Introduction

The increasing number of people living with dementia, combined with the absence of medications that can reverse its progression [1, 2], set prevention strategies in the center. Addressing modifiable factors that could increase the risk of cognitive decline, such as obesity or diabetes, could be key for reducing the prevalence of cognitive impairment [3]. However, it is unclear whether targeting nutritional aspects at the population level could be beneficial.

Dairy consumption is recommended in many countries [4] due to its rich composition of micro- and macronutrients. Several studies have explored the relationship between dairy consumption and the risk of cognitive decline and dementia, hypothesizing that components like short and medium chain fatty acids, vitamins A, B complex and C, calcium and magnesium could contribute to reduce the cardiovascular risk regulating blood pressure and decreasing the risk of vascular dementia, and decrease inflammation which has been linked to lower cognitive function [5]. Among the three available meta-analyses, one found that higher average milk consumption was associated with lower risk of Alzheimer’s disease and cognitive disorders [6]. The other two concluded that the existing evidence does not support that dairy intake could prevent cognitive decline [7, 8]. The observational studies included in these meta-analyses, in the absence of experimental studies, were heterogeneous in their dietary and cognitive assessment methods, at high risk of residual confounding, selection and measurement bias, and reverse causation in cross-sectional studies. Additionally, the generalizability remains a concern as different regions of the world have different levels of dairy intake [6–8].

Triangulating the evidence from observational studies on the relationship between total and non-fermented dairy, mainly milk, and cognitive functioning in different populations using LCT-13,910 C/T genetic variant (rs4988235) as an instrumental variable is an alternative approach subject to different identification assumptions. LCT-13,910 C/T is the mutation that determines the ability of producing lactase, the enzyme digesting lactose in adults with European ancestry. Inheriting the CC genotype (C_− 13910_ homozygotes) leads to lower lactase enzyme activity (lactase non-persistence). In Europe, CC genotypes range from 10% prevalence in Nordic countries, to 70% in Italy or Turkey [9]. In the US and Canada, around 50% of the population carries the CC genotype [10]. Previous studies confirmed higher milk consumption among lactase persistent individuals (TC and TT genotypes) compared to lactase non-persistent individuals (CC genotype) [11, 12]. The use of randomly inherited genetic variants (also known as Mendelian randomization studies) as instrumental variables for specific exposures, when the identifiability assumptions hold, yield causal effect estimates [13]. Three Mendelian randomization studies investigated potential neurological effects of milk consumption and concluded that higher intake was associated with a lower risk for Alzheimer’s disease and Multiple Sclerosis, and a higher risk of Parkinson’s disease [14–16], but none investigated the effect of non-fermented dairy, on cognitive function measures.

Here, we hypothesized that the effect found between dairy and cognitive function in observational studies may be due to residual confounding. Therefore, we aimed to determine the long-term effect of non-fermented dairy products intake on cognitive function among older adults, using lactase persistence as an instrument to triangulate the results previously reported in meta-analyses of observational studies.

## Methods

### Study design and population

Our target population was healthy adults aged 55 years and older. To represent this population, we selected three population-based cohorts from different geographic regions – CoLaus|PsyCoLaus (PsyCoLaus) in Switzerland [17], the Rotterdam Study (RS) in the Netherlands [18], and the Canadian Longitudinal Study on Aging (CLSA) in Canada [19] – over 55, who were cognitively healthy, who had information on socioeconomic and clinical characteristics and dairy intake, who underwent a cognitive function assessment, and provided blood samples that were genotyped. A summary of the recruitment strategies, participation rates, inclusion dates and follow-ups is provided in Supplementary Table 1.

Participants from the three cohorts signed an informed consent and a further use of the data form to allow the use of the data for other purposes other than those initially proposed. The local ethics committees issued ethics approvals for each study and the Cantonal Ethics Committee for research in Bern under the number 2022 − 01976.

### Measurement of non-fermented dairy intake

Total and non-fermented dairy intakes were assessed with food frequency questionnaires (FFQs) in the three cohorts. PsyCoLaus and RS used a semi-quantitative 97-item [20, 21], and 389-item [22, 23], respetively, and CLSA a 36-item non-quantitative FFQ [24]. For each food item, participants in PsyCoLaus and RS self-reported the average frequency of consumption based on a common unit or portion size (e.g., 200 mL/d of milk in PsyCoLaus). In RS, participants reported their average intake in the previous year, while in PsyCoLaus, they reported their average intake over the past four weeks. CLSA reported number of times per day consumption without further specification of the number of portions, servings or total amount per time. Total dairy included all dairy products reported. Non-fermented dairy products included milk (skimmed, semi-skimmed, full fat), cream and butter which were modeled as a continuous variable in grams per day (g/d) in PsyCoLaus and RS, and in times/d in CLSA.

### Genetic instrument

We defined as non-lactase persistent participants with the CC genotype and as lactase persistent the CT and TT genotypes. We considered a p-value of the Chi-square test for the Hardy-Weinberg equilibrium (HWE) to be significant < 0.05 given the medium sample sizes from the individual studies. In PsyCoLaus, nuclear DNA was extracted from the whole blood for whole genome scan analysis. The genotyping was performed using Affymetrix Axiom Array 500 K SNP chip. The LCT-13,910 frequencies did not follow a HWE in PsyCoLaus (p-value = 2.8 × 10^− 6^). Allele A frequency was 0.85. In RS, the genetic data were imputed using MACH with HapMap Release 22 CEU build 36 as a reference panel with an imputation quality score of 92% [25]. Allele A frequency was 0.69. The genotype distribution of the LCT-13,910 C polymorphism did not follow HWE because fewer heterozygotes were present than expected (p-value = 0.0001). The RS also tested the genomic inflation factor (1.013) for body height which was unlikely to reflect important population stratification [26]. In CLSA, genotyping was performed using the Affymetrix Axiom Array 794 K SNP chip. Imputation was conducted using the TOPMed reference panel at the University of Michigan Imputation Service reporting an Imputation Quality Score over 82% (https://www.clsa-elcv.ca/doc/2748) [27]. The ApoE variant (rs429358) was used in sensitivity analyses and did follow the HWE in PsyCoLaus (r^2^ 0.75, HWE p-value = 0.19) and CLSA (r^2^ NA, HWE p-value > 0.05).

Lactase persistence variant (rs4988235) can be considered an instrument if (1) it is causally associated with total and non-fermented dairy consumption (relevance), (2) it is independent from the confounders affecting non-fermented dairy consumption and cognitive decline (marginal exchangeability) and (3) it is potentially causing cognitive decline only through non-fermented dairy consumption (exclusion-restriction) [28]. To test relevance (assumption 1), we regressed lactase persistence and total and non-fermented dairy consumption and used the F-statistic (> 10), Wu-Hausman and r^2^ [29]. Fermented dairy did not fulfill the relevance assumption and therefore we did not conduct the analyses (Supplementary File 4). We also ruled out that it was a rare variant (minor allele frequency < 5%) as rare variants are more prone to false-positive results. Moreover, we chose this variant because its causal effect on milk consumption has been well described [11, 12]. For exchangeability (assumption 2), we heavily rely on the random inheritance of the genetic variants. However, we adjusted our two-stage models for sex, age, body mass index and height (as a proxy for population stratification) to avoid any shared common causes between the lactase persistence and non-fermented dairy intake. Adjustment for height and BMI can account for violations of the independence assumption as it has been shown in previous studies that its relationship with health outcomes is due to dynastic effects, assortative mating and population stratification [30]. Lastly, we were unable to directly test the exclusion restriction (assumption 3), although there is no existing evidence to suggest that the genetic variant used has a direct effect on cognition.

Besides the three assumptions on valid instruments, instrumental variable estimands also rely on a fourth assumption based on the hypothesized exposure-outcome relationship. Under linearity and homogeneity assumptions, we could compute the Average Causal Effect. However, this assumes that the effect of non-fermented dairy on cognition is constant across individuals within the levels of baseline covariates. Because this is a strong and implausible assumption to make, we computed the Complier Average Causal Effect (CACE) that is the causal effect for participants who would consume non-fermented dairy if lactase persistent and would not if lactase non-persistent, and those who would never consume non-fermented dairy if lactase non-persistent or lactase persistent or would always eat non-fermented dairy if lactase non-persistent or lactase persistent. We assumed that there were no defiers (participants who would consume non-fermented dairy if lactase non-persistent and would not if lactase persistent). We think that this assumption, known as monotonicity, is more plausible [31].

### Cognitive function assessment

Our primary outcome was general cognition, assessed with the MMSE in PsyCoLaus and RS and with the Mental Alternation Test (MAT) in CLSA [32]. The MMSE involves spoken replies to evaluate orientation, memory and attention, the capacity to name objects, follow written or verbal instructions, spontaneously write a sentence or recreate a geometric Fig. (33). We considered a 1-point difference in the MMSE as the lower bound for a minimally clinically relevant effect for this measure [34, 35]. The MAT consists of an alternating series of numbers and letters and demands timed performance and category-switching assessing executive function. It is used as a screening tool to detect cognitive impairment [36], and therefore we defined it as representing general cognition, for which we could not find studies defining clinically relevant changes in the score.

Our secondary outcomes assessed four domains of cognition: processing speed and executive function, verbal learning, episodic memory, and verbal fluency. Executive function and processing speed were assessed with the Stroop test [37–39]. The Stroop Color test sets a clinically relevant difference of 5.5 points (seconds) among a healthy population at baseline [35]. CLSA and RS measured verbal learning with the Rey Auditory Verbal Learning Test [40]. Episodic memory was assessed with the Free and Cued Selective Reminding Test [41] in PsyCoLaus and the Prospective Memory Task [42] in CLSA. No studies have assessed clinically significant differences for either memory tests or verbal learning. Verbal fluency was assessed with the animal naming test [43] in all three cohorts and we considered a minimally relevant clinical difference at 2.9 points [35]. All of them were coded as continuous outcomes. We summarized tests’ features in Supplementary Table 2.

### Statistical analysis

We conducted a one sample Mendelian randomization because genetic variants, exposures, and outcomes were measured in the same individuals. The one-sample setting allows for comparison with covariate-adjusted studies and for unbalanced covariate adjustment. The analytic population consisted of participants who fulfilled the selection criteria: over 55 years old and having information on the genetic variant of interest, dietary assessment, and healthy baseline cognition (not diagnosed with dementia and a MMSE > 26).

We performed three separate analyses in the three cohorts using two-stage least-squares regression. In the first stage, the continuous exposure (total and non-fermented dairy intake) was regressed on the continuous probabilities of the genetic variant and the relevant covariates (age at baseline – continuous), sex, height (continuous) and body mass index (BMI) (continuous). We included height to prevent population stratification and BMI to potentially avoid dynastic effects [30]. In the second stage, cognitive function test scores were then regressed on the predicted values of the exposure from the first regression and the same covariates. We computed robust confidence intervals.

We performed a series of sensitivity analyses to check how robust are the results to our modelling assumptions. First, we compared two-stage least-squares method with the ratio method [44]. In a one-sample setting with individual-level data, a causal effect estimate can also be obtained using the ratio or Wald method [45]. Second, we considered the ApoE variant (rs429358) as a positive control exposure - an invalid instrument for non-fermented dairy and cognition because it is known to have a causal effect on cognition but not on non-fermented dairy intake. If it was found to be associated with non-fermented dairy intake but not associated with lower cognitive function, it would indicate violation of instrumental variable assumptions through pleiotropy or population stratification. Third, we excluded butter from the non-fermented dairy products as they make the group more heterogeneous. Fourth, we excluded BMI as a confounder, as it may act as a mediator in the pathway between dairy consumption and cognitive function, potentially distorting the targeted total causal effect. Fifth, we adjusted for the first four principal components in PsyCoLaus to check if our results are robust for pleiotropy and population stratification.

This manuscript was developed according to version 3 of the Guidelines for performing MR investigations [46], STROBE-nut and STROBE-MR reporting guidelines [47]. The analyses were performed using R version 4.3.3 and the package ivreg [48]. We provide these checklists in Supplementary File 1 and sample code and pooled results in Supplementary File 2.

## Results

We included 43,836 participants in our analysis with an average follow-up of 5.2 years. Individual characteristics from the three cohorts stratified by lactase persistence status are presented in Table 1. The proportion of female participants ranged from 51.2% in CLSA to 57.9% in RS. The mean age was 68.5, 65.9 and 62.9 years, in PsyCoLaus, RS and CLSA, respectively. Compared to the other cohorts, participants in PsyCoLaus achieved the lowest education, had the highest proportion of cardiovascular events (20.4%), and the lowest proportion of obesity (20.1%). RS had the highest proportion of current smokers (17.9%) and overweight individuals (46.5%). In CLSA, there were more men than in the other cohorts, highly educated (53%) and never smokers (47.4%) (Supplementary Table 3). 21% of participants in PsyCoLaus were lactase non-persistent, 8% in RS and 18% in CLSA. Unlike all other characteristics, BMI was unbalanced in PsyCoLaus and RS. The proportion of non-lactase persistent participants with obesity was higher than in lactase persistent participants, and the proportion of lactase persistent participants with a normal weight was higher than among non-lactase persistent.

**Table 1:** Baseline characteristics of the study population by cohort and exposure status

	PsyCoLaus			Rotterdam Study			CLSA		
	NLP (*n* = 327)	LP (*n* = 1238)	SMD	NLP (*n* = 694)	LP (*n* = 7688)	SMD	NLP (*n* = 4520)	LP (*n* = 21186)	SMD
Sex (Male) (%)	157 (48.0)	507 (41.0)	0.142	284 (40.9)	3243 (42.2)	0.026	2339 (51.7)	10,475 (49.4)	0.046
Age (%)			0.041			0.139			0.041
Below 70	201 (61.5)	747 (60.3)		183 (55.3)	2293 (59.8)		3365 (74.4)	15,386 (72.6)	
Between 70 and < 75	70 (21.4)	286 (23.1)		107 (32.3)	1001 (26.1)		427 (9.4)	2136 (10.1)	
75 or above	56 (17.1)	205 (16.6)		41 (12.4)	543 (14.2)		728 (16.1)	3664 (17.3)	
Education (%)			0.144			0.025			0.045
Basic	210 (64.2)	871 (70.4)		388 (56.1)	4353 (56.9)		312 (8.1)	1574 (8.8)	
Secondary	56 (17.1)	195 (15.8)		201 (29.0)	2135 (27.9)		1415 (36.8)	6901 (38.4)	
Higher	61 (18.7)	172 (13.9)		103 (14.9)	1158 (15.1)		2116 (55.1)	9496 (52.8)	
BMI categories (%)			0.111			0.111			0.061
Normal (BMI < 25)	115 (35.8)	479 (39.1)		118 (24.8)	1565 (29.8)		1407 (31.3)	6301 (29.8)	
Overweight (BMI 25-29.9)	59 (18.4)	252 (20.6)		234 (49.3)	2435 (46.3)		1886 (41.9)	8577 (40.6)	
Obese (BMI > 30)	147 (45.8)	494 (40.3)		123 (25.9)	1260 (24.0)		1206 (26.8)	6241 (29.6)	
Smoking (%)			0.064			0.042			0.052
Current	48 (14.7)	181 (14.7)		74 (18.6)	768 (17.3)		398 (8.8)	1882 (8.9)	
Former	135 (41.4)	548 (44.4)		200 (50.4)	2228 (50.2)		1882 (41.6)	9341 (44.1)	
Never	143 (43.9)	505 (40.9)		123 (31.0)	1446 (32.6)		2240 (49.6)	9963 (47.0)	
CV event* (%)	76 (23.2)	242 (19.6)	0.089	12 (1.7)	156 (2.0)	0.022	523 (11.6)	2561 (12.1)	0.016
Hypertension (%)	223 (68.2)	833 (67.4)	0.017	267 (70.4)	3032 (70.4)	0.001	1639 (36.3)	7803 (36.9)	0.011
Diabetes (%)	42 (12.8)	144 (11.6)	0.037	96 (17.2)	1192 (19.5)	0.061	833 (18.5)	3653 (17.3)	0.032
Physical Activity (%)			0.055			0.083			0.007
Low	130 (45.3)	493 (45.7)		123 (37.4)	1270 (33.7)		1450 (33.6)	6762 (33.2)	
Medium	96 (33.4)	379 (35.1)		106 (32.2)	1239 (32.9)		1420 (32.9)	6734 (33.1)	
High	61 (21.3)	207 (19.2)		100 (30.4)	1261 (33.4)		1449 (33.5)	6843 (33.6)	
Baseline MMSE/MAT (mean (SD))	29.3 (1.2)	29.4 (1.1)	0.039	27.7 (2.4)	27.8 (2.2)	0.047	25.7 (8.8)	26.9 (8.7)	0.129

NLP: Non-lactase persistent (CC); LP: Lactase persistent (CT/TT); CLSA: Canadian Longitudinal Study on Aging; CV: Cardiovascular; MMSE: Mini-Mental State Examination; MAT: Mental Alternation Test; SMD: Standardized mean difference. *In PsyCoLaus and CLSA includes self-reported cardiomyopathy, congenital heart disease, valvular heart disease, heart failure, coronary artery disease, angina, myocardial infarction, stroke, percutaneous coronary intervention, coronary artery bypass graft or pacing. In Rotterdam Study includes stroke events

Non-lactase persistent participants (CC-genotype) had lower average total and non-fermented dairy consumption compared to lactase persistent individuals in all three cohorts (Table 2). Total dairy intake was 24.8 g/d (95% CI 1.8–47.7) higher in PsyCoLaus, 57.9 g/d (95% CI 29.6–86.3) higher in RS, and 0.31 times/d (95% CI 0.26–0.36) higher in CLSA. Non-fermented dairy intake was 15.3 g/d (95% CI 1.8–83.5) higher in PsyCoLaus, 49.8 g/d (95% CI 22.9–69.5) higher in RS and 0.29 times/d (95% CI 0.25–0.32) higher in CLSA. We observed high instrument strength in CLSA and moderate strength in PsyCoLaus and RS for non-fermented dairy, and high instrument strength in CLSA, moderate strength in RS and low strength in PsyCoLaus for total dairy (Supplementary Table 4).

**Table 2:** Mean non-fermented dairy intake (grams/day) and mean differences per cohort by lactase persistence genotype

	Dairy type	NLP (SE)	LP (SE)	Difference (95% CI)
CoLaus|PsyCoLaus	Total	229.7 (10.4)	254.5 (11.7)	24.8 (1.8–47.7)
	Non-fermented	71.5 (6.1)	86.8 (3.1)	15.3 (1.8–83.5)
Rotterdam Study	Total	280.2 (13.9)	338.1 (14.5)	57.9 (29.6–86.3)
	Non-fermented	147.2 (11.8)	197.0 (3.5)	49.8 (22.9–69.5)
CLSA*	Total	2.6 (0.02)	2.9 (0.02)	0.31 (0.26–0.36)**
	Non-fermented	1.4 (0.02)	1.7 (0.01)	0.29 (0.25–0.32)**

*times/d, once/d corresponding to 250 g/d of milk ** Approximately corresponding to 73 g/d - CLSA performed s qualitative FFQ. NLP: Non-lactase persistent (CC genotype); LP: Lactase persistent (CT/TT genotypes). CLSA: Canadian Longitudinal Study on Aging, SE: Standard Error.

The mean difference in MMSE per 50 g/d higher total and non-fermented dairy consumption was 0.18 (95% CI −0.65 to 1.0) and 0.17 (95% CI −0.60 to 0.95) in PsyCoLaus, and − 0.20 (95% CI −0.56 to 0.17) and − 0.28 (95% CI −0.83 to 0.26) in RS, respectively. The MAT was 3.38 (95% CI 2.11 to 4.65) higher for total and 3.50 (95% CI 2.26 to 4.73) higher for non-fermented dairy among lactase persistent individuals in CLSA. The domain-specific cognitive function measures did not differ by T-allele in PsyCoLaus and RS. Nonetheless, we found 3.1 points (95% CI 2.1 to 4.0) higher executive function in lactase persistent individuals, 2.0 points (95% CI 1.3 to 2.8) higher verbal fluency, higher verbal learning 0.9 points (95% CI 0.5 to 1.2) and 0.7 points (95% CI 0.5 to 0.9) higher memory in CLSA (Table 3), for both total and non-fermented dairy. Crude coefficients are presented in Supplementary Table 5.

**Table 3:** Mean differences in scores (95% CI) for global cognitive function and four cognitive domains per 50 g/day intake difference in the three cohorts

	Dairy type	CoLaus|PsyCoLaus (*n* = 1565)	Rotterdam Study (*n* = 8382)	CLSA (*n* = 29046)
MMSE/MAT	Total	0.18 (-0.65 to 1.00)	-0.20 (-0.56 to 0.17)	3.38 (2.11 to 4.65)
(range: 0–30/0–52)	Non-fermented	0.17 (-0.60 to 0.95)	-0.28 (-0.83 to 0.26)	3.50 (2.26 to 4.73)
Executive function	Total	0.88 (-2.10 to 3.86)	3.80 (-1.68 to 9.28)	3.05 (2.09 to 4.00)
(difference in seconds)	Non-fermented	0.49 (-0.42 to 1.40)	5.66 (-3.69 to 15.00)	3.08 (2.19 to 3.96)
Verbal fluency	Total	1.65 (-6.07 to 9.37)	0.77 (-0.67 to 2.21)	2.03 (1.30 to 2.76)
(range: 0–58 words)	Non-fermented	0.95 (-2.66 to 4.55)	1.02 (-0.97 to 3.01)	2.17 (1.43 to 2.92)
Verbal learning	Total	-	0.21 (-0.28 to 0.70)	0.87 (0.53 to 1.21)
(range: 0–8 in RS 0–15 in CLSA)	Non-fermented	-	0.26 (-0.35 to 0.87)	0.89 (0.56 to 1.22)
Memory	Total	-1.60 (-7.40 to 4.18)	-	0.67 (0.47 to 0.88)
(range: 0–48 in PsyCoLaus and 0–9 in CLSA)	Non-fermented	-0.97 (-3.59 to 1.66)	-	0.71 (0.51 to 0.90)

CLSA: Canadian Longitudinal Study on Aging; MMSE: Mini-Mental State Examination; MAT: Mental Alternation Test; FCSRT: Free and Cued Selective Reminding Test; PMT: Prospective Memory Test

The results from the sensitivity analyses largely confirmed our findings. First, our analysis yielded similar results when using the ratio method compared to two-stage least-squares regression (Supplementary Table 6). The suitability of two-stage least-squares regression was confirmed with the Wu-Hausman statistic in PsyCoLaus and CLSA (Supplementary Table 4). Second, our positive control, ApoE, was not related to non-fermented dairy intake (Supplementary Table 7 A) and was related to lower cognitive function in CLSA but not in PsyCoLaus, potentially because of suboptimal imputation quality of 75% (Supplementary Table 7B). Third, removing BMI from the models (Supplementary Table 8), removing butter from the exposure variable (Supplementary Table 9), and adjustment for the four first principal components as an alternative way to deal with population stratification (Supplementary Table 10) resulted in similar estimates.

## Discussion

Neither total dairy nor non-fermented consumption was not associated with overall or domain-specific differences in cognitive function in the Dutch and Swiss populations, as estimated using lactase persistence as an instrument. However, we found higher scores for general cognition and all domain-specific cognitive measures in lactase persistent compared to non-lactase persistent individuals in the Canadian population.

The differences in general cognition were of small magnitude and clinically irrelevant, defined as a difference smaller than 1-point in the MMSE score [34, 49, 50], in PsyCoLaus and RS. In CLSA, general cognition, measured with the MAT, was higher among lactase persistent individuals. There is no literature discussing clinically relevant effects for the MAT, so we cannot rule out that our estimates are of clinical relevance.  The bounds of the confidence intervals for executive function, defined as 5.5 points for clinical relevance in the Stroop test [35, 49], did not include clinically relevant estimates in PsyCoLaus and CLSA but they did in RS. The higher verbal fluency among lactase persistent individuals could be of significant clinical relevance only in PsyCoLaus, set at 2.9 points [35, 49], however they were non-informative. Verbal learning and memory lack thresholds for clinical relevance, but they were either close to null or non-informative in the three cohorts. We interpreted CLSA estimates with caution because the cohort used a non-quantitative FFQ, more susceptible to exposure measurement bias than quantitative FFQs. This could impact the first stage regression and lead to an overestimate of the effect. Moreover, the effect is expressed as once/d more of non-fermented dairy consumption (approximately corresponding to 250 g/d if we consider one portion of a standard size), that is of higher magnitude than an increase of 50 g/d in PsyCoLaus and RS. Although the increased power given the bigger sample size can give us more precise estimates, they are mostly of small magnitude below the clinical relevance for executive function and verbal fluency.

Overall, our results suggest there is little evidence for an effect of total and non-fermented dairy on cognitive function. Therefore, a recommendation for increasing total or non-fermented dairy intake might have a low impact, if any, in general cognitive function in Canadian, Dutch and Swiss populations through the wide range of total and non-fermented dairy intakes observed (range: 0–960 g/d and 0–17 times/d). These populations represent healthy European adults with Caucasian ancestry with similar lactase persistence prevalence and cultural norms related to dairy consumption. Consequently, our results might not generalize to populations with non-Caucasian ancestry because they are not included in the panels for genotyping and to individuals falling outside of the observed ranges of dairy consumption. Extending any inferences to these populations would require genotyping panels that include participants from all ethnic backgrounds, where the frequency of the genetic variant is likely lower and therefore the validity of lactase persistence as an instrument would need to be confirmed.

We confirmed the validity of lactase persistence as a valid instrument for non-fermented dairy consumption, as previously suggested by some studies in the Swedish and Danish populations [51, 52], and also for total dairy even if slightly weaker. The two studies that explored the relationship between the same genetic variant and Alzheimer’s disease in two-sample mendelian randomization found similar null and small results. Zhang [14] reported 3% lower odds of Alzheimer’s disease per 50 mL milk/d [odds ratio 0.97 (95% CI 0.95–0.99)] and Yuan [16] reported no association in a phenome-wide study. In the same study, protective associations of milk consumption were found for cataracts, diabetes and some dyslipidemias. Especially cardiometabolic outcomes could increase the risk of cognitive decline [53]. With no overlap between the included cohorts, our results align with the small effect sizes observed indicating an effect close to the null. Comparing these results with the ones from classical observational analyses, similar small effect sizes were found in PsyCoLaus [54], and meta-analyses on the association also found overall no effect of total and milk dairy consumption on either Alzheimer’s disease or specific cognitive function domains [6–8]. Thus, the current study suggests that associations found in classic observational studies may be related to residual confounding, reverse causation, or selection bias.

We included 43,836 individuals in a one-sample Mendelian randomization. Performing a one-sample Mendelian randomization, as opposed to two-sample Mendelian randomization, makes our results comparable to other epidemiological studies using different methods in similar and identifiable populations, and allowing harmonization of the covariates included in the two-stage least-squares regression in the three cohorts. By choosing to adjust for BMI (not possible in a two-sample setting), we were able to mitigate potential dynastic effects, where the genotype of the parents influences the offspring’s exposure not through their genotype, creating a dependency between the randomization of lactase persistence and non-fermented dairy consumption. Height adjustment and restricting our population to Caucasian individuals could have prevented some bias due to population stratification. Lactase persistence is likely to be a valid instrument with biological and causal relationship to non-fermented dairy consumption, making the relevance assumption more plausible. The instrumental variable analysis moved away from the unmeasured confounding assumption and potential reverse causation in observational studies with short follow-up periods to replace it with equally strong but alternative assumptions to triangulate the evidence on the effect of dairy consumption on cognitive function. Additionally, by including populations with different prevalences of lactase persistence and cultural norms regarding non-fermented dairy products, our results cover a wide spectrum of populations that may share these characteristics.

The present study shares some limitations with observational studies. We rely on self-reported dairy intake in FFQs, that in the case of CLSA was non-quantitative and therefore more prone to measurement bias. The measurement bias could affect our analyses in any direction if it is systematic (e.g., older people consume less quantity per time/day) and towards the null if random, which we consider unlikely and thus, hard to predict the direction of the bias. Selection bias may also be a concern because we condition on not being censored due to death, loss to follow-up or missing data, and their determinants may differ between the groups compared (e.g., smokers may be unbalanced over the follow-up because they die before developing dementia, even if they were balanced at baseline). Moreover, the cohorts probably included the healthiest individuals in the population, so our results may not apply to participants with severe cognitive decline and genetic variants may not be distributed randomly in this selected sample [55]. Finally, the clinical significance thresholds were mostly determined in patients with mild cognitive impairment or dementia, which may not equal improvements in healthy individuals at the population level who usually benefit from smaller effect sizes.

Some other limitations are specific to the instrumental variable analysis assumptions. The relevance assumption was confirmed empirically, and we have a moderately strong instrument in PsyCoLaus and RS and a strong instrument in CLSA. Even with a moderately strong instrument, some bias can be introduced in one-sample Mendelian randomization resulting in biased estimates in the direction of the confounded association and higher false positive rates. We do not think this is affecting our results because in the *moderate strength* cohorts we observed no association between total and non-fermented dairy consumption and cognitive function. Moreover, both populations had a high (not rare) prevalence of non-lactase persistent individuals. A downside of one-sample and single instrumental variable analysis is that we cannot assess the plausibility of exclusion restriction in sensitivity analyses. None of our cohorts followed the HWE, meaning that the lactase persistence genetic variant is not constant across generations susceptible to mutations, genetic drift (random change in allele frequency in populations), natural selection (when a variant presents a trait advantage), gene flow (individuals moving to populations with different allele frequencies) and assortative mating (some individuals with the variant are more likely to mate with individuals with a particular genotype compared to random mating). We could not investigate the effect of fermented dairy because lactase persistence is a weak instrument for that aim because of limited lactose levels in this subgroup. It is therefore challenging to compare our results quantitatively to observational studies focusing on fermented dairy products and that might affect cognition differently due to their higher content of short-chain fatty acids, prebiotics, salt and saturated fatty acids. We assumed that the relationship between lactase persistence and non-fermented dairy consumption remains stable over time during late adulthood, which might not be the case given that humans lose the ability to produce lactase if we considered different dairy consumption assessment timepoints. Including one genetic variant reduces power and results could be biased if there were pleiotropic pathways. Pleiotropic effects occur when the gene affects other pathways related with cognition, which we think is unlikely, as none have been described in the literature. The validity of our estimates depends on the assumption of independence of genetic variants from these two potential confounders at the population-family level.

## Conclusion

Lactase persistence is a potentially valid instrument to estimate the effect of total and non-fermented dairy consumption on cognitive function. Our results, inconsistent across cohorts, support that the similar effect estimates reported in classic observational studies on the association between dairy and cognitive function in the general population, are weak and most likely non-causal. Interventions targeting dairy are unlikely to have a relevant effect on cognitive function.

## Electronic supplementary material

Below is the link to the electronic supplementary material.

Supplementary Material 1

Supplementary Material 2

## Data Availability

The data of the CoLaus|PsyCoLaus study used in this article cannot be fully shared as they contain potentially sensitive personal information on participants. According to the Ethics Committee for Research of the Canton of Vaud, sharing these data would be a violation of the Swiss legislation with respect to privacy protection. However, coded individual-level data that do not allow researchers to identify participants are available upon request to researchers who meet the criteria for data sharing of the CoLaus|PsyCoLaus Datacenter (CHUV, Lausanne, Switzerland). Any researcher affiliated to a public or private research institution who complies with the CoLaus|PsyCoLaus standards can submit a research application to research.colaus@chuv.ch or research.psycolaus@chuv.ch. Proposals requiring baseline data only, will be evaluated by the baseline (local) Scientific Committee (SC) of the CoLaus and PsyCoLaus studies. Proposals requiring follow-up data will be evaluated by the follow-up (multicentric) SC of the CoLaus|PsyCoLaus cohort study. Detailed instructions for gaining access to the CoLaus|PsyCoLaus data used in this study are available at www.colaus-psycolaus.ch/professionals/how-to-collaborate/.Data from the Rotterdam Study cannot be made publicly available because of restrictions based on privacy regulations and informed consent of the participants. Proposals for data access can be directed to the management team of the Rotterdam Study (secretariat.epi@erasmusmc.nl), which has a protocol for approving data requests.Data are available from the Canadian Longitudinal Study on Aging (www.clsa-elcv.ca) for researchers who meet the criteria for access to de-identified CLSA data. We provide detailed sample code and pooled results for CoLaus|PsyCoLaus in Supplementary File 2.

## Declarations

### Disclaimer

The opinions expressed in this manuscript are the author’s own and do not reflect the views of the Canadian Longitudinal Study on Aging.

## References

[1] Gauthier SWC, Servaes S, Morais JA, Rosa-Neto P. World Alzheimer Report 2022: life after diagnosis: navigating treatment, care and support. London, England: Alzheimer’s Disease International.; 2022.

[2] Nations U. World Population Ageing. United Nations. Population Division of the United Nations Department of Economic and Social Affairs; 2020.

[3] Livingston G, Huntley J, Sommerlad A, Ames D, Ballard C, Banerjee S, et al. Dementia prevention, intervention, and care: 2020 report of the Lancet Commission. Lancet. 2020;396(10248):413–46. 32738937 10.1016/S0140-6736(20)30367-6 PMC7392084

[4] Weaver CM. How sound is the science behind the dietary recommendations for dairy? Am J Clin Nutr. 2014;99(5):S1217–22. 10.3945/ajcn.113.073007 PMC6410894 24646824

[5] Gaucheron F. Milk and dairy products: a Unique Micronutrient Combination. J Am Coll Nutr. 2011;30(sup5):S400–9. 10.1080/07315724.2011.10719983 22081685

[6] Wu L, Sun D. Meta-analysis of milk consumption and the risk of Cognitive disorders. Nutrients. 2016;8(12):824. 27999380 10.3390/nu8120824 PMC5188477

[7] Lee J, Fu Z, Chung M, Jang D-J, Lee H-J. Role of milk and dairy intake in cognitive function in older adults: a systematic review and meta-analysis. Nutr J. 2018;17(1). 10.1186/s12937-018-0387-1 PMC6112122 30149812

[8] Villoz F, Filippini T, Ortega N, Kopp-Heim D, Voortman T, Blum MR, et al. Dairy intake and risk of Cognitive decline and Dementia: a systematic review and dose-response Meta-analysis of prospective studies. Adv Nutr. 2024;15(1):100160. 38043604 10.1016/j.advnut.2023.100160 PMC10788406

[9] Anguita-Ruiz A, Aguilera CM, Gil Á. Genetics of Lactose Intolerance: an updated review and Online Interactive World maps of phenotype and genotype frequencies. Nutrients. 2020;12(9):2689. 32899182 10.3390/nu12092689 PMC7551416

[10] Fung M, Xue X, Szilagyi A. Estimating Lactase Nonpersistence distributions in the multi-ethnic Canadian demographic: a Population-based study. J Can Association Gastroenterol. 2020;3(3):103–10. 10.1093/jcag/gwy068 PMC7204802 32395684

[11] Bergholdt HK, Nordestgaard BG, Ellervik C. Milk intake is not associated with low risk of diabetes or overweight-obesity: a mendelian randomization study in 97,811 Danish individuals. Am J Clin Nutr. 2015;102(2):487–96. 26156736 10.3945/ajcn.114.105049

[12] Bergholdt HKM, Nordestgaard BG, Varbo A, Ellervik C. Milk intake is not associated with ischaemic heart disease in observational or mendelian randomization analyses in 98,529 Danish adults. Int J Epidemiol. 2015;44(2):587–603. 26085675 10.1093/ije/dyv109

[13] Davey Smith G, Ebrahim S. Mendelian randomization’: can genetic epidemiology contribute to understanding environmental determinants of disease? Int J Epidemiol. 2003;32(1):1–22. 12689998 10.1093/ije/dyg070

[14] Zhang Z, Wang M, Yuan S, Larsson SC, Liu X. Genetically predicted milk intake and risk of neurodegenerative diseases. Nutrients. 2021;13(8):2893. 34445060 10.3390/nu13082893 PMC8398304

[15] Domenighetti C, Sugier PE, Ashok Kumar Sreelatha A, Schulte C, Grover S, Mohamed O, et al. Dairy intake and Parkinson’s Disease: a mendelian randomization study. Mov Disord. 2022;37(4):857–64. 34997937 10.1002/mds.28902

[16] Yuan S, Sun J, Lu Y, Xu F, Li D, Jiang F et al. Health effects of milk consumption: phenome-wide mendelian randomization study. BMC Med. 2022;20(1). 10.1186/s12916-022-02658-w PMC9694907 36424608

[17] Preisig M, Waeber G, Vollenweider P, Bovet P, Rothen S, Vandeleur C, et al. The PsyCoLaus study: methodology and characteristics of the sample of a population-based survey on psychiatric disorders and their association with genetic and cardiovascular risk factors. BMC Psychiatry. 2009;9(1):9. 19292899 10.1186/1471-244X-9-9 PMC2667506

[18] Hofman A, Brusselle GGO, Murad SD, van Duijn CM, Franco OH, Goedegebure A, et al. The Rotterdam Study: 2016 objectives and design update. Eur J Epidemiol. 2015;30(8):661–708. 26386597 10.1007/s10654-015-0082-x PMC4579264

[19] Raina P, Wolfson C, Kirkland S, Griffith LE, Balion C, Cossette B, et al. Cohort Profile: the Canadian longitudinal study on aging (CLSA). Int J Epidemiol. 2019;48(6):1752–j3. 31633757 10.1093/ije/dyz173 PMC6929533

[20] Morabia A, Bernstein M, Kumanyika S, Sorenson A, Mabiala I, Prodolliet B, et al. [Development and validation of a semi-quantitative food questionnaire based on a population survey]. Soz Praventivmed. 1994;39(6):345–69. 7817624 10.1007/BF01299666

[21] Bernstein M, Huot I, Morabia A. Amélioration Des performances d’un questionnaire alimentaire semi-quantitatif comparé à Un Rappel Des 24 heures. Santé publique (Vandoeuvre-lès-Nancy). 1995;4:403–13.

[22] Goldbohm R, van den Brandt P, Brants H, van’t Veer PSF. Validation of a dietary questionnaire used in a large-scale prospective cohort study on diet and cancer. Eur J Clin Nutr I.994(48):253–65. 8039485

[23] Feunekes GI, Van Staveren WA, De Vries J, Burema J, Hautvast J. Relative and biomarker-based validity of a food-frequency questionnaire estimating intake of fats and cholesterol. Am J Clin Nutr. 1993;58(4):489–96. 8379504 10.1093/ajcn/58.4.489

[24] Shatenstein B, Payette H. Evaluation of the relative validity of the short Diet Questionnaire for assessing Usual Consumption frequencies of selected nutrients and foods. Nutrients. 2015;7(8):6362–74. 26247965 10.3390/nu7085282 PMC4555121

[25] Ikram MA, Brusselle G, Ghanbari M, Goedegebure A, Ikram MK, Kavousi M, et al. Objectives, design and main findings until 2020 from the Rotterdam Study. Eur J Epidemiol. 2020;35(5):483–517. 32367290 10.1007/s10654-020-00640-5 PMC7250962

[26] Koek WNH, Van Meurs JB, Van Der Eerden BC, Rivadeneira F, Zillikens MC, Hofman A, et al. The T-13910 C polymorphism in the lactase phlorizin hydrolase gene is associated with differences in serum calcium levels and calcium intake. J Bone Miner Res. 2010;25(9):1980–7. 20225268 10.1002/jbmr.83

[27] Forgetta V, Li R, Darmond-Zwaig C, Belisle A, Balion C, Roshandel D, et al. Cohort profile: genomic data for 26 622 individuals from the Canadian longitudinal study on aging (CLSA). BMJ Open. 2022;12(3):e059021. 35273064 10.1136/bmjopen-2021-059021 PMC8915305

[28] Greenland S. An introduction to instrumental variables for epidemiologists. Int J Epidemiol. 2000;29(4):722–9. 10922351 10.1093/ije/29.4.722

[29] Enattah NS, Sahi T, Savilahti E, Terwilliger JD, Peltonen L, Järvelä I. Identification of a variant associated with adult-type hypolactasia. Nat Genet. 2002;30(2):233–7. 11788828 10.1038/ng826

[30] Brumpton B, Sanderson E, Heilbron K, Hartwig FP, Harrison S, Vie GÅ et al. Avoiding dynastic, assortative mating, and population stratification biases in mendelian randomization through within-family analyses. Nat Commun. 2020;11(1). 10.1038/s41467-020-17117-4 PMC7360778 32665587

[31] Imbens GW, Angrist JD. Identification and Estimation of Local Average Treatment effects. Econometrica. 1994;62(2):467–75.

[32] Teng E. The Mental alternations Test (MAT). Clin Neuropsychol. 1995;9(3):287.

[33] Folstein MF, Folstein SE, McHugh PR. Mini-mental state. A practical method for grading the cognitive state of patients for the clinician. J Psychiatr Res. 1975;12(3):189–98. 1202204 10.1016/0022-3956(75)90026-6

[34] Huntley JD, Gould RL, Liu K, Smith M, Howard RJ. Do cognitive interventions improve general cognition in dementia? A meta-analysis and meta-regression. BMJ Open. 2015;5(4):e005247. 25838501 10.1136/bmjopen-2014-005247 PMC4390716

[35] Andrews JS, Desai U, Kirson NY, Zichlin ML, Ball DE, Matthews BR. Disease severity and minimal clinically important differences in clinical outcome assessments for Alzheimer’s disease clinical trials. Alzheimer’s & Dementia: Translational Research & Clinical Interventions. 2019;5(1):354 – 63. 10.1016/j.trci.2019.06.005 PMC6690415 31417957

[36] Salib E, McCarthy J. Mental Alternation Test (MAT): a rapid and valid screening tool for dementia in primary care. Int J Geriatr Psychiatry. 2002;17(12):1157–61. 12461766 10.1002/gps.738

[37] Stroop JR. Studies of interference in serial verbal reactions. J Exp Psychol. 1935;18(6):643–62.

[38] Troyer AK, Leach L, Strauss E, Aging. Neuropsychol Cognition. 2006;13(1):20–35. 10.1080/138255890968187 16766341

[39] Bayard S, Erkes J, Moroni C. Victoria Stroop Test: normative data in a Sample Group of Older people and the study of their clinical applications in the Assessment of Inhibition in Alzheimer’s Disease. Arch Clin Neuropsychol. 2011;26(7):653–61. 21873625 10.1093/arclin/acr053

[40] Hall J, O’Carroll RE, Frith CD. 7 - neuropsychology. In: Johnstone EC, Owens DC, Lawrie SM, McIntosh AM, Sharpe M, editors. Companion to Psychiatric studies (Eighth Edition). St. Louis: Churchill Livingstone; 2010. pp. 121–40.

[41] Grober E, Buschke H. Genuine memory deficits in dementia. Dev Neuropsychol. 1987;3(1):13–36.

[42] Loewenstein D, Acevedo A. The prospective memory test: administration and scoring manual. Miami: Unpublished Manuscript) University of Miami School of Medicine; 2004.

[43] Spreen O, Strauss E. A compendium of neuropsychological tests: Administration, norms, and commentary, 2nd ed. New York, NY, US: Oxford University Press; 1998. xvi, 736-xvi, p.

[44] Palmer TM, Sterne JAC, Harbord RM, Lawlor DA, Sheehan NA, Meng S, et al. Instrumental variable estimation of causal risk ratios and causal odds ratios in mendelian randomization analyses. Am J Epidemiol. 2011;173(12):1392–403. 21555716 10.1093/aje/kwr026

[45] Wald A. The fitting of straight lines if both variables are subject to Error. Ann Math Stat. 1940;11(3):284–300.

[46] Burgess S, Davey Smith G, Davies NM, Dudbridge F, Gill D, Glymour MM, et al. Guidelines for performing mendelian randomization investigations: update for summer 2023. Wellcome Open Res. 2019;4:186. 32760811 10.12688/wellcomeopenres.15555.1 PMC7384151

[47] Skrivankova VW, Richmond RC, Woolf BAR, Yarmolinsky J, Davies NM, Swanson SA, et al. Strengthening the reporting of Observational studies in Epidemiology using mendelian randomization: the STROBE-MR Statement. JAMA. 2021;326(16):1614–21. 34698778 10.1001/jama.2021.18236

[48] Fox J, Kleiber C, Zeileis A, Kuschnig N. Instrumental-Variables Regression by ‘2SLS’, ‘2SM’, or ‘2SMM’, with Diagnostics. 0.6-4. CRAN; 2024.

[49] Borland E, Edgar C, Stomrud E, Cullen N, Hansson O, Palmqvist S. Clinically relevant Changes for Cognitive outcomes in Preclinical and Prodromal Cognitive stages. Neurology. 2022;99(11):e1142–53. 35835560 10.1212/WNL.0000000000200817 PMC9536741

[50] Muir RT, Hill MD, Black SE, Smith EE. Minimal clinically important difference in Alzheimer’s disease: Rapid review. Alzheimer’s Dement. 2024;20(5):3352–63. 38561021 10.1002/alz.13770 PMC11095473

[51] Sonestedt E, Borné Y, Wirfält E, Ericson U. Dairy consumption, Lactase Persistence, and Mortality Risk in a Cohort from Southern Sweden. Front Nutr. 2021;8. 10.3389/fnut.2021.779034 PMC8652079 34901125

[52] Bergholdt HKM, Nordestgaard BG, Varbo A, Ellervik C. Lactase persistence, milk intake, and mortality in the Danish general population: a mendelian randomization study. Eur J Epidemiol. 2018;33(2):171–81. 29071499 10.1007/s10654-017-0328-x

[53] Haring B, Leng X, Robinson J, Johnson KC, Jackson RD, Beyth R et al. Cardiovascular Disease and Cognitive Decline in Postmenopausal Women: Results From the Women’s Health Initiative Memory Study. J Am Heart Association.2(6):e000369. 10.1161/JAHA.113.000369 PMC3886762 24351701

[54] Ortega N, Carmeli C, Efthimiou O, Beer J-H, Gunten AV, Preisig M, et al. Effect of dairy consumption on cognition in older adults: a population-based cohort study. J Nutr Health Aging. 2024;28(2):100031.38388110 10.1016/j.jnha.2023.100031

[55] Smit RAJ, Trompet S, Dekkers OM, Jukema JW, le Cessie S. Survival Bias in mendelian randomization studies: a threat to causal inference. Epidemiology. 2019;30(6):813–6. 31373921 10.1097/EDE.0000000000001072 PMC6784762

