# Supplementary material for: Dairy intake and cognitive function in older adults in three cohorts: a mendelian randomization study
Source: Nutr J. 2025 Jan 31;24:20. doi: 10.1186/s12937-025-01083-y (PMC11784005; doi:10.1186/s12937-025-01083-y)
Supplement: Supplementary file 1 — Supplementary Material 1 [file 12937_2025_1083_MOESM1_ESM.docx]

**Supplementary File 1 - Non-fermented dairy intake and cognitive function in older adults in three cohorts: a Mendelian Randomization study**

Natalia Ortega, Nick J Mueller, Abbas Dehghan, Armin von Gunten, Martin Preisig, Pedro Marques-Vidal, Marco Vinceti, Trudy Voortman, Nicolas Rodondi, Patricia O. Chocano-Bedoya

Table of Contents

Checklist 1. STROBE-nut: An extension of the STROBE statement for nutritional epidemiology2

Checklist 2. STROBE-MR checklist of recommended items to address in reports of Mendelian randomization studies7

Supplementary Table 1 Cohorts’ characteristics13

Supplementary Table 2 Cognitive tests’ characteristics14

Supplementary Table 3 Participants’ characteristics15

Supplementary Table 4 Instrument strength statistics16

Supplementary Table 5 Crude coefficients of the first and second stage models16

Supplementary Table 6 Estimates with the ratio method17

Supplementary Table 7 Sensitivity analysis with ApoE variant17

Supplementary Table 8 Sensitivity analysis without butter18

Supplementary Table 9 Sensitivity analysis assuming BMI is a mediator18

Supplementary Table 10 Sensitivity analysis adjusting for main principal components in CoLaus|PsyCoLaus19

**Table 1. STROBE-nut: An extension of the STROBE statement for nutritional epidemiology**

Lachat C et al. (2016) STrengthening the Reporting of OBservational studies in Epidemiology – Nutritional Epidemiology (STROBE-nut): an extension of the STROBE statement. Plos Medicine 13(6) <http://dx.doi.org/10.1371/journal.pmed.1002036> [pdf](http://journals.plos.org/plosmedicine/article/asset?id=10.1371%2Fjournal.pmed.1002036.PDF) or [online](http://journals.plos.org/plosmedicine/article?id=10.1371/journal.pmed.1002036) version.

| Item | Item nr | STROBE recommendations | Extension for Nutritional Epidemiology studies (STROBE-nut) | Reported on page # |
| --- | --- | --- | --- | --- |
| Title and  abstract | 1 | (a) Indicate the study’s design with a commonly used term in the title or the abstract.  (b) Provide in the abstract an informative and balanced summary of what was done and what was found. | **nut-1** State the dietary/nutritional assessment method(s) used in the title, abstract, or keywords. | 1  2 |
| Introduction |  |  |  |  |
| Background rationale | 2 | Explain the scientific background and rationale for the investigation being reported. |  | 3 |
| Objectives | 3 | State specific objectives, including any pre-specified hypotheses. |  | 3 (Lines 94-96) |
| Methods |  |  |  |  |
| Study design | 4 | Present key elements of study design early in the paper. |  | 3-4 |
| Settings | 5 | Describe the setting, locations, and relevant dates, including periods of recruitment, exposure, follow-up, and data collection. | **nut-5** Describe any characteristics of the study settings that might affect the dietary intake or nutritional status of the participants, if applicable. | 3-4 |
| Participants | 6 | a) Cohort study—Give the eligibility criteria, and the sources and methods of selection of participants. Describe methods of follow-up.  Case-control study—Give the eligibility criteria, and the sources and methods of case ascertainment and control selection. Give the rationale for the choice of cases and controls.  Cross-sectional study—Give the eligibility criteria, and the sources and methods of selection of participants.  (b) Cohort study—For matched studies, give matching criteria and number of exposed and unexposed.  Case-control study—For matched studies, give matching criteria and the number of controls per case. | **nut-6** Report particular dietary, physiological or nutritional characteristics that were considered when selecting the target population. | 3-4 |
| Variables | 7 | Clearly define all outcomes, exposures, predictors, potential confounders, and effect modifiers. Give diagnostic criteria, if applicable. | **nut-7.1** Clearly define foods, food groups, nutrients, or other food components.  **nut-7.2** When using dietary patterns or indices, describe the methods to obtain them and their nutritional properties. | 3-4-5 |
| Data sources - measurements | 8 | For each variable of interest, give sources of data and details of methods of assessment (measurement). Describe comparability of assessment methods if there is more than one group. | **nut-8.1** Describe the dietary assessment method(s), e.g., portion size estimation, number of days and items recorded, how it was developed and administered, and how quality was assured. Report if and how supplement intake was assessed.  **nut-8.2** Describe and justify food composition data used. Explain the procedure to match food composition with consumption data. Describe the use of conversion factors, if applicable.  **nut-8.3** Describe the nutrient requirements, recommendations, or dietary guidelines and the evaluation approach used to compare intake with the dietary reference values, if applicable.  **nut-8.4** When using nutritional biomarkers, additionally use the STROBE Extension for Molecular Epidemiology (STROBE-ME). Report the type of biomarkers used and their usefulness as dietary exposure markers.  **nut-8.5** Describe the assessment of nondietary data (e.g., nutritional status and influencing factors) and timing of the assessment of these variables in relation to dietary assessment.  **nut-8.6** Report on the validity of the dietary or nutritional assessment methods and any internal or external validation used in the study, if applicable. | 4 |
| Bias | 9 | Describe any efforts to address potential sources of bias. | **nut-9** Report how bias in dietary or nutritional assessment was addressed, e.g., misreporting, changes in habits as a result of being measured, or data imputation from other sources | 6 |
| Study Size | 10 | Explain how the study size was arrived at. |  | NA |
| Quantitative variables | 11 | Explain how quantitative variables were handled in the analyses. If applicable, describe which groupings were chosen and why. | **nut-11** Explain categorization of dietary/nutritional data (e.g., use of N-tiles and handling of nonconsumers) and the choice of reference category, if applicable. | 4 |
| Statistical  Methods | 12 | (a) Describe all statistical methods, including those used to control for confounding  (b) Describe any methods used to examine subgroups and interactions.  (c) Explain how missing data were addressed.  (d) Cohort study—If applicable, explain how loss to follow-up was addressed.  Case-control study—If applicable, explain how matching of cases and controls was addressed.  Cross-sectional study—If applicable, describe analytical methods taking account of sampling strategy.  (e) Describe any sensitivity analyses. | **nut-12.1** Describe any statistical method used to combine dietary or nutritional data, if applicable.  **nut-12.2** Describe and justify the method for energy adjustments, intake modeling, and use of weighting factors, if applicable.  **nut-12.3** Report any adjustments for measurement error, i.e,. from a validity or calibration study. | 6 |
| Results |  |  |  |  |
| Participants | 13 | (a) Report the numbers of individuals at each stage of the study—e.g., numbers potentially eligible, examined for eligibility, confirmed eligible, included in the study, completing follow-up, and analyzed.  (b) Give reasons for non-participation at each stage.  (c) Consider use of a flow diagram. | **nut-13** Report the number of individuals excluded based on missing, incomplete or implausible dietary/nutritional data. | 6 |
| Descriptive data | 14 | (a) Give characteristics of study participants (e.g., demographic, clinical, social) and information on exposures and potential confounders  (b) Indicate the number of participants with missing data for each variable of interest  (c) Cohort study—Summarize follow-up time (e.g., average and total amount) | **nut-14** Give the distribution of participant characteristics across the exposure variables if applicable. Specify if food consumption of total population or consumers only were used to obtain results. | 6 to 8 |
| Outcome data | 15 | Cohort study—Report numbers of outcome events or summary measures over time.  Case-control study—Report numbers in each exposure category, or summary measures of exposure.  Cross-sectional study—Report numbers of outcome events or summary measures. |  | 10 |
| Main results | 16 | (a) Give unadjusted estimates and, if applicable, confounder-adjusted estimates and their precision (e.g., 95% confidence interval).  Make clear which confounders were adjusted for and why they were included.  (b) Report category boundaries when continuous variables were categorized.  (c) If relevant, consider translating estimates of relative risk into absolute risk for a meaningful time period. | **nut-16** Specify if nutrient intakes are reported with or without inclusion of dietary supplement intake, if applicable. | 10 |
| Other analyses | 17 | Report other analyses done—e.g., analyses of subgroups and interactions and sensitivity analyses. | **nut-17** Report any sensitivity analysis (e.g., exclusion of misreporters or outliers) and data imputation, if applicable. | 11 |
| Discussion |  |  |  |  |
| Key results | 18 | Summarize key results with reference to study objectives. |  | 11 |
| Limitation | 19 | Discuss limitations of the study, taking into account sources of potential bias or imprecision. Discuss both direction and magnitude of any potential bias. | **nut-19** Describe the main limitations of the data sources and assessment methods used and implications for the interpretation of the findings. | 12-13 |
| Interpretation | 20 | Give a cautious overall interpretation of results considering objectives, limitations, multiplicity of analyses, results from similar studies, and other relevant evidence. | **nut-20** Report the nutritional relevance of the findings, given the complexity of diet or nutrition as an exposure. | 13 |
| Generalizability | 21 | Discuss the generalizability (external validity) of the study results. |  | 12-13 |
| Other information |  |  |  |  |
| Funding | 22 | Give the source of funding and the role of the funders for the present study and, if applicable, for the original study on which the present article is based. |  | 13 |
| *Ethics* |  |  | **nut-22.1** Describe the procedure for consent and study approval from ethics committee(s). | 4 |
| *Supplementary material* |  |  | **nut-22.2** Provide data collection tools and data as online material or explain how they can be accessed. | Yes |

**Table 2. STROBE-MR checklist of recommended items to address in reports of Mendelian randomization studies** ^1^ ^2^

| **Item No.** | **Section** | **Checklist item** | **Page No.** | **Relevant text from manuscript** |
| --- | --- | --- | --- | --- |
| 1 | **TITLE and ABSTRACT** | Indicate Mendelian randomization (MR) as the study’s design in the title and/or the abstract if that is a main purpose of the study | 1 | Dairy intake and cognitive function in older adults in three cohorts: a Mendelian Randomization study |
|  | **INTRODUCTION** |  |  |  |
| 2 | **Background** | Explain the scientific background and rationale for the reported study. What is the exposure? Is a potential causal relationship between exposure and outcome plausible? Justify why MR is a helpful method to address the study question | 3 | Lines 78-93 |
| 3 | **Objectives** | State specific objectives clearly, including pre-specified causal hypotheses (if any). State that MR is a method that, under specific assumptions, intends to estimate causal effects | 3 | Lines 94-96 |
|  | **METHODS** |  |  |  |
| 4 | **Study design and data sources** | Present key elements of the study design early in the article. Consider including a table listing sources of data for all phases of the study. For each data source contributing to the analysis, describe the following: |  |  |
|  | a) | Setting: Describe the study design and the underlying population, if possible. Describe the setting, locations, and relevant dates, including periods of recruitment, exposure, follow-up, and data collection, when available. | 4 | Lines 99-106 |
|  | b) | Participants: Give the eligibility criteria, and the sources and methods of selection of participants. Report the sample size, and whether any power or sample size calculations were carried out prior to the main analysis | 4 | Lines 99-106 |
|  | c) | Describe measurement, quality control and selection of genetic variants | 4 | Lines 123-135 |
|  | d) | For each exposure, outcome, and other relevant variables, describe methods of assessment and diagnostic criteria for diseases | 5 | Lines 150-170 |
|  | e) | Provide details of ethics committee approval and participant informed consent, if relevant | 3 | Lines 107-110 |
| 5 | **Assumptions** | Explicitly state the three core IV assumptions for the main analysis (relevance, independence and exclusion restriction) as well assumptions for any additional or sensitivity analysis | 4/5 | Lines 136-149 and Lines 172-183 |
| 6 | **Statistical methods: main analysis** | Describe statistical methods and statistics used |  |  |
|  | a) | Describe how quantitative variables were handled in the analyses (i.e., scale, units, model) | 6 | Lines 192-198 |
|  | b) | Describe how genetic variants were handled in the analyses and, if applicable, how their weights were selected | 6 | Lines 192-198 |
|  | c) | Describe the MR estimator (e.g. two-stage least squares, Wald ratio) and related statistics. Detail the included covariates and, in case of two-sample MR, whether the same covariate set was used for adjustment in the two samples | 6 | Lines 192-198 |
|  | d) | Explain how missing data were addressed |  |  |
|  | e) | If applicable, indicate how multiple testing was addressed | NA | NA |
| 7 | **Assessment of assumptions** | Describe any methods or prior knowledge used to assess the assumptions or justify their validity | 6 | Lines 199-211 |
| 8 | **Sensitivity analyses and additional analyses** | Describe any sensitivity analyses or additional analyses performed (e.g. comparison of effect estimates from different approaches, independent replication, bias analytic techniques, validation of instruments, simulations) | 6 | Lines 199-211 |
| 9 | **Software and pre-registration** |  |  |  |
|  | a) | Name statistical software and package(s), including version and settings used | 6 | Lines 213-215 |
|  | b) | State whether the study protocol and details were pre-registered (as well as when and where) | NA | NA |
|  | **RESULTS** |  |  |  |
| 10 | **Descriptive data** |  |  |  |
|  | a) | Report the numbers of individuals at each stage of included studies and reasons for exclusion. Consider use of a flow diagram | 6 | Line 217 |
|  | b) | Report summary statistics for phenotypic exposure(s), outcome(s), and other relevant variables (e.g. means, SDs, proportions) | 6/7 | Lines 218-228 (Table 1) |
|  | c) | If the data sources include meta-analyses of previous studies, provide the assessments of heterogeneity across these studies | NA | NA |
|  | d) | For two-sample MR:  i.  Provide justification of the similarity of the genetic variant-exposure associations between the exposure and outcome samples  ii.  Provide information on the number of individuals who overlap between the exposure and outcome studies | NA | NA |
| 11 | **Main results** |  |  |  |
|  | a) | Report the associations between genetic variant and exposure, and between genetic variant and outcome, preferably on an interpretable scale | 10 | Lines 1-5 (Table 2) |
|  | b) | Report MR estimates of the relationship between exposure and outcome, and the measures of uncertainty from the MR analysis, on an interpretable scale, such as odds ratio or relative risk per SD difference | 10 | Lines 13-19 (Table 3) - RD |
|  | c) | If relevant, consider translating estimates of relative risk into absolute risk for a meaningful time period | 10 | Table 3 |
|  | d) | Consider plots to visualize results (e.g. forest plot, scatterplot of associations between genetic variants and outcome versus between genetic variants and exposure) | NA | NA |
| 12 | **Assessment of assumptions** | |  |  |
|  | a) | Report the assessment of the validity of the assumptions | 11 | Lines 26-38 |
|  | b) | Report any additional statistics (e.g., assessments of heterogeneity across genetic variants, such as *I^2^*, Q statistic or E-value) | NA | NA |
| 13 | **Sensitivity analyses and additional analyses** | |  |  |
|  | a) | Report any sensitivity analyses to assess the robustness of the main results to violations of the assumptions | 11 | Lines 26-38 |
|  | b) | Report results from other sensitivity analyses or additional analyses | 11 | Lines 26-38 |
|  | c) | Report any assessment of direction of causal relationship (e.g., bidirectional MR) | NA | NA |
|  | d) | When relevant, report and compare with estimates from non-MR analyses | 12 | Lines 74-79 |
|  | e) | Consider additional plots to visualize results (e.g., leave-one-out analyses) | NA | NA |
|  | **DISCUSSION** |  |  |  |
| 14 | **Key results** | Summarize key results with reference to study objectives | 11 | Lines 41-44 |
| 15 | **Limitations** | Discuss limitations of the study, taking into account the validity of the IV assumptions, other sources of potential bias, and imprecision. Discuss both direction and magnitude of any potential bias and any efforts to address them | 12/13 | Lines 97-134 |
| 16 | **Interpretation** |  |  |  |
|  | a) | Meaning: Give a cautious overall interpretation of results in the context of their limitations and in comparison with other studies | 12 | Lines 65-79 |
|  | b) | Mechanism: Discuss underlying biological mechanisms that could drive a potential causal relationship between the investigated exposure and the outcome, and whether the gene-environment equivalence assumption is reasonable. Use causal language carefully, clarifying that IV estimates may provide causal effects only under certain assumptions | 3 | Lines 67-77 |
|  | c) | Clinical relevance: Discuss whether the results have clinical or public policy relevance, and to what extent they inform effect sizes of possible interventions | 11 | Lines 45-64 |
| 17 | **Generalizability** | Discuss the generalizability of the study results (a) to other populations, (b) across other exposure periods/timings, and (c) across other levels of exposure | 11/12 | Lines 61-64 and 97-107 |
|  | **OTHER INFORMATION** |  |  |  |
| 18 | **Funding** | Describe sources of funding and the role of funders in the present study and, if applicable, sources of funding for the databases and original study or studies on which the present study is based | 13 | Lines 140-146 |
| 19 | **Data and data sharing** | Provide the data used to perform all analyses or report where and how the data can be accessed, and reference these sources in the article. Provide the statistical code needed to reproduce the results in the article, or report whether the code is publicly accessible and if so, where | 14 | Lines 172-191 |
| 20 | **Conflicts of Interest** | All authors should declare all potential conflicts of interest | 14 | Line 166 |

This checklist is copyrighted by the Equator Network under the Creative Commons Attribution 3.0 Unported (CC BY 3.0) license.

1. Skrivankova VW, Richmond RC, Woolf BAR, Yarmolinsky J, Davies NM, Swanson SA, et al. Strengthening the Reporting of Observational Studies in Epidemiology using Mendelian Randomization (STROBE-MR) Statement. JAMA. 2021;under review.

2. Skrivankova VW, Richmond RC, Woolf BAR, Davies NM, Swanson SA, VanderWeele TJ, et al. Strengthening the Reporting of Observational Studies in Epidemiology using Mendelian Randomisation (STROBE-MR): Explanation and Elaboration. BMJ. 2021;375:n2233.

**Supplementary Table 1.** Description of the eligible population, recruitment strategies, participation rates, recruitment dates and follow-ups of PsyCoLaus|CoLaus, Rotterdam Study and the Canadian Longitudinal Study on Aging.

| Cohort | Eligible population characteristics | Recruitment strategies | Participation rates | Period baseline recruitment | Follow-ups |
| --- | --- | --- | --- | --- | --- |
| CoLaus\|  PsyCoLaus | Living in Lausanne, 35-75 years old | Random sample contacted by phone and mailing | 41% (n=6,734) | 2003-2006 | 2^nd^ follow-up: 2014-2018 |
| Rotterdam Study | Cohort I: >55 years old in the Ommoord district in Rotterdam  Cohort II: movers into the area  Cohort III: younger participants 45-54 years old in the area | All inhabitants in the area were invited to participate by mail | 72% (14,926) | Cohort I: 1990  Cohort II: 2000  Cohort III:  2006 | Cohort I and II: waves 6 and 7 2014-2016  Cohort III: waves 2 and 3 (2012-14) |
| CLSA (1) | Households with adults 45-85 years old | Random sample of households recruited in a multi-stage process | 45% (n=30,097) | 2010-2015 | 2015-2018 |

CLSA: Canadian Longitudinal Study on Aging

**Supplementary Table 2.** Cognitive test description and range score by cognitive domain.

| **Cognitive domain** | **Cognitive test definition** | **Range score** |
| --- | --- | --- |
| *General cognitive function* | | |
| Mental Alternation Test (MAT)  *CLSA* | Cognitive switching exercise in which participants switch between the numbers 1-26 and the letters of the alphabet (1-A, 2-B, 3-C) and finishes after 30 seconds (2, 3). | 0-52 |
| Mini Mental State Examination (MMSE)  *PsyCoLaus, RS* | The first part focuses on verbal responses and evaluates orientation, memory, calculation and attention. The second part examines abilities such as naming, following verbal and written instructions, spontaneous sentence writing and copying a complex figure. There is no time limit (4). | 0-30 |
| *Verbal fluency* | | |
| Animal naming *CLSA, PsyCoLaus, RS* | Individuals mention as many animals as possible in 60 seconds (CLSA, RS) or 2 min (PsyCoLaus) (5). | CLSA: 0-44, PsyCoLaus: 3-58, RS: 5-44 |
| *Memory* | | |
| Time and event-based prospective memory tests  *CLSA* | The task evaluates the capacity to recall and execute a planned action at a specific time or in response to a known event. By using cues after 15 or 30 minutes, the test battery includes both event-based and time-based prospective memory tasks. The final score is added together by three criteria: intention to perform, accuracy of response and the need for reminders (6). | 0-9 |
| Free and Cued Selective Reminding Test (FCSRT)  *PsyCoLaus* | Individuals are tasked with identifying items based on category cues. During the testing phase, they have to recall the items they were taught (free recall). Category cues help with remembering items which were not mentioned during free call. The combined score of free and cued recall is called the total recall (7). | 0-48 |
| *Processing speed and execute function* | | |
| Stroop  *CLSA, PsyCoLaus, RS* | The test has three tasks. First, participants must identify the color of the ink in which dots are printed on a card. Second, they name the ink color of non-color words printed on a card. Third, they must name the color of the ink in which the color words are without reading actual word. The score is based on the difference in the time taken to complete task 3 and task 1 adjusted for the number of errors (2, 8, 9). | CLSA: 0-211 s |
| *Verbal learning* | | |
| Rey Auditory verbal learning test (RAVLT)  *CLSA, RS* | In the original test, a set of 15 words are read aloud by the examiner at a pace of one word per second and then the participants are asked to repeat as many words as they remember. This process is repeated five times. Then, a different set of 15 words is presented to the patients for a single recall attempt. Immediately afterwards, the patients are tasked with recalling all the words from the initial list. For CLSA, only a single measurement was used instead of five, and a delayed recall after 5 minutes was added (10). | CLSA: 0-8  RS: 0-15 |

**Supplementary Table 3.** Baseline characteristics of the study population without stratification.

|  | PsyCoLaus (n=1,565) | Rotterdam Study (n=8,382) | CLSA  (n=29,046) |
| --- | --- | --- | --- |
| Sex - Male (%) | 664 (42.4) | 3527 (42.1) | 14182 (48.8) |
| Age (%) |  |  |  |
| Below 70 | 948 (60.6) | 2476 (59.4) | 21073 (72.6) |
| Between 70 and <75 | 356 (22.7) | 1108 (26.6) | 2927 (10.1) |
| 75 and above | 261 (16.7) | 584 (14.0) | 5046 (17.4) |
| Education (%) |  |  |  |
| Basic | 1081 (69.1) | 4741 (56.9) | 2142 (8.7) |
| Secondary | 251 (16.0) | 2336 (28.0) | 9424 (38.3) |
| Higer | 233 (14.9) | 1261 (15.1) | 13058 (53.0) |
| BMI (%) |  |  |  |
| Normal (BMI <25) | 594 (38.4) | 1683 (29.3) | 8763 (30.3) |
| Overweight (BMI 25-29.9) | 641 (41.5) | 2669 (46.5) | 11661 (40.3) |
| Obese (BMI >30) | 311 (20.1) | 1383 (24.1) | 8495 (29.4) |
| Smoking (%) |  |  |  |
| Current | 229 (14.7) | 842 (17.4) | 2602 (9.0) |
| Former | 683 (43.8) | 2428 (50.2) | 12669 (43.6) |
| Never | 648 (41.5) | 1569 (32.4) | 13774 (47.4) |
| CV event* (%) | 318 (20.4) | 168 (2.0) | 3566 (12.3) |
| Hypertension (%) | 1056 (67.6) | 3299 (70.4) | 10685 (36.9) |
| Diabetes (%) | 186 (11.9) | 1288 (19.3) | 5122 (17.7) |
| Physical Activity (%) |  |  |  |
| Low | 623 (45.6) | 1393 (34.0) | 9407 (33.9) |
| Medium | 475 (34.8) | 1345 (32.8) | 9148 (32.9) |
| High | 268 (19.6) | 1361 (33.2) | 9233 (33.2) |
| MMSE/MAT (mean (SD)) | 29.4 (1.2) | 27.8 (2.0) | 26.6 (8.7) |

PsyCoLaus: CoLaus|PsyCoLaus, CLSA: Canadian Longitudinal Study on Aging; CV: Cardiovascular; MMSE: Mini-Mental State Examination; MAT: Mental Alternation Test. *In PsyCoLaus and CLSA includes self-reported cardiomyopathy, congenital heart disease, valvular heart disease, heart failure, coronary artery disease, angina, myocardial infarction, stroke, percutaneous coronary intervention, coronary artery bypass graft or pacing. In Rotterdam Study includes stroke events.

**Supplementary Table 4.** Descriptive statistics on relevance assumption between lactase persistence and total dairy and non-fermented dairy.

|  | **PsyCoLaus** | **p-value** | **Rotterdam Study** | **p-value** | **CLSA** | **p-value** |
| --- | --- | --- | --- | --- | --- | --- |
| **Total dairy** | | | | | | |
| R^2^ | -0.05 | 1.5x10^-5^ | -0.16 | 2x10^-16^ | -0.17 | 2x10^-16^ |
| F statistic | 1.84 | 0.18 | 5.0 | 0.03 | 128.7 | 2x10^-16^ |
| Wu-Hausman | 0.16 | 0.694 | 1.4 | 0.23 | 44.1 | 3.2x10^-11^ |
| **Non-fermented dairy** | | | | | | |
| R^2^ | -0.06 | 7.8x10^-6^ | -0.27 | 2x10^-16^ | -0.19 | 2.2x10^-16^ |
| F statistic | 5.47 | 0.02 | 3.6 | 0.06 | 121.1 | 2x10^-16^ |
| Wu-Hausman | 0.185 | 0.67 | 1.43 | 0.23 | 43.5 | 4.4x10^-3^ |
| **Fermented dairy** | | | | | | |
| R^2^ | 0.01 | 0.92 | 0.0002 | 0.26 | 0.02 | 8x10^-5^ |
| F statistic | 1.07 | 0.30 | 1.85 | 0.17 | 3.1 | 0.08 |
| Wu-Hausman | 0.21 | 0.65 | 1.47 | 0.23 | 40.19 | 1.6x10^-10^ |

**Supplementary Table 5.** Crude coefficients (95% CI) for lactase persistence and cognitive function, total and non-fermented dairy and cognitive function.

| **Lactase persistence** | **PsyCoLaus** | **Rotterdam Study** | **CLSA** |
| --- | --- | --- | --- |
| MMSE/MAT | -0.04 (-0.33, 0.24) | -0.08 (-0.34, 0.18) | 0.77 (0.49, 1.06) |
| Executive function | 0.16 (-0.14, 0.46) | 1.32 (-1.45, 4.09) | 0.76 (0.56, 0.95) |
| Verbal fluency | -0.03 (-1.45, 1.40) | 0.23 (-0.56, 1.03) | 0.61 (0.44, 0.78) |
| Verbal learning | - | 0.15 (-0.23, 0.36) | 0.19 (0.11, 0.27) |
| Memory | -0.60 (-1.54, 0.33) | - | 0.16 (0.12, 0.21) |
| **Total dairy** |  |  |  |
| MMSE/MAT | 0.03 (-0.003, 0.06) | 0.002 (-0.01, 0.02) | -0.19 (-0.25, -0.12) |
| Executive function | -0.01 (-0.04, 0.03) | 0.05 (-0.09, 0.20) | -0.09 (-0.14, -0.05) |
| Verbal fluency | -0.07 (-0.22, 0.09) | 0.01 (-0.03, 0.05) | -0.08 (-0.12, -0.05) |
| Verbal learning | - | 0.01 (-0.0, 0.03) | -0.06 (-0.07, -0.04) |
| Memory | -0.06 (-0.16, 0.05) | - | -0.01 (-0.02, 0.01) |
| **Non-fermented dairy** | **PsyCoLaus** | **Rotterdam Study** | **CLSA** |
| MMSE/MAT | 0.005 (-0.05, 0.06) | 0.002 (-0.01, 0.02) | -0.29 (-0.38, -0.21) |
| Executive function | 0.02 (-0.03, 0.07) | 0.10 (-0.06, 0.27) | -0.14 (-0.15, -0.10) |
| Verbal fluency | -0.08 (-0.33, 0.17) | -0.01 (-0.06, 0.04) | -0.16 (-0.19, -0.09) |
| Verbal learning | - | 0.002 (-0.02, 0.02) | -0.12 (-0.22, 0.10) |
| Memory | -0.01 (-0.18, 0.15) | - | -0.01 (-0.02, 0.001) |

CLSA: Canadian Longitudinal Study on Aging, MMSE: Mini- Mental state examination, MAT: Mental Alternation Test.

**Supplementary Table 6.** Point estimates with the ratio method in comparison to 2SLS.

|  | CoLaus\|PsyCoLaus (n=1565) | | Rotterdam Study (n=8382) | | CLSA  (n=29046) | |
| --- | --- | --- | --- | --- | --- | --- |
|  | Ratio method | 2SLS | Ratio method | 2SLS | Ratio method | 2SLS |
| Total dairy | | | | | | |
| MMSE/MAT | 0.11 | 0.18 (-0.65 to 1.0) | -0.17 | -0.20 (-0.56 to 0.17) | 3.26 | 3.38 (2.11 to 4.65) |
| Executive function | 0.33 | 0.88 (-2.10 to 3.86) | 2.79 | 3.80 (-1.68 to 9.28) | 2.88 | 3.05 (2.09 to 4.00) |
| Verbal fluency | 0.67 | 1.65 (-6.07 to 9.37) | 0.55 | 0.77 (-0.67 to 2.21) | 2.09 | 2.03 (1.30 to 2.76) |
| Verbal learning | - | - | 0.15 | 0.21 (-0.28 to 0.70) | 0.82 | 0.87 (0.53 to 1.21) |
| Memory | -0.66 | -1.60 (-7.4 to 4.18) | - | - | 0.68 | 0.67 (0.47 to 0.88) |
| Non-fermented dairy | | | | | | |
| MMSE/MAT | 0.2 | 0.2 (-0.6 to 1.0) | -0.2 | -0.3 (-0.8 to 0.3) | 4.2 | 4.4 (2.8 to 6.0) |
| Executive function | 0.5 | 0.5 (-0.4 to 1.0) | 3.9 | 6.7 (-5.9 to 19.3) | 2.7 | 2.7 (1.8 to 3.7) |
| Verbal fluency | 1.1 | 1.0 (-2.7 to 4.6) | 0.8 | 1.2 (-1.4 to 3.8) | 3.8 | 4.1 (2.9 to 5.2) |
| Verbal learning | - | - | 0.2 | 0.3 (-0.5 to 1.1) | 1.0 | 1.1 (0.7 to 1.6) |
| Memory | -1.1 | -1.0 (-3.6 to 1.7) | - | - | 0.9 | 0.9 (0.7 to 1.2) |

CLSA: Canadian Longitudinal Study on Aging, MMSE: Mini- Mental state examination, MAT: Mental Alternation Test.

**Supplementary Table 7.** Sensitivity analysis with ApoE variant A) Mean consumption of non-fermented dairy by ApoE4 variant, and B) Mean difference in cognitive function with the ApoE4 variant. PsyCoLaus: CoLaus|PsyCoLaus, CLSA: Canadian Longitudinal Study on Aging, MMSE: Mini- Mental state examination, MAT: Mental Alternation Test.

**A**

|  | Non-fermented dairy intake (g/d) (SE) | | |
| --- | --- | --- | --- |
|  | ApoE | Non-ApoE | Difference |
| PsyCoLaus | 91.2 (5.5) | 81.0 (3.2) | 10.2 (6.4) |
| CLSA* | 1.02 (0.01) | 0.99 (0.007) | 0.03 (0.01) |

*times/d - CLSA performed semiquantitative FFQs

**B**

|  | PsyCoLaus (n=1186) | CLSA (n= 19863) |
| --- | --- | --- |
| MMSE/MAT | -0.13 (-0.42, 0.16) | -0.41 (-0.63, -0.18) |
| Verbal fluency | 0.02 (-1.35, 1.40) | -0.05 (-0.20, 0.10) |
| Excecutive function test | 0.06 (-0.24, 0.35) | 0.20 (0.06, 0.35) |
| Memory | -0.32 (-1.26, 0.62) | -0.08 (-0.12, -0.04) |

CLSA: Canadian Longitudinal Study on Aging, MMSE: Mini- Mental state examination, MAT: Mental Alternation Test.

**Supplementary Table 8.** Sensitivity analysis of the two-stage least squares regression models without including butter as part of the non-fermented dairy.

|  | Theoretical Range scores | PsyCoLaus  (n=1565) | Rotterdam Study  (n=8382) | CLSA  (n=29046) |
| --- | --- | --- | --- | --- |
| MMSE/MAT | 0-30/0-52 | 0.18 (-0.61 to 0.97) | -0.29 (-0.84 to 0.27) | 4.60 (2.96 to 6.23) |
| Executive function | difference in seconds | 0.5 (-0.43 to 1.44) | 6.71 (-5.91 to 19.33) | 2.84 (1.86 to 3.81) |
| Verbal fluency | 0-58 words | 0.96 (-0.05 to 4.64) | 1.21 (-1.35 to 3.76) | 4.11 (2.91 to 5.31) |
| Verbal learning | 0-8 in CLSA  0-15 in CLSA | - | 0.32 (-0.49 to 1.14) | 1.18 (0.74 to 1.62) |
| Memory | FCSRT in PsyCoLaus 0-48  PMT in CLSA 0-9 | -0.07 (-0.98 to 1.7) | - | 0.93 (0.67 to 1.19) |

CLSA: Canadian Longitudinal Study on Aging, MMSE: Mini- Mental state examination, MAT: Mental Alternation Test.

**Supplementary Table 9.** Sensitivity analysis of all the two least squares regression models excluding BMI, assuming it might mediate the relationship between dairy consumption and cognitive function.

|  | Dairy type | PsyCoLaus  (n=1565) | Rotterdam Study  (n=8382) | CLSA  (n=29046) |
| --- | --- | --- | --- | --- |
| MMSE/MAT | Total | 0.17 (-0.65 to 0.99) | -0.19 (-0.55 to 0.17) | 3.27 (2.02 to 4.52) |
| 0-30/0-52 | Non-fermented | 0.16 (-0.6 to 0.92) | -0.27 (-0.81 to 0.26) | 3.36 (2.14 to 4.57) |
| Executive function | Total | 0.78 (-1.90 to 3.47) | 3.6 5(-1.64 to 8.93) | 3.02 (2.07 to 3.96) |
| difference in seconds | Non-fermented | 0.44 (-0.44 to 1.32) | 5.52 (-3.69 to 14.73) | 2.08 (1.35 to 2.81) |
| Verbal fluency | Total | 1.17 (-5.87 to 8.2) | 0.80 (-0.63 to 2.23) | 1.96 (1.24 to 2.68) |
| 0-58 words | Non-fermented | 0.67 (-0.06 to 4.23) | 1.07 (-0.96 to 3.08) | 3.02 (2.16 to 3.89) |
| Verbal learning | Total | - | 0.23 (-0.26 to 0.73) | 0.83 (0.50 to 1.16) |
| 0-8 in CLSA  0-15 in CLSA | Non-fermented | - | 0.28 (-0.33 to 0.90) | 0.84 (0.52 to 1.16) |
| Memory | Total | -1.01 (-3.64 to 1.63) | - | 0.68 (0.47 to 0.88) |
| FCSRT in PsyCoLaus 0-48  PMT in CLSA 0-9 | Non-fermented | -0.07 (-1.01 to 1.63) | - | 0.7 (0.51 to 0.90) |

CLSA: Canadian Longitudinal Study on Aging, MMSE: Mini- Mental state examination, MAT: Mental Alternation Test.

**Supplementary Table 10.** Sensitivity analysis of the two-stage least squares regression models using the first four principal components instead of height to control for population stratification in PsyCoLaus (n=1565).

|  | Dairy type | Score difference |
| --- | --- | --- |
| MMSE/MAT | Total | 0.02 (-0.85 to 0.90) |
| 0-30/0-52 | Non-fermented | 0.02 (-0.71 to 0.74) |
| Executive function | Total | 0.51 (-1.22 to 2.24) |
| difference in seconds | Non-fermented | 0.32 (-0.46 to 1.10) |
| Verbal fluency | Total | 0.67 (-5.25 to 6.60) |
| 0-58 words | Non-fermented | 0.41 (-0.06 to 3.88) |
| Memory | Total | -0.63 (-4.41 to 3.15) |
| FCSRT 0-48 | Non-fermented | -0.42 (-0.06 to 1.96) |

**References**

1. Raina P, Wolfson C, Kirkland S, Griffith LE, Balion C, Cossette B, et al. Cohort Profile: The Canadian Longitudinal Study on Aging (CLSA). International Journal of Epidemiology. 2019;48(6):1752-3j.

2. Tuokko H, Griffith LE, Simard M, Taler V. Cognitive measures in the Canadian Longitudinal Study on Aging. Clin Neuropsychol. 2017;31(1):233-50.

3. Teng E. The Mental Alternations Test (MAT). The Clinical Neuropsychologist. 1995;9(3):287.

4. Folstein MF, Folstein SE, McHugh PR. “Mini-mental state”: A practical method for grading the cognitive state of patients for the clinician. Journal of Psychiatric Research. 1975;12(3):189-98.

5. Tuokko H, Griffith LE, Simard M, Taler V. Cognitive measures in the Canadian Longitudinal Study on Aging. The Clinical Neuropsychologist. 2017;31(1):233-50.

6. Loewenstein D, Acevedo A. The prospective memory test: Administration and scoring manual. Unpublished Manuscript) University of Miami School of Medicine, Miami. 2004.

7. Grober E, Buschke H. Genuine memory deficits in dementia. Developmental Neuropsychology. 1987;3(1):13-36.

8. Troyer AK, Leach L, Strauss E. Aging and Response Inhibition: Normative Data for the Victoria Stroop Test. Aging, Neuropsychology, and Cognition. 2006;13(1):20-35.

9. Bayard S, Erkes J, Moroni C. Victoria Stroop Test: Normative Data in a Sample Group of Older People and the Study of Their Clinical Applications in the Assessment of Inhibition in Alzheimer's Disease. Archives of Clinical Neuropsychology. 2011;26(7):653-61.

10. Hall J, O'Carroll RE, Frith CD. 7 - Neuropsychology. In: Johnstone EC, Owens DC, Lawrie SM, McIntosh AM, Sharpe M, editors. Companion to Psychiatric Studies (Eighth Edition). St. Louis: Churchill Livingstone; 2010. p. 121-40.
