# Supplementary material for: Dairy intake and cognitive function in older adults in three cohorts: a mendelian randomization study
Source: Nutr J. 2025 Jan 31;24:20. doi: 10.1186/s12937-025-01083-y (PMC11784005; doi:10.1186/s12937-025-01083-y)
Supplement: Supplementary file 2 — Supplementary Material 2 [file 12937_2025_1083_MOESM2_ESM.html]

OrtegaMueller\_MR\_SupMaterials2


# Non-fermeted dairy and cognitive function¶

### Example code from Rotterdam Study and CoLaus|PsyCoLaus¶

### 0. Loading packages¶

In [21]:

```
library(tidyverse)
library(MASS)
library(ggpubr)
library(tableone)
library(gridExtra)
library(rlang)
library(survey)
library(ivreg)

#Ignore or not warnings
options(warn=-1)
dodge = position_dodge(width=0.1)
```

### 1. CoLaus|PsyCoLaus¶

#### 1A. Loading datasets¶

In [22]:

```
baseline <- read.csv (".../Baseline.csv")
fu1 <- read.csv (".../FU1.csv")
fu2 <- read.csv (".../FU2.csv")
fu3 <- read.csv (".../FU3.csv")
cog <- read.csv (".../20220908_Chocano_Cogn_F0F1F2F3.csv")
snp <- read.csv(".../SNP.csv")
possnp <- read.delim(".../request_09-Apr-2024-13h37_HRCimputed_data.txt")
pcs <- read.csv(".../Baseline_PCs.csv")
```

#### 1B. Recoding exposure outcome and covariates¶

In [23]:

```
#EXPOSURE

plot(snp$rs4988235)
snp$lp <- ifelse(snp$rs4988235 < 0.5, 0, 1)
table(snp$lp) #23% NP
possnp$apoe <- ifelse(possnp$rs429358 < 0.5, 0, 1)

df <- dplyr::full_join(baseline, fu1, by = c("pt"))
df <- df %>% 
    dplyr::full_join(fu2, by = c("pt")) %>%
    dplyr::full_join(fu3, by = c("pt")) %>%
    dplyr::full_join(cog, by = c("pt")) %>%
    dplyr::full_join(snp, by = c("pt")) %>%
    dplyr::full_join(possnp, by=c("pt")) %>%
    dplyr::full_join(pcs, by=c("pt"))

df <- df %>%
        mutate(total_dairy_b = F1FFQ1amount+F1FFQ2amount+F1FFQ3amount+F1FFQ4amount+F1FFQ5amount+F1FFQ6amount+F1FFQ7amount+
                    F1FFQ8amount+F1FFQ52amount+F1FFQ53amount+F1FFQ71amount+F1FFQ68amount+F1FFQ82amount+F1FFQ83amount+
                    F1FFQ84amount+F1FFQ85amount+F1FFQ86amount+F1FFQ63amount,
                nobutterferm_dairy_b = F1FFQ53amount+F1FFQ71amount+F1FFQ68amount+F1FFQ82amount+F1FFQ83amount+F1FFQ84amount+
               F1FFQ85amount+F1FFQ86amount+F1FFQ63amount,
                nonferm_dairy_b = F1FFQ52amount+F1FFQ53amount+F1FFQ71amount+F1FFQ68amount+F1FFQ82amount+F1FFQ83amount+
                    F1FFQ84amount+F1FFQ85amount+F1FFQ86amount+F1FFQ63amount)
#OUTCOME
df <- df %>% dplyr::rename(MMSE_b = F1MME,
                          occ_b = F1job_curr8,
                          bmi_b = F1BMI,
                          sm_b = F1sbsmk,
                          depre_b = F1depressed,
                          totalcal_b = F1sumtot1,
                          F2totalcal = F2sumtot1,
                          famincome_b = F2income2, #many NAs
                          F1datquest = F1datblood,
                          diab_b = F1dbtld,
                          memory_b = F1_B16_REC,#max 48
                          verbal_b = F1_FV_A_TOT, 
                          stroop_b = F1_STROOP_IFORTE,#max 24
                          do40_b = F1_DO40, #max 40
                          CERAD_b = F1_CERAD_TOT, #max 11
                          F2memory = F2_B16_REC,#max 48
                          F2verbal = F2_FV_A_TOT, 
                          F2stroop = F2_STROOP_IFORTE,#max 24
                          F2do40 = F2_DO40, #max 40
                          F2CERAD = F2_CERAD_TOT, #max 11
                          F2CDR = F2_CDR,
                          F3memory = F3_B16_REC,#max 48
                          F3verbal = F3_FV_A_TOT, 
                          F3stroop = F3_STROOP_IFORTE,#max 24
                          F3do40 = F3_DO40, #max 40
                          F3CERAD = F3_CERAD_TOT)#max 11 
df[df == "."] <- NA

df <- df %>%
        mutate_at(c("F3_QPC_B","F3_QPC_A","F3_QPC_1","F3_QPC_2","F3_QPC_3","F3_QPC_4","F3_QPC_5",
                    'F3_QPC_8', 'F3_QPC_7',"F3_QPC_6","F2_QPC_B","F2_QPC_A","F2_QPC_1","F2_QPC_2","F2_QPC_3",
                    "F2_QPC_4","F2_QPC_5",'F2_QPC_8', 'F2_QPC_7',"F2_QPC_6","F1_QPC_B","F1_QPC_A","F1_QPC_1",
                    "F1_QPC_2","F1_QPC_3","F1_QPC_4","F1_QPC_5",'F1_QPC_8', 'F1_QPC_7',"F1_QPC_6","memory_b","verbal_b",
                    "stroop_b","do40_b","CERAD_b","F2memory","F2verbal","F2stroop", "F2do40", "F2CERAD", "F3memory",
                    "F3verbal","F3stroop", "F3do40", "F3CERAD"), as.numeric)
#COVARIATES

df$sm_b <- as.factor(df$sm_b)
df$occ_b <- as.factor(df$occ_b)
df$famincome_b <- as.factor(df$famincome_b)

df <- df %>% mutate(edu = ifelse(edtyp == 0 |edtyp == 1 | edtyp == 2, 1, #elementary
                                 ifelse(edtyp == 3, 2,                   #high sdfool
                                       ifelse(edtyp == 4, 3, NA))))          #superior

df$edu <- factor(df$edu,
levels = c(1,2,3),
labels = c("Elementary", "High school", "Superior"))

df$bmi_cat <- as.factor(ifelse(df$bmi_b < 25, "Normal",
                    ifelse(df$bmi_b < 30, "Overweight", 
                          ifelse(df$bmi_b >= 30, "Obese", NA))))

rep_str = c('jan'='-01-','feb'='-02-','mar'='-03-','apr'='-04-','may'='-05-','jun'='-06-','jul'='-07-','aug'='-08-',
            'sep'='-09-', 'oct'='-10-','nov'='-11-','dec'='-12-')
df$datbirth <- str_replace_all(df$datbirth, rep_str)
df$datexam <- str_replace_all(df$datexam, rep_str)
df$F1datquest <- str_replace_all(df$F1datquest, rep_str)

table(is.na(df$datbirth))

df$datbirth <- as.Date(df$datbirth,
  format = "%d-%m-%Y")
df$datexam <- as.Date(df$datexam,
  format = "%d-%m-%Y")
df$F1datquest <- as.Date(df$F1datquest,
  format = "%d-%m-%Y")

df$age_recruit <- difftime(df$datexam, df$datbirth, units = "days")
df$age_recruit <- as.integer(round(df$age_recruit/365,digits = 0))

df$age_b <- difftime(df$F1datquest, df$datbirth, units = "days")
df$age_b <- as.integer(round(df$age_b/365,digits = 0))

df <- df %>%
    dplyr::mutate(age_cat = as.factor(ifelse(age_b < 70, "Below 70",
                                  ifelse(age_b < 75, "Between 70 and 74",
                                        ifelse(age_b >= 75, "Over 74", NA)))))
df <- df %>% mutate(cvevent_b = ifelse(cmp == 1 | hdc == 1 |hdv == 1 | chf == 1 |cad == 1 | angn == 1 |miac == 1 |
                                       strk == 1 | ccth == 1 | cabg == 1 |pcin == 1|
                                       F1cmp == 1 | F1hdv == 1 | F1chf == 1 |F1cad == 1 | F1angn == 1 |F1miac == 1 |
                                       F1strk == 1 | F1ccth == 1 | F1cabg == 1 , 1, 0))

df <- df %>% mutate(HTA_b = ifelse(antiHTA == 1 | HTA == 1 | F1antiHTA == 1 | F1HTA == 1, 1, 0))


# Find tertiles
vTert = quantile(na.omit(df$F1etsem), c(0:3/3))

df <- df %>% mutate(pa_b = ifelse(is.na(F1etsem), NA, 
                                 ifelse(F1etsem < 16200.16, "Low", 
                                       ifelse(F1etsem < 19807.82, "Medium",
                                             ifelse(F1etsem < 44913.35, "High")))))

df <- df %>% mutate(alc_b = ifelse(alcool1 == 0, 0,
                                   ifelse(sex == 0 & F1alcool1 == 1, 1, 
                                      ifelse(sex == 0 & (F1alcool1 == 2 | F1alcool1 == 3),2,
                                        ifelse(sex == 0 & (F1alcool1 == 4 | F1alcool1 == 5 | alcool1 ==6),2,
                                            ifelse(sex == 1 & (F1alcool1 == 1|F1alcool1 ==2), 1, 
                                                ifelse(sex == 1 & (F1alcool1 == 3 | F1alcool1 == 4),2,
                                                    ifelse(sex == 1 & (F1alcool1 == 5 | F1alcool1 ==6),2, NA))))))))

df["F2datquest"][df["F2datquest"] == "03jul2004"] <- "03jul2014"

df$F2datquest <- str_replace_all(df$F2datquest, rep_str)
df$F3datquest <- str_replace_all(df$F3datquest, rep_str)

df$F2datquest <- as.Date(df$F2datquest,
  format = "%d-%m-%Y")
df$F3datquest <- as.Date(df$F3datquest,
  format = "%d-%m-%Y")

df$futime_b <- difftime(df$F1datquest, df$datexam, units = "days")
df$futime_b <- as.numeric(round(df$futime_b/365,digits = 2))

df$futime_F1 <- difftime(df$F2datquest, df$F1datquest, units = "days")
df$futime_F1 <- as.numeric(round(df$futime_F1/365,digits = 2))

df$futime_F2 <- difftime(df$F3datquest, df$F1datquest, units = "days")
df$futime_F2 <- as.numeric(round(df$futime_F2/365,digits = 2))
```

```
   0    1 
1272 4163
```

```
FALSE  TRUE 
 6733    17
```

#### 1C. Selection criteria¶

In [24]:

```
df <- df %>% #6733 at df
        filter(!is.na(MMSE_b)) %>% #n=1995
        filter((sex == 1 & totalcal_b > 799 & totalcal_b < 4001)|(sex == 0 & totalcal_b > 499 & totalcal_b < 3501))%>%
        filter(age_b > 55)%>% #<55 years old at baseline (n=0)
        filter(MMSE_b > 23)#baseline dietary assessment at least 3 years before the 3rd cognitive function assessment (N=306)
```

#### 1D. Table 1 - Baseline characteristics¶

In [25]:

```
df <- filter(df, !is.na(lp)) ## Vector of variables to summarize
baselinevars <- c("sex", "age_cat", "edu","bmi_cat", "sm_b","cvevent_b","HTA_b", "diab_b","pa_b", "MMSE_b","nonferm_dairy_b","total_dairy_b")

## Vector of categorical variables that need transformation
catvars <- c("sex", "age_cat", "edu","bmi_cat", "sm_b","cvevent_b","HTA_b", "diab_b","pa_b")

## Create the table object
tab1 <- CreateTableOne(vars = baselinevars, data = df, factorVars = catvars, test = FALSE)
tab1Mat <- print(tab1, smd = TRUE)
#tab1_csv <- write.csv(tab1Mat, file = "C:/Users/no22t395/OneDrive - Universitaet Bern/PhD project/Aim 4 - MR/CoLTable1_MR.csv")

tab1_str <- CreateTableOne(vars = baselinevars, strata = "lp", data = df, factorVars = catvars, test = FALSE)     
tab1Mat_str <- print(tab1_str, smd = TRUE)
#tab1_csv_str <- write.csv(tab1Mat_str, file = "C:/Users/no22t395/OneDrive - Universitaet Bern/PhD project/Aim 4 - MR/CoLTable1_MRstrat.csv")
```

```
                              Overall        
  n                             1565         
  sex = 1 (%)                    664 (42.4)  
  age_cat (%)                                
     Below 70                    948 (60.6)  
     Between 70 and 74           356 (22.7)  
     Over 74                     261 (16.7)  
  edu (%)                                    
     Elementary                 1081 (69.1)  
     High school                 251 (16.0)  
     Superior                    233 (14.9)  
  bmi_cat (%)                                
     Normal                      594 (38.4)  
     Obese                       311 (20.1)  
     Overweight                  641 (41.5)  
  sm_b (%)                                   
     0                           648 (41.5)  
     1                           683 (43.8)  
     2                           229 (14.7)  
  cvevent_b = 1 (%)              318 (20.4)  
  HTA_b = 1 (%)                 1056 (67.6)  
  diab_b = 1 (%)                 186 (11.9)  
  pa_b (%)                                   
     High                        268 (19.6)  
     Low                         623 (45.6)  
     Medium                      475 (34.8)  
  MMSE_b (mean (SD))           29.35 (1.15)  
  nonferm_dairy_b (mean (SD))  83.61 (110.57)
  total_dairy_b (mean (SD))   249.27 (188.55)
                             Stratified by lp
                              0               1               SMD   
  n                              327            1238                
  sex = 1 (%)                    157 (48.0)      507 (41.0)    0.142
  age_cat (%)                                                  0.041
     Below 70                    201 (61.5)      747 (60.3)         
     Between 70 and 74            70 (21.4)      286 (23.1)         
     Over 74                      56 (17.1)      205 (16.6)         
  edu (%)                                                      0.144
     Elementary                  210 (64.2)      871 (70.4)         
     High school                  56 (17.1)      195 (15.8)         
     Superior                     61 (18.7)      172 (13.9)         
  bmi_cat (%)                                                  0.111
     Normal                      115 (35.8)      479 (39.1)         
     Obese                        59 (18.4)      252 (20.6)         
     Overweight                  147 (45.8)      494 (40.3)         
  sm_b (%)                                                     0.064
     0                           143 (43.9)      505 (40.9)         
     1                           135 (41.4)      548 (44.4)         
     2                            48 (14.7)      181 (14.7)         
  cvevent_b = 1 (%)               76 (23.2)      242 (19.6)    0.089
  HTA_b = 1 (%)                  223 (68.2)      833 (67.4)    0.017
  diab_b = 1 (%)                  42 (12.8)      144 (11.6)    0.037
  pa_b (%)                                                     0.055
     High                         61 (21.3)      207 (19.2)         
     Low                         130 (45.3)      493 (45.7)         
     Medium                       96 (33.4)      379 (35.1)         
  MMSE_b (mean (SD))           29.31 (1.20)    29.35 (1.13)    0.039
  nonferm_dairy_b (mean (SD))  71.49 (98.82)   86.81 (113.29)  0.144
  total_dairy_b (mean (SD))   229.67 (165.18) 254.45 (193.99)  0.138
```

## Ratio Method¶

In [26]:

```
x <- ivreg(total_dairy_b ~ lp, data = df)
x
confint(x)
```

```
Call:
ivreg(formula = total_dairy_b ~ lp, data = df)

Coefficients:
(Intercept)           lp  
     229.67        24.78
```

A matrix: 2 × 2 of type dbl

|  | 2.5 % | 97.5 % |
| --- | --- | --- |
| (Intercept) | 209.238025 | 250.09706 |
| lp | 1.810276 | 47.74963 |

In [27]:

```
a <- lm(nonferm_dairy_b ~ lp, data = df)
summary(a)
confint(a)

o <- lm(nonferm_dairy_b ~ lp+sex+age_b+F1ht+bmi_b, data = df)
summary(o)
```

```
Call:
lm(formula = nonferm_dairy_b ~ lp, data = df)

Residuals:
   Min     1Q Median     3Q    Max 
-86.81 -65.38 -37.88  24.98 878.03 

Coefficients:
            Estimate Std. Error t value Pr(>|t|)    
(Intercept)   71.488      6.107  11.707   <2e-16 ***
lp            15.322      6.866   2.232   0.0258 *  
---
Signif. codes:  0 '***' 0.001 '**' 0.01 '*' 0.05 '.' 0.1 ' ' 1

Residual standard error: 110.4 on 1563 degrees of freedom
Multiple R-squared:  0.003176,	Adjusted R-squared:  0.002538 
F-statistic:  4.98 on 1 and 1563 DF,  p-value: 0.02579
```

A matrix: 2 × 2 of type dbl

|  | 2.5 % | 97.5 % |
| --- | --- | --- |
| (Intercept) | 59.51051 | 83.46644 |
| lp | 1.85435 | 28.78891 |

```
Call:
lm(formula = nonferm_dairy_b ~ lp + sex + age_b + F1ht + bmi_b, 
    data = df)

Residuals:
   Min     1Q Median     3Q    Max 
-97.32 -64.98 -38.98  22.92 887.19 

Coefficients:
            Estimate Std. Error t value Pr(>|t|)  
(Intercept)  38.7287    84.6634   0.457   0.6474  
lp           17.3233     6.9176   2.504   0.0124 *
sex           9.5428     7.8554   1.215   0.2246  
age_b         0.5997     0.5204   1.152   0.2493  
F1ht         -0.1108     0.4216  -0.263   0.7926  
bmi_b         0.1745     0.6202   0.281   0.7785  
---
Signif. codes:  0 '***' 0.001 '**' 0.01 '*' 0.05 '.' 0.1 ' ' 1

Residual standard error: 110 on 1540 degrees of freedom
  (19 Beobachtungen als fehlend gelöscht)
Multiple R-squared:  0.006311,	Adjusted R-squared:  0.003085 
F-statistic: 1.956 on 5 and 1540 DF,  p-value: 0.08233
```

In [28]:

```
#Crude estimates - request Reviewer 1

summary(lm(F2MME ~ lp, data = df))
confint(lm(F2MME ~ lp, data = df))
summary(lm(F2memory ~ lp, data = df))
confint(lm(F2memory ~ lp, data = df))
summary(lm(F2verbal ~ lp, data = df))
confint(lm(F2verbal ~ lp, data = df))
summary(lm(F2stroop ~ lp, data = df))
confint(lm(F2stroop ~ lp, data = df))

w <- lm(F2MME ~ nonferm_dairy_b, data = df)
c(w$coefficients[2]*50, confint(w)[2,1]*50, confint(w)[2,2]*50)
z <- lm(F2memory ~ nonferm_dairy_b, data = df)
c(z$coefficients[2]*50, confint(z)[2,1]*50, confint(z)[2,2]*50)
y <- lm(F2verbal ~ nonferm_dairy_b, data = df)
c(y$coefficients[2]*50, confint(y)[2,1]*50, confint(y)[2,2]*50)
v <- lm(F2stroop ~ nonferm_dairy_b, data = df)
c(v$coefficients[2]*50, confint(v)[2,1]*50, confint(v)[2,2]*50)

w <- lm(F2MME ~ total_dairy_b, data = df)
c(w$coefficients[2]*50, confint(w)[2,1]*50, confint(w)[2,2]*50)
z <- lm(F2memory ~ total_dairy_b, data = df)
c(z$coefficients[2]*50, confint(z)[2,1]*50, confint(z)[2,2]*50)
y <- lm(F2verbal ~ total_dairy_b, data = df)
c(y$coefficients[2]*50, confint(y)[2,1]*50, confint(y)[2,2]*50)
v <- lm(F2stroop ~ total_dairy_b, data = df)
c(v$coefficients[2]*50, confint(v)[2,1]*50, confint(v)[2,2]*50)
```

```
Call:
lm(formula = F2MME ~ lp, data = df)

Residuals:
     Min       1Q   Median       3Q      Max 
-28.9748  -0.0161   0.9839   1.0252   1.0252 

Coefficients:
            Estimate Std. Error t value Pr(>|t|)    
(Intercept) 29.01613    0.12956 223.951   <2e-16 ***
lp          -0.04137    0.14548  -0.284    0.776    
---
Signif. codes:  0 '***' 0.001 '**' 0.01 '*' 0.05 '.' 0.1 ' ' 1

Residual standard error: 2.04 on 1197 degrees of freedom
  (366 Beobachtungen als fehlend gelöscht)
Multiple R-squared:  6.754e-05,	Adjusted R-squared:  -0.0007678 
F-statistic: 0.08085 on 1 and 1197 DF,  p-value: 0.7762
```

A matrix: 2 × 2 of type dbl

|  | 2.5 % | 97.5 % |
| --- | --- | --- |
| (Intercept) | 28.7619297 | 29.2703283 |
| lp | -0.3267917 | 0.2440605 |

```
Call:
lm(formula = F2memory ~ lp, data = df)

Residuals:
     Min       1Q   Median       3Q      Max 
-31.5719   0.8239   1.4281   1.4281   1.4281 

Coefficients:
            Estimate Std. Error t value Pr(>|t|)    
(Intercept)  47.1761     0.4249 111.025   <2e-16 ***
lp           -0.6043     0.4771  -1.267    0.206    
---
Signif. codes:  0 '***' 0.001 '**' 0.01 '*' 0.05 '.' 0.1 ' ' 1

Residual standard error: 5.637 on 849 degrees of freedom
  (714 Beobachtungen als fehlend gelöscht)
Multiple R-squared:  0.001886,	Adjusted R-squared:  0.0007103 
F-statistic: 1.604 on 1 and 849 DF,  p-value: 0.2057
```

A matrix: 2 × 2 of type dbl

|  | 2.5 % | 97.5 % |
| --- | --- | --- |
| (Intercept) | 46.342131 | 48.0101417 |
| lp | -1.540728 | 0.3321593 |

```
Call:
lm(formula = F2verbal ~ lp, data = df)

Residuals:
     Min       1Q   Median       3Q      Max 
-27.1283  -6.1283  -0.1283   5.8717  27.8717 

Coefficients:
            Estimate Std. Error t value Pr(>|t|)    
(Intercept) 30.15517    0.64769  46.558   <2e-16 ***
lp          -0.02685    0.72606  -0.037    0.971    
---
Signif. codes:  0 '***' 0.001 '**' 0.01 '*' 0.05 '.' 0.1 ' ' 1

Residual standard error: 8.544 on 850 degrees of freedom
  (713 Beobachtungen als fehlend gelöscht)
Multiple R-squared:  1.609e-06,	Adjusted R-squared:  -0.001175 
F-statistic: 0.001368 on 1 and 850 DF,  p-value: 0.9705
```

A matrix: 2 × 2 of type dbl

|  | 2.5 % | 97.5 % |
| --- | --- | --- |
| (Intercept) | 28.883911 | 31.426434 |
| lp | -1.451936 | 1.398228 |

```
Call:
lm(formula = F2stroop ~ lp, data = df)

Residuals:
     Min       1Q   Median       3Q      Max 
-20.2361  -0.2361   0.7639   0.7639   0.9209 

Coefficients:
            Estimate Std. Error t value Pr(>|t|)    
(Intercept)  23.0791     0.1361 169.571   <2e-16 ***
lp            0.1570     0.1527   1.028    0.304    
---
Signif. codes:  0 '***' 0.001 '**' 0.01 '*' 0.05 '.' 0.1 ' ' 1

Residual standard error: 1.811 on 857 degrees of freedom
  (706 Beobachtungen als fehlend gelöscht)
Multiple R-squared:  0.001231,	Adjusted R-squared:  6.541e-05 
F-statistic: 1.056 on 1 and 857 DF,  p-value: 0.3044
```

A matrix: 2 × 2 of type dbl

|  | 2.5 % | 97.5 % |
| --- | --- | --- |
| (Intercept) | 22.811962 | 23.3462300 |
| lp | -0.142827 | 0.4567756 |

nonferm\_dairy\_b
:   0.00546729481655511

2
:   -0.0459730166643223

3
:   0.0569076062974325

nonferm\_dairy\_b
:   -0.0127625615187618

2
:   -0.17776312524957

3
:   0.152238002212046

nonferm\_dairy\_b
:   -0.0768097533174921

2
:   -0.326734873569626

3
:   0.173115366934642

nonferm\_dairy\_b
:   0.0199319994731775

2
:   -0.0337133975780892

3
:   0.0735773965244441

total\_dairy\_b
:   0.0281632220993733

2
:   -0.00338429419596107

3
:   0.0597107383947077

total\_dairy\_b
:   -0.0556619000468695

2
:   -0.156718389622333

3
:   0.0453945895285943

total\_dairy\_b
:   -0.0673282164141844

2
:   -0.220046059121121

3
:   0.0853896262927518

total\_dairy\_b
:   -0.00728359598092273

2
:   -0.0400021581273967

3
:   0.0254349661655513

In [29]:

```
a<- lm(F2MME ~ rs4988235+sex+age_b+F1ht+bmi_b, data = df)
b<- lm(F2CERAD ~ rs4988235+sex+age_b+F1ht+bmi_b, data = df)
c<- lm(F2do40 ~ rs4988235+sex+age_b+F1ht+bmi_b, data = df)
d<- lm(F2memory ~ rs4988235+sex+age_b+F1ht+bmi_b, data = df)
e<- lm(F2verbal ~ rs4988235+sex+age_b+F1ht+bmi_b, data = df)
f<- lm(F2stroop ~ rs4988235+sex+age_b+F1ht+bmi_b, data = df)

g<- lm(F3MME ~ rs4988235+sex+age_b+F1ht+bmi_b, data = df)
h<- lm(F3CERAD ~ rs4988235+sex+age_b+F1ht+bmi_b, data = df)
i<- lm(F3do40 ~ rs4988235+sex+age_b+F1ht+bmi_b, data = df)
j<- lm(F3memory ~ rs4988235+sex+age_b+F1ht+bmi_b, data = df)
k<- lm(F3verbal ~ rs4988235+sex+age_b+F1ht+bmi_b, data = df)
l<- lm(F3stroop ~ rs4988235+sex+age_b+F1ht+bmi_b, data = df)
```

## 2SLS - Package (correct SE) with BMI¶

In [30]:

```
a2<- ivreg(F2MME ~ sex+age_b+F1ht+bmi_b | nonferm_dairy_b | rs4988235 + sex+age_b+F1ht+bmi_b, data = df)
b2<- ivreg(F2CERAD ~ sex+age_b+F1ht+bmi_b | nonferm_dairy_b | rs4988235 + sex+age_b+F1ht+bmi_b, data = df)
c2<- ivreg(F2do40 ~ sex+age_b+F1ht+bmi_b | nonferm_dairy_b | rs4988235 + sex+age_b+F1ht+bmi_b, data = df)
d2<- ivreg(F2memory ~ sex+age_b+F1ht+bmi_b | nonferm_dairy_b | rs4988235 + sex+age_b+F1ht+bmi_b, data = df)
e2<- ivreg(F2verbal ~ sex+age_b+F1ht+bmi_b | nonferm_dairy_b | rs4988235 + sex+age_b+F1ht+bmi_b, data = df)
f2<- ivreg(F2stroop ~ sex+age_b+F1ht+bmi_b | nonferm_dairy_b | rs4988235 + sex+age_b+F1ht+bmi_b, data = df)

g2<- ivreg(F3MME ~ sex+age_b+F1ht+bmi_b | nonferm_dairy_b | rs4988235 + sex+age_b+F1ht+bmi_b, data = df)
h2<- ivreg(F3CERAD ~ sex+age_b+F1ht+bmi_b | nonferm_dairy_b | rs4988235 + sex+age_b+F1ht+bmi_b, data = df)
i2<- ivreg(F3do40 ~ sex+age_b+F1ht+bmi_b | nonferm_dairy_b | rs4988235 + sex+age_b+F1ht+bmi_b, data = df)
j2<- ivreg(F3memory ~ sex+age_b+F1ht+bmi_b | nonferm_dairy_b | rs4988235 + sex+age_b+F1ht+bmi_b, data = df)
k2<- ivreg(F3verbal ~ sex+age_b+F1ht+bmi_b | nonferm_dairy_b | rs4988235 + sex+age_b+F1ht+bmi_b, data = df)
l2<- ivreg(F3stroop ~ sex+age_b+F1ht+bmi_b | nonferm_dairy_b | rs4988235 + sex+age_b+F1ht+bmi_b, data = df)
```

In [31]:

```
table = data.frame(tp = c("F2","F2","F2","F2","F2","F2", "F3","F3","F3","F3","F3","F3"),
                   test = c("MMSE", "CERAD", "DO40", "MEMORY", "VERBAL", "STROOP",
                            "MMSE", "CERAD", "DO40", "MEMORY", "VERBAL", "STROOP"),
                   estimate_ratio = c((a$coefficients[2]*50)/o$coefficients[2],(b$coefficients[2]*50)/o$coefficients[2],
                                       (c$coefficients[2]*50)/o$coefficients[2],(d$coefficients[2]*50)/o$coefficients[2],
                                       (e$coefficients[2]*50)/o$coefficients[2],(f$coefficients[2]*50)/o$coefficients[2],
                                      (g$coefficients[2]*50)/o$coefficients[2],(h$coefficients[2]*50)/o$coefficients[2],
                                       (i$coefficients[2]*50)/o$coefficients[2],(j$coefficients[2]*50)/o$coefficients[2],
                                       (k$coefficients[2]*50)/o$coefficients[2],(l$coefficients[2]*50)/o$coefficients[2]),
                  estimate_2sls = c(a2$coefficients[2]*50,b2$coefficients[2]*50,c2$coefficients[2]*50,d2$coefficients[2]*50,
                                    e2$coefficients[2]*50,f2$coefficients[2]*50,g2$coefficients[2]*50,h2$coefficients[2]*50,
                                    i2$coefficients[2]*50,j2$coefficients[2]*50,k2$coefficients[2]*50,l2$coefficients[2]*50),
                  lb_2sls = c(confint(a2)[2,1]*50,confint(b2)[2,1]*50,confint(c2)[2,1]*50,confint(d2)[2,1]*50,confint(e2)[2,1]*50,
                             confint(f2)[2,1]*50,confint(g2)[2,1]*50,confint(h2)[2,1]*50,confint(i2)[2,1]*50,confint(j2)[2,1]*50,
                             confint(k2)[2,1]*50,confint(l2)[2,1]*50),
                   ub_2sls = c(confint(a2)[2,2]*50,confint(b2)[2,2]*50,confint(c2)[2,2]*50,confint(d2)[2,2]*50,confint(e2)[2,2]*50,
                             confint(f2)[2,2]*50,confint(g2)[2,2]*50,confint(h2)[2,2]*50,confint(i2)[2,2]*50,confint(j2)[2,2]*50,
                             confint(k2)[2,2]*50,confint(l2)[2,2]*50))
table
```

A data.frame: 12 × 6

| tp | test | estimate\_ratio | estimate\_2sls | lb\_2sls | ub\_2sls |
| --- | --- | --- | --- | --- | --- |
| <chr> | <chr> | <dbl> | <dbl> | <dbl> | <dbl> |
| F2 | MMSE | 0.11716104 | 0.1748270 | -0.5982301 | 0.9478841 |
| F2 | CERAD | 0.10447212 | 0.1462655 | -0.4186436 | 0.7111746 |
| F2 | DO40 | 0.22691722 | 0.3281473 | -0.2779636 | 0.9342582 |
| F2 | MEMORY | -0.66455746 | -0.9662905 | -3.5944817 | 1.6619007 |
| F2 | VERBAL | 0.66956544 | 0.9452429 | -2.6608338 | 4.5513197 |
| F2 | STROOP | 0.32727208 | 0.4903118 | -0.4174138 | 1.3980375 |
| F3 | MMSE | -0.47828281 | -0.6067865 | -1.5002032 | 0.2866303 |
| F3 | CERAD | -0.07421591 | -0.2269102 | -1.9730371 | 1.5192166 |
| F3 | DO40 | 0.32113227 | 1.2420459 | -3.8244327 | 6.3085245 |
| F3 | MEMORY | 0.14434266 | 0.5748954 | -3.0916260 | 4.2414169 |
| F3 | VERBAL | -1.42896896 | -5.2232413 | -29.1354048 | 18.6889222 |
| F3 | STROOP | 0.03487967 | 0.1162497 | -2.2272477 | 2.4597471 |

In [32]:

```
a2<- ivreg(F2MME ~ sex+age_b+F1ht+bmi_b | total_dairy_b | rs4988235 + sex+age_b+F1ht+bmi_b, data = df)
b2<- ivreg(F2CERAD ~ sex+age_b+F1ht+bmi_b | total_dairy_b | rs4988235 + sex+age_b+F1ht+bmi_b, data = df)
c2<- ivreg(F2do40 ~ sex+age_b+F1ht+bmi_b | total_dairy_b | rs4988235 + sex+age_b+F1ht+bmi_b, data = df)
d2<- ivreg(F2memory ~ sex+age_b+F1ht+bmi_b | total_dairy_b | rs4988235 + sex+age_b+F1ht+bmi_b, data = df)
e2<- ivreg(F2verbal ~ sex+age_b+F1ht+bmi_b | total_dairy_b | rs4988235 + sex+age_b+F1ht+bmi_b, data = df)
f2<- ivreg(F2stroop ~ sex+age_b+F1ht+bmi_b | total_dairy_b | rs4988235 + sex+age_b+F1ht+bmi_b, data = df)

g2<- ivreg(F3MME ~ sex+age_b+F1ht+bmi_b | total_dairy_b | rs4988235 + sex+age_b+F1ht+bmi_b, data = df)
h2<- ivreg(F3CERAD ~ sex+age_b+F1ht+bmi_b | total_dairy_b | rs4988235 + sex+age_b+F1ht+bmi_b, data = df)
i2<- ivreg(F3do40 ~ sex+age_b+F1ht+bmi_b | total_dairy_b | rs4988235 + sex+age_b+F1ht+bmi_b, data = df)
j2<- ivreg(F3memory ~ sex+age_b+F1ht+bmi_b | total_dairy_b | rs4988235 + sex+age_b+F1ht+bmi_b, data = df)
k2<- ivreg(F3verbal ~ sex+age_b+F1ht+bmi_b | total_dairy_b | rs4988235 + sex+age_b+F1ht+bmi_b, data = df)
l2<- ivreg(F3stroop ~ sex+age_b+F1ht+bmi_b | total_dairy_b | rs4988235 + sex+age_b+F1ht+bmi_b, data = df)

summary(a2)
```

```
Call:
ivreg(formula = F2MME ~ sex + age_b + F1ht + bmi_b | total_dairy_b | 
    rs4988235 + sex + age_b + F1ht + bmi_b, data = df)

Residuals:
     Min       1Q   Median       3Q      Max 
-29.0487  -0.5700   0.4116   1.0664   2.3583 

Coefficients:
               Estimate Std. Error t value Pr(>|t|)    
(Intercept)   25.734356   1.880445  13.685  < 2e-16 ***
total_dairy_b  0.003687   0.008502   0.434  0.66464    
sex           -0.634138   0.186813  -3.395  0.00071 ***
age_b         -0.032289   0.011916  -2.710  0.00683 ** 
F1ht           0.028264   0.019474   1.451  0.14693    
bmi_b          0.002538   0.022110   0.115  0.90862    

Diagnostic tests:
                  df1  df2 statistic p-value
Weak instruments    1 1180     1.842   0.175
Wu-Hausman          1 1179     0.155   0.694
Sargan              0   NA        NA      NA

Residual standard error: 2.103 on 1180 degrees of freedom
Multiple R-Squared: -0.05318,	Adjusted R-squared: -0.05764 
Wald test: 6.073 on 5 and 1180 DF,  p-value: 1.462e-05
```

In [33]:

```
data.frame(tp = c("F2 tot","F2 tot","F2 tot","F2 tot","F2 tot","F2 tot","F3 tot","F3 tot","F3 tot","F3 tot","F3 tot","F3 tot"),
           test = c("MMSE", "CERAD", "DO40", "MEMORY", "VERBAL", "STROOP",
                    "MMSE", "CERAD", "DO40", "MEMORY", "VERBAL", "STROOP"),
           estimate_ratio = c((a$coefficients[2]*50)/o$coefficients[2],(b$coefficients[2]*50)/o$coefficients[2],
                               (c$coefficients[2]*50)/o$coefficients[2],(d$coefficients[2]*50)/o$coefficients[2],
                               (e$coefficients[2]*50)/o$coefficients[2],(f$coefficients[2]*50)/o$coefficients[2],
                              (g$coefficients[2]*50)/o$coefficients[2],(h$coefficients[2]*50)/o$coefficients[2],
                               (i$coefficients[2]*50)/o$coefficients[2],(j$coefficients[2]*50)/o$coefficients[2],
                               (k$coefficients[2]*50)/o$coefficients[2],(l$coefficients[2]*50)/o$coefficients[2]),
          estimate_2sls = c(a2$coefficients[2]*50,b2$coefficients[2]*50,c2$coefficients[2]*50,d2$coefficients[2]*50,
                            e2$coefficients[2]*50,f2$coefficients[2]*50,g2$coefficients[2]*50,h2$coefficients[2]*50,
                            i2$coefficients[2]*50,j2$coefficients[2]*50,k2$coefficients[2]*50,l2$coefficients[2]*50),
          lb_2sls = c(confint(a2)[2,1]*50,confint(b2)[2,1]*50,confint(c2)[2,1]*50,confint(d2)[2,1]*50,confint(e2)[2,1]*50,
                     confint(f2)[2,1]*50,confint(g2)[2,1]*50,confint(h2)[2,1]*50,confint(i2)[2,1]*50,confint(j2)[2,1]*50,
                     confint(k2)[2,1]*50,confint(l2)[2,1]*50),
           ub_2sls = c(confint(a2)[2,2]*50,confint(b2)[2,2]*50,confint(c2)[2,2]*50,confint(d2)[2,2]*50,confint(e2)[2,2]*50,
                     confint(f2)[2,2]*50,confint(g2)[2,2]*50,confint(h2)[2,2]*50,confint(i2)[2,2]*50,confint(j2)[2,2]*50,
                     confint(k2)[2,2]*50,confint(l2)[2,2]*50))
```

A data.frame: 12 × 6

| tp | test | estimate\_ratio | estimate\_2sls | lb\_2sls | ub\_2sls |
| --- | --- | --- | --- | --- | --- |
| <chr> | <chr> | <dbl> | <dbl> | <dbl> | <dbl> |
| F2 tot | MMSE | 0.11716104 | 0.1843261 | -6.496818e-01 | 1.018334e+00 |
| F2 tot | CERAD | 0.10447212 | 0.1947315 | -6.558878e-01 | 1.045351e+00 |
| F2 tot | DO40 | 0.22691722 | 0.4992115 | -9.562494e-01 | 1.954672e+00 |
| F2 tot | MEMORY | -0.66455746 | -1.5962460 | -7.368989e+00 | 4.176497e+00 |
| F2 tot | VERBAL | 0.66956544 | 1.6458669 | -6.073695e+00 | 9.365429e+00 |
| F2 tot | STROOP | 0.32727208 | 0.8778324 | -2.099978e+00 | 3.855642e+00 |
| F3 tot | MMSE | -0.47828281 | -0.6597668 | -1.909616e+00 | 5.900820e-01 |
| F3 tot | CERAD | -0.07421591 | -0.5526354 | -8.447213e+00 | 7.341942e+00 |
| F3 tot | DO40 | 0.32113227 | -66.4929369 | -2.259008e+04 | 2.245709e+04 |
| F3 tot | MEMORY | 0.14434266 | -2.6303414 | -8.241360e+01 | 7.715291e+01 |
| F3 tot | VERBAL | -1.42896896 | -628.9077839 | -4.558243e+05 | 4.545665e+05 |
| F3 tot | STROOP | 0.03487967 | 0.7961298 | -3.384318e+01 | 3.543544e+01 |

## 2SLS - Package (correct SE) without BMI for sensitivity analysis¶

In [34]:

```
a2<- ivreg(F2MME ~ nonferm_dairy_b+sex+age_b+F1ht | rs4988235 + sex+age_b+F1ht, data = df)
b2<- ivreg(F2CERAD ~ sex+age_b+F1ht | nonferm_dairy_b | rs4988235 + sex+age_b+F1ht, data = df)
c2<- ivreg(F2do40 ~ sex+age_b+F1ht | nonferm_dairy_b | rs4988235 + sex+age_b+F1ht, data = df)
d2<- ivreg(F2memory ~ sex+age_b+F1ht | nonferm_dairy_b | rs4988235 + sex+age_b+F1ht, data = df)
e2<- ivreg(F2verbal ~ sex+age_b+F1ht | nonferm_dairy_b | rs4988235 + sex+age_b+F1ht, data = df)
f2<- ivreg(F2stroop ~ sex+age_b+F1ht | nonferm_dairy_b | rs4988235 + sex+age_b+F1ht, data = df)

g2<- ivreg(F3MME ~ sex+age_b+F1ht | nonferm_dairy_b | rs4988235 + sex+age_b+F1ht, data = df)
h2<- ivreg(F3CERAD ~ sex+age_b+F1ht | nonferm_dairy_b | rs4988235 + sex+age_b+F1ht, data = df)
i2<- ivreg(F3do40 ~ sex+age_b+F1ht | nonferm_dairy_b | rs4988235 + sex+age_b+F1ht, data = df)
j2<- ivreg(F3memory ~ sex+age_b+F1ht | nonferm_dairy_b | rs4988235 + sex+age_b+F1ht, data = df)
k2<- ivreg(F3verbal ~ sex+age_b+F1ht | nonferm_dairy_b | rs4988235 + sex+age_b+F1ht, data = df)
l2<- ivreg(F3stroop ~ sex+age_b+F1ht | nonferm_dairy_b | rs4988235 + sex+age_b+F1ht, data = df)
```

In [35]:

```
data.frame(tp = c("F2","F2","F2","F2","F2","F2", "F3","F3","F3","F3","F3","F3"),
           test = c("MMSE", "CERAD", "DO40", "MEMORY", "VERBAL", "STROOP",
                    "MMSE", "CERAD", "DO40", "MEMORY", "VERBAL", "STROOP"),
          estimate_2sls = c(a2$coefficients[2]*50,b2$coefficients[2]*50,c2$coefficients[2]*50,d2$coefficients[2]*50,
                            e2$coefficients[2]*50,f2$coefficients[2]*50,g2$coefficients[2]*50,h2$coefficients[2]*50,
                            i2$coefficients[2]*50,j2$coefficients[2]*50,k2$coefficients[2]*50,l2$coefficients[2]*50),
          lb_2sls = c(confint(a2)[2,1]*50,confint(b2)[2,1]*50,confint(c2)[2,1]*50,confint(d2)[2,1]*50,confint(e2)[2,1]*50,
                     confint(f2)[2,1]*50,confint(g2)[2,1]*50,confint(h2)[2,1]*50,confint(i2)[2,1]*50,confint(j2)[2,1]*50,
                     confint(k2)[2,1]*50,confint(l2)[2,1]*50),
           ub_2sls = c(confint(a2)[2,2]*50,confint(b2)[2,2]*50,confint(c2)[2,2]*50,confint(d2)[2,2]*50,confint(e2)[2,2]*50,
                     confint(f2)[2,2]*50,confint(g2)[2,2]*50,confint(h2)[2,2]*50,confint(i2)[2,2]*50,confint(j2)[2,2]*50,
                     confint(k2)[2,2]*50,confint(l2)[2,2]*50))
```

A data.frame: 12 × 5

| tp | test | estimate\_2sls | lb\_2sls | ub\_2sls |
| --- | --- | --- | --- | --- |
| <chr> | <chr> | <dbl> | <dbl> | <dbl> |
| F2 | MMSE | 0.1613855 | -0.5994308 | 0.9222019 |
| F2 | CERAD | 0.1088326 | -0.4497891 | 0.6674542 |
| F2 | DO40 | 0.3286039 | -0.2759799 | 0.9331878 |
| F2 | MEMORY | -1.0079267 | -3.6425711 | 1.6267178 |
| F2 | VERBAL | 0.6676421 | -2.8923696 | 4.2276538 |
| F2 | STROOP | 0.4422094 | -0.4385090 | 1.3229279 |
| F3 | MMSE | -0.6016960 | -1.4900517 | 0.2866597 |
| F3 | CERAD | -0.1419359 | -1.6524873 | 1.3686155 |
| F3 | DO40 | 1.1071713 | -3.0023292 | 5.2166719 |
| F3 | MEMORY | 0.5090306 | -2.6742797 | 3.6923409 |
| F3 | VERBAL | -5.2184039 | -26.8643661 | 16.4275583 |
| F3 | STROOP | 0.1426125 | -1.9427777 | 2.2280027 |

In [36]:

```
a2<- ivreg(F2MME ~ total_dairy_b+sex+age_b+F1ht | rs4988235 + sex+age_b+F1ht, data = df)
b2<- ivreg(F2CERAD ~ sex+age_b+F1ht | total_dairy_b | rs4988235 + sex+age_b+F1ht, data = df)
c2<- ivreg(F2do40 ~ sex+age_b+F1ht | total_dairy_b | rs4988235 + sex+age_b+F1ht, data = df)
d2<- ivreg(F2memory ~ sex+age_b+F1ht | total_dairy_b | rs4988235 + sex+age_b+F1ht, data = df)
e2<- ivreg(F2verbal ~ sex+age_b+F1ht | total_dairy_b | rs4988235 + sex+age_b+F1ht, data = df)
f2<- ivreg(F2stroop ~ sex+age_b+F1ht | total_dairy_b | rs4988235 + sex+age_b+F1ht, data = df)

g2<- ivreg(F3MME ~ sex+age_b+F1ht | total_dairy_b | rs4988235 + sex+age_b+F1ht, data = df)
h2<- ivreg(F3CERAD ~ sex+age_b+F1ht | total_dairy_b | rs4988235 + sex+age_b+F1ht, data = df)
i2<- ivreg(F3do40 ~ sex+age_b+F1ht | total_dairy_b | rs4988235 + sex+age_b+F1ht, data = df)
j2<- ivreg(F3memory ~ sex+age_b+F1ht | total_dairy_b | rs4988235 + sex+age_b+F1ht, data = df)
k2<- ivreg(F3verbal ~ sex+age_b+F1ht | total_dairy_b | rs4988235 + sex+age_b+F1ht, data = df)
l2<- ivreg(F3stroop ~ sex+age_b+F1ht | total_dairy_b | rs4988235 + sex+age_b+F1ht, data = df)

data.frame(tp = c("F2 tot","F2 tot","F2 tot","F2 tot","F2 tot","F2 tot","F3 tot","F3 tot","F3 tot","F3 tot","F3 tot","F3 tot"),
          test = c("MMSE", "CERAD", "DO40", "MEMORY", "VERBAL", "STROOP","MMSE", "CERAD", "DO40", "MEMORY", "VERBAL", "STROOP"),
          estimate_2sls = c(a2$coefficients[2]*50,b2$coefficients[2]*50,c2$coefficients[2]*50,d2$coefficients[2]*50,
                            e2$coefficients[2]*50,f2$coefficients[2]*50,g2$coefficients[2]*50,h2$coefficients[2]*50,
                            i2$coefficients[2]*50,j2$coefficients[2]*50,k2$coefficients[2]*50,l2$coefficients[2]*50),
          lb_2sls = c(confint(a2)[2,1]*50,confint(b2)[2,1]*50,confint(c2)[2,1]*50,confint(d2)[2,1]*50,confint(e2)[2,1]*50,
                     confint(f2)[2,1]*50,confint(g2)[2,1]*50,confint(h2)[2,1]*50,confint(i2)[2,1]*50,confint(j2)[2,1]*50,
                     confint(k2)[2,1]*50,confint(l2)[2,1]*50),
          ub_2sls = c(confint(a2)[2,2]*50,confint(b2)[2,2]*50,confint(c2)[2,2]*50,confint(d2)[2,2]*50,confint(e2)[2,2]*50,
                     confint(f2)[2,2]*50,confint(g2)[2,2]*50,confint(h2)[2,2]*50,confint(i2)[2,2]*50,confint(j2)[2,2]*50,
                     confint(k2)[2,2]*50,confint(l2)[2,2]*50))
```

A data.frame: 12 × 5

| tp | test | estimate\_2sls | lb\_2sls | ub\_2sls |
| --- | --- | --- | --- | --- |
| <chr> | <chr> | <dbl> | <dbl> | <dbl> |
| F2 tot | MMSE | 0.1699908 | -0.6466221 | 0.9866036 |
| F2 tot | CERAD | 0.1451720 | -0.6597748 | 0.9501189 |
| F2 tot | DO40 | 0.4991879 | -0.9508152 | 1.9491910 |
| F2 tot | MEMORY | -1.6563074 | -7.4985444 | 4.1859295 |
| F2 tot | VERBAL | 1.1690481 | -5.8671371 | 8.2052333 |
| F2 tot | STROOP | 0.7837650 | -1.9012009 | 3.4687308 |
| F3 tot | MMSE | -0.6620959 | -1.9270134 | 0.6028215 |
| F3 tot | CERAD | -0.2700700 | -3.8798206 | 3.3396805 |
| F3 tot | DO40 | 7.2192617 | -261.4350753 | 275.8735987 |
| F3 tot | MEMORY | -28.6247082 | -9448.9897458 | 9391.7403293 |
| F3 tot | VERBAL | -30.4906850 | -1001.8975127 | 940.9161426 |
| F3 tot | STROOP | 0.4666175 | -9.5937718 | 10.5270068 |

## 2SLS with BMI and no butter for sensitivity analysis¶

In [37]:

```
a2<- ivreg(F2MME ~ sex+age_b+F1ht+bmi_b | nobutterferm_dairy_b | rs4988235 + sex+age_b+F1ht+bmi_b, data = df)
b2<- ivreg(F2CERAD ~ sex+age_b+F1ht+bmi_b | nobutterferm_dairy_b | rs4988235 + sex+age_b+F1ht+bmi_b, data = df)
c2<- ivreg(F2do40 ~ sex+age_b+F1ht+bmi_b | nobutterferm_dairy_b | rs4988235 + sex+age_b+F1ht+bmi_b, data = df)
d2<- ivreg(F2memory ~ sex+age_b+F1ht+bmi_b | nobutterferm_dairy_b | rs4988235 + sex+age_b+F1ht+bmi_b, data = df)
e2<- ivreg(F2verbal ~ sex+age_b+F1ht+bmi_b | nobutterferm_dairy_b | rs4988235 + sex+age_b+F1ht+bmi_b, data = df)
f2<- ivreg(F2stroop ~ sex+age_b+F1ht+bmi_b | nobutterferm_dairy_b | rs4988235 + sex+age_b+F1ht+bmi_b, data = df)

g2<- ivreg(F3MME ~ sex+age_b+F1ht+bmi_b | nobutterferm_dairy_b | rs4988235 + sex+age_b+F1ht+bmi_b, data = df)
h2<- ivreg(F3CERAD ~ sex+age_b+F1ht+bmi_b | nobutterferm_dairy_b | rs4988235 + sex+age_b+F1ht+bmi_b, data = df)
i2<- ivreg(F3do40 ~ sex+age_b+F1ht+bmi_b | nobutterferm_dairy_b | rs4988235 + sex+age_b+F1ht+bmi_b, data = df)
j2<- ivreg(F3memory ~ sex+age_b+F1ht+bmi_b | nobutterferm_dairy_b | rs4988235 + sex+age_b+F1ht+bmi_b, data = df)
k2<- ivreg(F3verbal ~ sex+age_b+F1ht+bmi_b | nobutterferm_dairy_b | rs4988235 + sex+age_b+F1ht+bmi_b, data = df)
l2<- ivreg(F3stroop ~ sex+age_b+F1ht+bmi_b | nobutterferm_dairy_b | rs4988235 + sex+age_b+F1ht+bmi_b, data = df)
```

In [38]:

```
data.frame(tp = c("F2","F2","F2","F2","F2","F2", "F3","F3","F3","F3","F3","F3"),
          test = c("MMSE","CERAD","DO40","MEMORY","VERBAL","STROOP","MMSE","CERAD","DO40","MEMORY","VERBAL","STROOP"),
          estimate_2sls = c(a2$coefficients[2]*50,b2$coefficients[2]*50,c2$coefficients[2]*50,d2$coefficients[2]*50,
                            e2$coefficients[2]*50,f2$coefficients[2]*50,g2$coefficients[2]*50,h2$coefficients[2]*50,
                            i2$coefficients[2]*50,j2$coefficients[2]*50,k2$coefficients[2]*50,l2$coefficients[2]*50),
          lb_2sls = c(confint(a2)[2,1]*50,confint(b2)[2,1]*50,confint(c2)[2,1]*50,confint(d2)[2,1]*50,confint(e2)[2,1]*50,
                     confint(f2)[2,1]*50,confint(g2)[2,1]*50,confint(h2)[2,1]*50,confint(i2)[2,1]*50,confint(j2)[2,1]*50,
                     confint(k2)[2,1]*50,confint(l2)[2,1]*50),
          ub_2sls = c(confint(a2)[2,2]*50,confint(b2)[2,2]*50,confint(c2)[2,2]*50,confint(d2)[2,2]*50,confint(e2)[2,2]*50,
                     confint(f2)[2,2]*50,confint(g2)[2,2]*50,confint(h2)[2,2]*50,confint(i2)[2,2]*50,confint(j2)[2,2]*50,
                     confint(k2)[2,2]*50,confint(l2)[2,2]*50))
```

A data.frame: 12 × 5

| tp | test | estimate\_2sls | lb\_2sls | ub\_2sls |
| --- | --- | --- | --- | --- |
| <chr> | <chr> | <dbl> | <dbl> | <dbl> |
| F2 | MMSE | 0.1781228 | -0.6104052 | 0.9666509 |
| F2 | CERAD | 0.1495755 | -0.4291648 | 0.7283158 |
| F2 | DO40 | 0.3354798 | -0.2879669 | 0.9589265 |
| F2 | MEMORY | -0.9842848 | -3.6655254 | 1.6969558 |
| F2 | VERBAL | 0.9621124 | -2.7131587 | 4.6373835 |
| F2 | STROOP | 0.5023392 | -0.4329410 | 1.4376194 |
| F3 | MMSE | -0.6225237 | -1.5445949 | 0.2995475 |
| F3 | CERAD | -0.2128948 | -1.8317457 | 1.4059561 |
| F3 | DO40 | 1.1265275 | -3.0622122 | 5.3152672 |
| F3 | MEMORY | 0.5226890 | -2.7001577 | 3.7455357 |
| F3 | VERBAL | -4.7683124 | -25.2716084 | 15.7349837 |
| F3 | STROOP | 0.1091249 | -2.0872516 | 2.3055014 |

## 2SLS with PCs sensitivity analysis¶

In [39]:

```
a2<- ivreg(F2MME ~ sex+age_b+pc1+pc2+pc3+pc4+bmi_b | nonferm_dairy_b | rs4988235 + sex+age_b+pc1+pc2+pc3+pc4+bmi_b, data = df)
b2<- ivreg(F2CERAD ~ sex+age_b+pc1+pc2+pc3+pc4+bmi_b | nonferm_dairy_b | rs4988235 +sex+age_b+pc1+pc2+pc3+pc4+bmi_b, data = df)
c2<- ivreg(F2do40 ~ sex+age_b+pc1+pc2+pc3+pc4+bmi_b | nonferm_dairy_b | rs4988235 +sex+age_b+pc1+pc2+pc3+pc4+bmi_b, data = df)
d2<- ivreg(F2memory ~ sex+age_b+pc1+pc2+pc3+pc4+bmi_b | nonferm_dairy_b | rs4988235+sex+age_b+pc1+pc2+pc3+pc4+bmi_b, data = df)
e2<- ivreg(F2verbal ~ sex+age_b+pc1+pc2+pc3+pc4+bmi_b | nonferm_dairy_b | rs4988235+sex+age_b+pc1+pc2+pc3+pc4+bmi_b, data = df)
f2<- ivreg(F2stroop ~ sex+age_b+pc1+pc2+pc3+pc4+bmi_b | nonferm_dairy_b | rs4988235+sex+age_b+pc1+pc2+pc3+pc4+bmi_b, data = df)

g2<- ivreg(F3MME ~ sex+age_b+pc1+pc2+pc3+pc4+bmi_b | nonferm_dairy_b | rs4988235 +sex+age_b+pc1+pc2+pc3+pc4+bmi_b, data = df)
h2<- ivreg(F3CERAD ~ sex+age_b+pc1+pc2+pc3+pc4+bmi_b | nonferm_dairy_b | rs4988235 +sex+age_b+pc1+pc2+pc3+pc4+bmi_b, data = df)
i2<- ivreg(F3do40 ~ sex+age_b+pc1+pc2+pc3+pc4+bmi_b | nonferm_dairy_b | rs4988235 + sex+age_b+pc1+pc2+pc3+pc4+bmi_b, data = df)
j2<- ivreg(F3memory ~ sex+age_b+pc1+pc2+pc3+pc4+bmi_b | nonferm_dairy_b | rs4988235 + sex+age_b+pc1+pc2+pc3+pc4+bmi_b, data = df)
k2<- ivreg(F3verbal ~ sex+age_b+pc1+pc2+pc3+pc4+bmi_b | nonferm_dairy_b | rs4988235 + sex+age_b+pc1+pc2+pc3+pc4+bmi_b, data = df)
l2<- ivreg(F3stroop ~ sex+age_b+pc1+pc2+pc3+pc4+bmi_b | nonferm_dairy_b | rs4988235 + sex+age_b+pc1+pc2+pc3+pc4+bmi_b, data = df)
```

In [40]:

```
data.frame(tp = c("F2","F2","F2","F2","F2","F2","F3","F3","F3","F3","F3","F3"),
          test = c("MMSE","CERAD","DO40","MEMORY","VERBAL","STROOP","MMSE","CERAD","DO40","MEMORY","VERBAL","STROOP"),
          estimate_2sls = c(a2$coefficients[2]*50,b2$coefficients[2]*50,c2$coefficients[2]*50,d2$coefficients[2]*50,
                            e2$coefficients[2]*50,f2$coefficients[2]*50,g2$coefficients[2]*50,h2$coefficients[2]*50,
                            i2$coefficients[2]*50,j2$coefficients[2]*50,k2$coefficients[2]*50,l2$coefficients[2]*50),
          lb_2sls = c(confint(a2)[2,1]*50,confint(b2)[2,1]*50,confint(c2)[2,1]*50,confint(d2)[2,1]*50,confint(e2)[2,1]*50,
                     confint(f2)[2,1]*50,confint(g2)[2,1]*50,confint(h2)[2,1]*50,confint(i2)[2,1]*50,confint(j2)[2,1]*50,
                     confint(k2)[2,1]*50,confint(l2)[2,1]*50),
          ub_2sls = c(confint(a2)[2,2]*50,confint(b2)[2,2]*50,confint(c2)[2,2]*50,confint(d2)[2,2]*50,confint(e2)[2,2]*50,
                     confint(f2)[2,2]*50,confint(g2)[2,2]*50,confint(h2)[2,2]*50,confint(i2)[2,2]*50,confint(j2)[2,2]*50,
                     confint(k2)[2,2]*50,confint(l2)[2,2]*50))
```

A data.frame: 12 × 5

| tp | test | estimate\_2sls | lb\_2sls | ub\_2sls |
| --- | --- | --- | --- | --- |
| <chr> | <chr> | <dbl> | <dbl> | <dbl> |
| F2 | MMSE | 0.01943517 | -0.7052782 | 0.7441485 |
| F2 | CERAD | -0.06034060 | -0.5782250 | 0.4575438 |
| F2 | DO40 | 0.11223700 | -0.3839738 | 0.6084478 |
| F2 | MEMORY | -0.41599827 | -2.7871882 | 1.9551917 |
| F2 | VERBAL | 0.41048505 | -3.0609330 | 3.8819031 |
| F2 | STROOP | 0.31747238 | -0.4648005 | 1.0997452 |
| F3 | MMSE | -0.75982234 | -1.6231401 | 0.1034954 |
| F3 | CERAD | -0.21171386 | -1.2873182 | 0.8638905 |
| F3 | DO40 | 0.51549760 | -0.7262759 | 1.7572711 |
| F3 | MEMORY | 0.37464211 | -1.2830032 | 2.0322875 |
| F3 | VERBAL | -4.23724190 | -15.8996086 | 7.4251248 |
| F3 | STROOP | -0.15769995 | -1.5302370 | 1.2148371 |

In [41]:

```
a2<- ivreg(F2MME ~ sex+age_b+pc1+pc2+pc3+pc4+bmi_b | total_dairy_b | rs4988235 + sex+age_b+pc1+pc2+pc3+pc4+bmi_b, data = df)
b2<- ivreg(F2CERAD ~ sex+age_b+pc1+pc2+pc3+pc4+bmi_b | total_dairy_b | rs4988235 +sex+age_b+pc1+pc2+pc3+pc4+bmi_b, data = df)
c2<- ivreg(F2do40 ~ sex+age_b+pc1+pc2+pc3+pc4+bmi_b | total_dairy_b | rs4988235 +sex+age_b+pc1+pc2+pc3+pc4+bmi_b, data = df)
d2<- ivreg(F2memory ~ sex+age_b+pc1+pc2+pc3+pc4+bmi_b | total_dairy_b | rs4988235+sex+age_b+pc1+pc2+pc3+pc4+bmi_b, data = df)
e2<- ivreg(F2verbal ~ sex+age_b+pc1+pc2+pc3+pc4+bmi_b | total_dairy_b | rs4988235+sex+age_b+pc1+pc2+pc3+pc4+bmi_b, data = df)
f2<- ivreg(F2stroop ~ sex+age_b+pc1+pc2+pc3+pc4+bmi_b | total_dairy_b | rs4988235+sex+age_b+pc1+pc2+pc3+pc4+bmi_b, data = df)

g2<- ivreg(F3MME ~ sex+age_b+pc1+pc2+pc3+pc4+bmi_b | total_dairy_b | rs4988235 +sex+age_b+pc1+pc2+pc3+pc4+bmi_b, data = df)
h2<- ivreg(F3CERAD ~ sex+age_b+pc1+pc2+pc3+pc4+bmi_b | total_dairy_b | rs4988235 +sex+age_b+pc1+pc2+pc3+pc4+bmi_b, data = df)
i2<- ivreg(F3do40 ~ sex+age_b+pc1+pc2+pc3+pc4+bmi_b | total_dairy_b | rs4988235 + sex+age_b+pc1+pc2+pc3+pc4+bmi_b, data = df)
j2<- ivreg(F3memory ~ sex+age_b+pc1+pc2+pc3+pc4+bmi_b | total_dairy_b | rs4988235 + sex+age_b+pc1+pc2+pc3+pc4+bmi_b, data = df)
k2<- ivreg(F3verbal ~ sex+age_b+pc1+pc2+pc3+pc4+bmi_b | total_dairy_b | rs4988235 + sex+age_b+pc1+pc2+pc3+pc4+bmi_b, data = df)
l2<- ivreg(F3stroop ~ sex+age_b+pc1+pc2+pc3+pc4+bmi_b | total_dairy_b | rs4988235 + sex+age_b+pc1+pc2+pc3+pc4+bmi_b, data = df)

data.frame(tp = c("F2","F2","F2","F2","F2","F2", "F3","F3","F3","F3","F3","F3"),
          test = c("MMSE","CERAD","DO40","MEMORY","VERBAL","STROOP","MMSE","CERAD","DO40","MEMORY","VERBAL","STROOP"),
          estimate_2sls = c(a2$coefficients[2]*50,b2$coefficients[2]*50,c2$coefficients[2]*50,d2$coefficients[2]*50,
                            e2$coefficients[2]*50,f2$coefficients[2]*50,g2$coefficients[2]*50,h2$coefficients[2]*50,
                            i2$coefficients[2]*50,j2$coefficients[2]*50,k2$coefficients[2]*50,l2$coefficients[2]*50),
          lb_2sls = c(confint(a2)[2,1]*50,confint(b2)[2,1]*50,confint(c2)[2,1]*50,confint(d2)[2,1]*50,confint(e2)[2,1]*50,
                     confint(f2)[2,1]*50,confint(g2)[2,1]*50,confint(h2)[2,1]*50,confint(i2)[2,1]*50,confint(j2)[2,1]*50,
                     confint(k2)[2,1]*50,confint(l2)[2,1]*50),
          ub_2sls = c(confint(a2)[2,2]*50,confint(b2)[2,2]*50,confint(c2)[2,2]*50,confint(d2)[2,2]*50,confint(e2)[2,2]*50,
                     confint(f2)[2,2]*50,confint(g2)[2,2]*50,confint(h2)[2,2]*50,confint(i2)[2,2]*50,confint(j2)[2,2]*50,
                     confint(k2)[2,2]*50,confint(l2)[2,2]*50))
```

A data.frame: 12 × 5

| tp | test | estimate\_2sls | lb\_2sls | ub\_2sls |
| --- | --- | --- | --- | --- |
| <chr> | <chr> | <dbl> | <dbl> | <dbl> |
| F2 | MMSE | 0.02353999 | -0.8532918 | 0.9003718 |
| F2 | CERAD | -0.07700698 | -0.7421794 | 0.5881655 |
| F2 | DO40 | 0.16174859 | -0.6225507 | 0.9460478 |
| F2 | MEMORY | -0.63169110 | -4.4115747 | 3.1481925 |
| F2 | VERBAL | 0.67116167 | -5.2541587 | 6.5964820 |
| F2 | STROOP | 0.51069297 | -1.2186948 | 2.2400807 |
| F3 | MMSE | -0.78992796 | -1.9657753 | 0.3859194 |
| F3 | CERAD | -0.21593261 | -1.4162810 | 0.9844157 |
| F3 | DO40 | 0.60692027 | -1.8729824 | 3.0868229 |
| F3 | MEMORY | 0.49245970 | -2.4274583 | 3.4123777 |
| F3 | VERBAL | -5.21892086 | -28.3213154 | 17.8834737 |
| F3 | STROOP | -0.19010712 | -1.9889121 | 1.6086979 |

### Positive control exposure¶

In [42]:

```
a<- lm(F2MME ~ rs429358+sex+age_b+F1ht+bmi_b, data = df)
b<- lm(F2CERAD ~ rs429358+sex+age_b+F1ht+bmi_b, data = df)
c<- lm(F2do40 ~ rs429358+sex+age_b+F1ht+bmi_b, data = df)
d<- lm(F2memory ~ rs429358+sex+age_b+F1ht+bmi_b, data = df)
e<- lm(F2verbal ~ rs429358+sex+age_b+F1ht+bmi_b, data = df)
f<- lm(F2stroop ~ rs429358+sex+age_b+F1ht+bmi_b, data = df)

length(fitted(a))

g<- lm(F3MME ~ rs429358+sex+age_b+F1ht+bmi_b, data = df)
h<- lm(F3CERAD ~ rs429358+sex+age_b+F1ht+bmi_b, data = df)
i<- lm(F3do40 ~ rs429358+sex+age_b+F1ht+bmi_b, data = df)
j<- lm(F3memory ~ rs429358+sex+age_b+F1ht+bmi_b, data = df)
k<- lm(F3verbal ~ rs429358+sex+age_b+F1ht+bmi_b, data = df)
l<- lm(F3stroop ~ rs429358+sex+age_b+F1ht+bmi_b, data = df)

data.frame(tp = c("F2","F2","F2","F2","F2","F2", "F3","F3","F3","F3","F3","F3"),
          test = c("MMSE","CERAD","DO40","MEMORY","VERBAL","STROOP","MMSE","CERAD","DO40","MEMORY","VERBAL","STROOP"),
          estimate_apoe = c(a$coefficients[2],b$coefficients[2],c$coefficients[2],d$coefficients[2],
                            e$coefficients[2],f$coefficients[2],g$coefficients[2],h$coefficients[2],
                           i$coefficients[2],j$coefficients[2],k$coefficients[2],l$coefficients[2]),
          lb_apoe = c(confint(a)[2,1],confint(b)[2,1],confint(c)[2,1],confint(d)[2,1],confint(e)[2,1],
                     confint(f)[2,1],confint(g)[2,1],confint(h)[2,1], confint(i)[2,1],confint(j)[2,1],
                      confint(k)[2,1],confint(l)[2,1]),
          ub_apoe = c(confint(a)[2,2],confint(b)[2,2],confint(c)[2,2],confint(d)[2,2],confint(e)[2,2],
                     confint(f)[2,2],confint(g)[2,2],confint(h)[2,2],confint(i)[2,2],confint(j)[2,2],
                       confint(k)[2,2],confint(l)[2,2]))
```

1186

A data.frame: 12 × 5

| tp | test | estimate\_apoe | lb\_apoe | ub\_apoe |
| --- | --- | --- | --- | --- |
| <chr> | <chr> | <dbl> | <dbl> | <dbl> |
| F2 | MMSE | -0.13037128 | -0.4175895 | 0.15684698 |
| F2 | CERAD | -0.17838846 | -0.3995390 | 0.04276212 |
| F2 | DO40 | -0.03190469 | -0.2259111 | 0.16210168 |
| F2 | MEMORY | -0.34883162 | -1.3015489 | 0.60388566 |
| F2 | VERBAL | -0.18255194 | -1.5686712 | 1.20356732 |
| F2 | STROOP | 0.05067460 | -0.2435600 | 0.34490917 |
| F3 | MMSE | -0.47935068 | -0.8015452 | -0.15715618 |
| F3 | CERAD | 0.16443417 | -0.1290840 | 0.45795235 |
| F3 | DO40 | -0.21172401 | -0.4037557 | -0.01969227 |
| F3 | MEMORY | 0.10668049 | -0.3109273 | 0.52428826 |
| F3 | VERBAL | -0.12451281 | -2.2465091 | 1.99748345 |
| F3 | STROOP | -0.27463425 | -0.6632413 | 0.11397284 |

### 2. CLSA¶

#### 2A. Function to read ped files¶

In [43]:

```
read.pedfile <- function(file, first.row=NA, coded=NULL, naVal=0, sep=" ", 
		p2g=FALSE, non.rs.IDs=FALSE, cols4ID=FALSE){
	if(!is.null(coded) && !coded %in% c("12", "AB", "1234", "ATCG"))
		stop("coded must be either '12', or 'AB', or '1234', or 'ATCG'.")
	if(is.na(first.row)){
		if(non.rs.IDs){
			rs <- readLines(file, n=2)
			tmp1 <- unlist(strsplit(rs[1], sep))
			tmp2 <- unlist(strsplit(rs[2], sep))
			first.row <- length(tmp1) == length(tmp2)
			rs <- rs[1]
		}
		else{
			rs <- readLines(file, n=1)
			first.row <- tolower(substring(rs, 1, 2)) != "rs"
		}
		read <- TRUE
		cat("NOTE: first.row has not been specified. Since the first row ",
			ifelse(first.row, "does not seem", "seems"), "\n", 
			"to contain the rs-IDs, first.row is set to ", first.row,
			".\n\n", sep="")
	}
	else
		read <- FALSE
	if(!first.row){
		if(!read)
			rs <- readLines(file, n=1)
		snpnames <- unlist(strsplit(tolower(rs), sep))
		if(!non.rs.IDs && any(substring(snpnames, 1, 2) != "rs"))
			stop("All SNP names must be rs-IDs, if non.rs.IDs=FALSE.")
		ped <- read.table(file, stringsAsFactors=FALSE, skip=1)
	}
	else{
		ped <- read.table(file, stringsAsFactors=FALSE)
		snpnames <- paste("SNP", 1:((ncol(ped) - 6) / 2), sep="")
	}
	if(any(sapply(ped, is.logical))){
		idsLogical <- which(sapply(ped, is.logical))
		for(i in idsLogical){
			if(any(!ped[,i]))
				stop("ped contains a logic variable with values TRUE and FALSE.")
			ped[,i] <- "T"
		}
	}
	n.snp <- length(snpnames)
	colnames(ped) <- c("famid", "pid", "fatid", "motid", "sex", "affected",
		paste(rep(snpnames, e=2), rep(1:2, n.snp), sep="."))
	ids.kid1 <- ped[,3] != 0
	ids.kid2 <- ped[,4] != 0
	if(any(ids.kid1 != ids.kid2))
		stop("The third and fourth column of file (containing fatid and motid)",
			"\n", "must both be either zero or non-zero.")
	if(any(duplicated(ped[,2]))){
		ped[,2] <- paste(ped[,1], ped[,2], sep="_")
		ped[ids.kid1, 3] <- paste(ped[ids.kid1, 1], ped[ids.kid1, 3], sep="_")
		ped[ids.kid2, 4] <- paste(ped[ids.kid2, 1], ped[ids.kid2, 4], sep="_")
		warning("Since the individual IDs in the second column are not unique,\n",
			"they are made unique by combining the first and second column.")
	}
	if(any(duplicated(ped[,2])))
		stop("Even after combining the first and second column, the individual IDs\n",
			"in the second column are not unique. Please make them unique in file.")
	if(!p2g)
		return(ped)
	if(is.null(coded)){
		ids.select <- sample(n.snp, min(n.snp, 20)) * 2 + 6
		tmpmat <- as.matrix(ped[,ids.select])
		tabnames <- names(table(tmpmat))
		if(all(tabnames %in% c(naVal, 1:4)))
			coded <- "1234"
		else if(all(tabnames %in% c(naVal, 1:2)))
			coded <- "12"
		else if(all(tabnames %in% c(naVal, "A", "B")))
			coded <- "AB"
		else if(all(tabnames %in% c(naVal, "A", "T", "C", "G")))
			coded <- "ATCG"
		else stop("It is not clear how the SNPs and how missing values are coded.\n",
			"Please specify coded and naVal.")
		cat("NOTE: Since coded has not been specified, it is set to \"", coded, "\".\n\n", sep="")
	}
	ped2geno(ped, snpnames=snpnames, coded=coded, naVal=naVal, cols4ID=cols4ID)
}

o <- read.pedfile(".../clsa_analysis.ped", first.row = TRUE, coded = NULL, naVal = 0, sep = " ", 
   p2g = FALSE, non.rs.IDs = FALSE, cols4ID=FALSE)
    
o$lp <- ifelse(o$SNP1.1 == "G" & o$SNP1.2 == "G", 0, 1)
o$apoe <- ifelse(o$SNP2.1 == "C" | o$SNP2.2 == "C", 1, 0)    
table(o$lp) #17.7% non-lactase persistent population
table(o$apoe)
o <- o %>% rename(ADM_GWAS3_COM = pid) %>% filter(ADM_GWAS3_COM != "CTL.CEPH1463.1" & ADM_GWAS3_COM != "CTL.AXIOM103"&
                                                  ADM_GWAS3_COM != "CTL.CEPH1347.1" & ADM_GWAS3_COM != "CTL.NA24385")
o$ADM_GWAS3_COM <- as.integer(o$ADM_GWAS3_COM)
```

```
    0     1 
 4711 21915
```

```
    0     1 
19726  6900
```

#### 2B. Loading datasets¶

In [44]:

```
baseline <- read.csv(".../CLSA/2206003_UBern_PChocanoBedoya_Baseline_CoPv7.csv")
fu1 <- read.csv(".../CLSA/2206003_UBern_PChocanoBedoya_FUP1_CoPv4.csv")
part_status <- read.csv(".../CLSA/2206003_UBern_PChocanoBedoya_ParticipantStatus_CoP_v3_Sep2022.csv")
```

#### 2C. Data recoding¶

In [45]:

```
# EXPOSURE

clsa2 <- baseline %>%
  filter(NUT_LWCS_NB_COM!= 7777&NUT_CHSE_NB_COM!= 7777& NUT_LWYG_NB_COM!= 7777&NUT_YOGR_NB_COM!= 7777&
         NUT_DAIR_NB_COM!= 7777&NUT_LFML_NB_COM!= 7777& NUT_WHML_NB_COM!= 7777&NUT_CAML_NB_COM!= 7777&
         NUT_BTTR_NB_COM!= 7777&NUT_CHOC_NB_COM!= 7777& NUT_DSRT_NB_COM!= 7777&NUT_PURE_NB_COM!= 7777&
         NUT_CALC_NB_COM!= 7777&NUT_CADR_NB_COM!= 7777& NUT_SASG_NB_COM!= 7777&NUT_PATE_NB_COM!= 7777&
         NUT_FBR_NB_COM!= 7777&NUT_BRD_NB_COM!= 7777& NUT_FISH_NB_COM!= 7777&NUT_SALT_NB_COM!= 7777&
         NUT_DRSG_NB_COM!= 7777&NUT_SAUC_NB_COM!= 7777& NUT_NUTS_NB_COM!= 7777&NUT_GREEN_NB_COM!= 9999& 
         NUT_VGOT_NB_COM!= 9999& NUT_CRRT_NB_COM!= 9999&NUT_PTTO_NB_COM!= 9999&NUT_FRIE_NB_COM!= 9999&
         NUT_LEGM_NB_COM!= 9999& NUT_FRUT_NB_COM!= 9999&NUT_MEAT_NB_COM!= 9999&NUT_MTOT_NB_COM!= 9999&
         NUT_CHCK_NB_COM!= 9999& NUT_O3EG_NB_COM!= 9999&NUT_EGGS_NB_COM!= 9999&NUT_LWCS_NB_COM!= 9999&
         NUT_CHSE_NB_COM!= 9999& NUT_LWYG_NB_COM!= 9999&NUT_YOGR_NB_COM!= 9999&  NUT_DAIR_NB_COM!= 9999&
         NUT_LFML_NB_COM!= 9999& NUT_WHML_NB_COM!= 9999&NUT_CAML_NB_COM!= 9999&  NUT_BTTR_NB_COM!= 9999&
         NUT_CHOC_NB_COM!= 9999& NUT_DSRT_NB_COM!= 9999&NUT_PURE_NB_COM!= 9999&  NUT_CALC_NB_COM!= 9999&
         NUT_CADR_NB_COM!= 9999& NUT_SASG_NB_COM!= 9999&NUT_PATE_NB_COM!= 9999&  NUT_FBR_NB_COM!= 9999&
         NUT_BRD_NB_COM!= 9999& NUT_FISH_NB_COM!= 9999&NUT_SALT_NB_COM!= 9999&  NUT_DRSG_NB_COM!= 9999&
         NUT_O3EG_NB_COM!= 9998&NUT_EGGS_NB_COM!= 9998&NUT_LWCS_NB_COM!= 9998&NUT_CHSE_NB_COM!= 9998& 
         NUT_LWYG_NB_COM!= 9998&NUT_YOGR_NB_COM!= 9998&NUT_DAIR_NB_COM!= 9998&NUT_LFML_NB_COM!= 9998& 
         NUT_WHML_NB_COM!= 9998&NUT_CAML_NB_COM!= 9998&NUT_BTTR_NB_COM!= 9998&NUT_CHOC_NB_COM!= 9998&
         NUT_DSRT_NB_COM!= 9998&NUT_PURE_NB_COM!= 9998&NUT_CALC_NB_COM!= 9998&NUT_CADR_NB_COM!= 9998&
         NUT_SASG_NB_COM!= 9998&NUT_PATE_NB_COM!= 9998&NUT_FBR_NB_COM!= 9998&NUT_BRD_NB_COM!= 9998)

clsa2[clsa2 == 9996] <- 0

clsa2$nonferm_dairy<-clsa2$NUT_DAIR_NB_COM+clsa2$NUT_LFML_NB_COM+clsa2$NUT_WHML_NB_COM+clsa2$NUT_CAML_NB_COM+
                     clsa2$NUT_BTTR_NB_COM

clsa2$total_dairy<-clsa2$NUT_DAIR_NB_COM+clsa2$NUT_LFML_NB_COM+clsa2$NUT_WHML_NB_COM+clsa2$NUT_CAML_NB_COM+
                     clsa2$NUT_BTTR_NB_COM+clsa2$NUT_LWCS_NB_COM+clsa2$NUT_CHSE_NB_COM+clsa2$NUT_LWYG_NB_COM+
                     clsa2$NUT_YOGR_NB_COM+clsa2$NUT_CALC_NB_COM+clsa2$NUT_DAIR_NB_COM

clsa2$nobutternonferm_dairy<-clsa2$NUT_DAIR_NB_COM+clsa2$NUT_LFML_NB_COM+clsa2$NUT_WHML_NB_COM+clsa2$NUT_CAML_NB_COM

ch <- dplyr::left_join(clsa2, fu1, by = c("entity_id"))
ch <- ch %>% 
    dplyr::left_join(part_status, by = c("entity_id")) %>% 
    dplyr::select(-entity_id) %>% 
    dplyr::left_join(o, by = c("ADM_GWAS3_COM"))

#OUTCOME
ch <- ch %>% dplyr::rename(MAT_b = COG_MAT_SCORE_COM,
                           F1MAT = COG_MAT_SCORE_COF1,
                           REY1_b = COG_REYI_SCORE_COM,
                           F1REY1 = COG_REYI_SCORE_COF1,
                           F1VERBAL1 = COG_AFT_SCORE_1_COF1,
                           VERBAL1_b = COG_AFT_SCORE_1_COM,
                           F1VERBAL2 =COG_AFT_SCORE_2_COF1,
                           VERBAL2_b = COG_AFT_SCORE_2_COM,
                           F1REY2 = COG_REYII_SCORE_COF1,
                           REY2_b = COG_REYII_SCORE_COM,
                          age_b= AGE_NMBR_COM,
                          depre_b = DEP_DPSFD_COM, #depression
                          famincome_b = INC_TOT_COM) #family income, we could further collapse the categories

#COVARIATES
ch$sex <- ifelse(ch$SEX_ASK_COM == "M", 1, 0)

ch <- ch %>%
    dplyr::mutate(age_cat = as.factor(ifelse(age_b < 70, "Below 70",
                                  ifelse(age_b < 75, "Between 70 and 74",
                                        ifelse(age_b >= 75, "Over 74", NA)))))

ch$alc_b <- ifelse(ch$ALC_FREQ_COM >6, 0, 
                ifelse(ch$ALC_FREQ_COM > 1, 1, 
                    ifelse(ch$ALC_FREQ_COM <= 1 ,2,NA)))
ch <- ch %>% mutate(famincome_cat = ifelse(famincome_b < 3 , "Low", 
                                       ifelse(famincome_b == 3 , "Medium",
                                             ifelse(famincome_b >= 4 , "High", NA))))


ch <- mutate(ch, diab_b = ifelse(DIA_DIAB_COM == 8, NA, 
                                 ifelse(DIA_DIAB_COM == 1, 1, 0)))

ch <- mutate(ch, edu = ifelse(ED_HIGH_COM == 1, "Elementary",
                              ifelse(ED_HIGH_COM >1 & ED_HIGH_COM<5, "High school",
                                     ifelse(ED_HIGH_COM >= 5 & ED_HIGH_COM <7 , "Superior", NA))))

ch <- mutate(ch, HTA_b = ifelse(CCC_HBP_COM == 1, 1, 0))

ch$sm_b <- ifelse(ch$ICQ_SMOKE_COM == 1,"Current",
                 ifelse(ch$ICQ_SMOKE_COM == 2,"Never",
                       ifelse(ch$ICQ_SMOKE_COM == 3, "Former", NA)))

ch$bmi_b <- ifelse(ch$HWT_DBMI_COM < 25,"Normal",
                       ifelse(ch$HWT_DBMI_COM <30, "Overweight", 
                              ifelse(ch$HWT_DBMI_COM >= 30, "Obese", NA)))

ch$cvevent_b <- ifelse(ch$CCC_ANGI_COM == 1|ch$CCC_CVA_COM ==1 |
                       ch$CCC_AMI_COM == 1|ch$IHD_CAB_COM == 1 | ch$CCC_TIA_COM == 1, 1,0)


# Find tertiles
vTert = filter(ch, PA2_DSCR2_MCQ != -888) 
vTert = quantile(na.omit(vTert$PA2_DSCR2_MCQ), c(0:3/3))

ch <- ch %>% mutate(pa_b = ifelse(PA2_DSCR2_MCQ < 102.57, "Low", 
                                       ifelse(PA2_DSCR2_MCQ < 161.71, "Medium",
                                             ifelse(PA2_DSCR2_MCQ >= 161.71, "High", NA))))

# Find tertiles
vTert = filter(ch, famincome_b != -888 & famincome_b < 6) 
vTert = quantile(na.omit(vTert$famincome_b), c(0:3/3))

#OUTCOME

ch$MAT_b <- as.numeric(ch$MAT_b)
ch$F1MAT <- as.numeric(ch$F1MAT)

#Memory#

ch$F1TMT <- ifelse(ch$TMT_ITPEXACT_COF1 == -99999|ch$TMT_ITPEXACT_COF1 == -99991 |
                 ch$TMT_ITPEXACT_COF1 == -88888|ch$TMT_ITPEXACT_COF1 ==-88880 |
                ch$TMT_ACC_COF1 == -99999|ch$TMT_ACC_COF1 == -99991 |
                 ch$TMT_ACC_COF1 == -88888|ch$TMT_ACC_COF1 ==-88880 |
                ch$TMT_RMD_COF1 == -99999|ch$TMT_RMD_COF1 == -99991 |
                 ch$TMT_RMD_COF1 == -88888|ch$TMT_RMD_COF1 ==-88880, NA,
                ch$TMT_RMD_COF1+ch$TMT_ACC_COF1+ch$TMT_ITPEXACT_COF1)
ch$TMT_b <- ifelse(ch$TMT_ITPEXACT_COM == -99999|ch$TMT_ITPEXACT_COM == -99991 |
                 ch$TMT_ITPEXACT_COM == -88888|ch$TMT_ITPEXACT_COM ==-88880 |
                ch$TMT_ACC_COM == -99999|ch$TMT_ACC_COM == -99991 |
                 ch$TMT_ACC_COM == -88888|ch$TMT_ACC_COM ==-88880 |
                ch$TMT_RMD_COM == -99999|ch$TMT_RMD_COM == -99991 |
                 ch$TMT_RMD_COM == -88888|ch$TMT_RMD_COM ==-88880, NA,
                ch$TMT_RMD_COM+ch$TMT_ACC_COM+ch$TMT_ITPEXACT_COM)

ch$F1PMT <- ifelse(ch$PMT_ITP_COF1 == -99999|ch$PMT_ITP_COF1 == -99991 |
                 ch$PMT_ITP_COF1 == -88888|ch$PMT_ITP_COF1 ==-88880 |
                ch$PMT_ACR_COF1 == -99999|ch$PMT_ACR_COF1 == -99991 |
                 ch$PMT_ACR_COF1 == -88888|ch$PMT_ACR_COF1 ==-88880 |
                ch$PMT_REM_COF1 == -99999|ch$PMT_REM_COF1 == -99991 |
                 ch$PMT_REM_COF1 == -88888|ch$PMT_REM_COF1 ==-88880, NA,
                ch$PMT_ITP_COF1+ch$PMT_ACR_COF1+ch$PMT_REM_COF1)

ch$PMT_b <- ifelse(ch$PMT_ITP_COM == -99999|ch$PMT_ITP_COM == -99991 |
                 ch$PMT_ITP_COM == -88888|ch$PMT_ITP_COM ==-88880 |
                ch$PMT_ACR_COM == -99999|ch$PMT_ACR_COM == -99991 |
                 ch$PMT_ACR_COM == -88888|ch$PMT_ACR_COM ==-88880 |
                ch$PMT_REM_COM == -99999|ch$PMT_REM_COM == -99991 |
                 ch$PMT_REM_COM == -88888|ch$PMT_REM_COM ==-88880, NA,
                ch$PMT_ITP_COM+ch$PMT_ACR_COM+ch$PMT_REM_COM)

ch$F1FAS <- ifelse(ch$FAS_S_SCORE_COF1 == -99999|ch$FAS_S_SCORE_COF1 == -99991 |
                 ch$FAS_S_SCORE_COF1 == -88888|ch$FAS_S_SCORE_COF1 ==-88880, NA, ch$FAS_S_SCORE_COF1)

ch$FAS_b <- ifelse(ch$FAS_S_SCORE_COM == -99999|ch$FAS_S_SCORE_COM == -99991 |
                 ch$FAS_S_SCORE_COM == -88888|ch$FAS_S_SCORE_COM ==-88880, NA, ch$FAS_S_SCORE_COM)

#Stroop#

ch$STP_COLTIME_SS_COF1 <- ifelse(ch$STP_COLTIME_SS_COF1 == -99999|ch$STP_COLTIME_SS_COF1 == -99991 |
                 ch$STP_COLTIME_SS_COF1 == -88888|ch$STP_COLTIME_SS_COF1 ==-88880|ch$STP_COLTIME_SS_COF1==-888, NA, ch$STP_COLTIME_SS_COF1)
ch$STP_DOTTIME_SS_COF1 <- ifelse(ch$STP_DOTTIME_SS_COF1 == -99999|ch$STP_DOTTIME_SS_COF1 == -99991 |
                 ch$STP_DOTTIME_SS_COF1 == -88888|ch$STP_DOTTIME_SS_COF1 ==-88880|ch$STP_DOTTIME_SS_COF1==-888, NA, ch$STP_DOTTIME_SS_COF1)
ch$STP_COLTIME_SS_COM <- ifelse(ch$STP_COLTIME_SS_COM == -99999|ch$STP_COLTIME_SS_COM == -99991 |
                 ch$STP_COLTIME_SS_COM == -88888|ch$STP_COLTIME_SS_COM ==-88880|ch$STP_COLTIME_SS_COM==-888, NA, ch$STP_COLTIME_SS_COM)
ch$STP_DOTTIME_SS_COM <- ifelse(ch$STP_DOTTIME_SS_COM == -99999|ch$STP_DOTTIME_SS_COM == -99991 |
                 ch$STP_DOTTIME_SS_COM == -88888|ch$STP_DOTTIME_SS_COM ==-88880|ch$STP_DOTTIME_SS_COM==-888, NA, ch$STP_DOTTIME_SS_COM)

ch$F1STROOP <- ch$STP_COLTIME_SS_COF1- ch$STP_DOTTIME_SS_COF1
ch$STROOP_b <- ch$STP_COLTIME_SS_COM-ch$STP_DOTTIME_SS_COM

#REYII#

ch$F1REY1 <- ifelse(ch$F1REY1 == -99999|ch$F1REY1 == -88888, NA, ch$F1REY1)
ch$F1REY2 <-ifelse(ch$F1REY2 == -99999|ch$F1REY2 == -88888, NA, ch$F1REY2)

#MAT#

ch$F1MAT <- ifelse(ch$F1MAT == -99999|ch$F1MAT == -99991 |
                 ch$F1MAT == -88888|ch$F1MAT ==-88880, NA, ch$F1MAT)

#summary(clsa2$F1MAT)

ch$F1VERBAL2 <- ifelse(ch$F1VERBAL2 == -99999|ch$F1VERBAL2 == -99991 |
                 ch$F1VERBAL2 == -88888|ch$F1VERBAL2 ==-88880, NA, ch$F1VERBAL2)

ch$F1VERBAL1 <- ifelse(ch$F1VERBAL1 == -99999|ch$F1VERBAL1 == -99991 |
                 ch$F1VERBAL1 == -88888|ch$F1VERBAL1 ==-88880, NA, ch$F1VERBAL1)
```

#### 2D. Table 1 - Baseline characteristics¶

In [46]:

```
## Vector of variables to summarize
baselinevars <- c("sex", "age_cat", "edu","bmi_b", "sm_b","cvevent_b","HTA_b", "diab_b","pa_b", "MAT_b","nonferm_dairy")

## Vector of categorical variables that need transformation
catvars <- c("sex", "age_cat", "edu","bmi_b", "sm_b","cvevent_b","HTA_b", "diab_b","pa_b")

## Create the table object
tab1 <- CreateTableOne(vars = baselinevars, data = ch, factorVars = catvars, test = FALSE)
tab1Mat <- print(tab1, smd = TRUE)
#tab1_csv <- write.csv(tab1Mat, file = "C:/Users/no22t395/OneDrive - Universitaet Bern/PhD project/Aim 4 - MR/CLSATable1_MR.csv")

tab1_str <- CreateTableOne(vars = baselinevars, strata = "lp", data = ch, factorVars = catvars, test = FALSE)     
tab1Mat_str <- print(tab1_str, smd = TRUE)
#tab1_csv_str <- write.csv(tab1Mat_str, file = "C:/Users/no22t395/OneDrive - Universitaet Bern/PhD project/Aim 4 - MR/CLSATable1_MRstrat.csv")
```

```
                            Overall      
  n                         29046        
  sex = 1 (%)               14182 (48.8) 
  age_cat (%)                            
     Below 70               21073 (72.6) 
     Between 70 and 74       2927 (10.1) 
     Over 74                 5046 (17.4) 
  edu (%)                                
     Elementary              2142 ( 8.7) 
     High school             9424 (38.3) 
     Superior               13058 (53.0) 
  bmi_b (%)                              
     Normal                  8763 (30.3) 
     Obese                   8495 (29.4) 
     Overweight             11661 (40.3) 
  sm_b (%)                               
     Current                 2602 ( 9.0) 
     Former                 12669 (43.6) 
     Never                  13774 (47.4) 
  cvevent_b = 1 (%)          3566 (12.3) 
  HTA_b = 1 (%)             10685 (36.9) 
  diab_b = 1 (%)             5122 (17.7) 
  pa_b (%)                               
     High                    9233 (33.2) 
     Low                     9407 (33.9) 
     Medium                  9148 (32.9) 
  MAT_b (mean (SD))         26.57 (8.74) 
  nonferm_dairy (mean (SD))  1.67 (1.20) 
                           Stratified by lp
                            0             1             SMD   
  n                          4520         21186               
  sex = 1 (%)                2339 (51.7)  10475 (49.4)   0.046
  age_cat (%)                                            0.041
     Below 70                3365 (74.4)  15386 (72.6)        
     Between 70 and 74        427 ( 9.4)   2136 (10.1)        
     Over 74                  728 (16.1)   3664 (17.3)        
  edu (%)                                                0.045
     Elementary               312 ( 8.1)   1574 ( 8.8)        
     High school             1415 (36.8)   6901 (38.4)        
     Superior                2116 (55.1)   9496 (52.8)        
  bmi_b (%)                                              0.061
     Normal                  1407 (31.3)   6301 (29.8)        
     Obese                   1206 (26.8)   6241 (29.6)        
     Overweight              1886 (41.9)   8577 (40.6)        
  sm_b (%)                                               0.052
     Current                  398 ( 8.8)   1882 ( 8.9)        
     Former                  1882 (41.6)   9341 (44.1)        
     Never                   2240 (49.6)   9963 (47.0)        
  cvevent_b = 1 (%)           523 (11.6)   2561 (12.1)   0.016
  HTA_b = 1 (%)              1639 (36.3)   7803 (36.9)   0.011
  diab_b = 1 (%)              833 (18.5)   3653 (17.3)   0.032
  pa_b (%)                                               0.007
     High                    1449 (33.5)   6843 (33.6)        
     Low                     1450 (33.6)   6762 (33.2)        
     Medium                  1420 (32.9)   6734 (33.1)        
  MAT_b (mean (SD))         25.73 (8.79)  26.86 (8.70)   0.129
  nonferm_dairy (mean (SD))  1.44 (1.08)   1.73 (1.22)   0.249
```

## Ratio method¶

In [47]:

```
x <- lm(nonferm_dairy ~ lp+sex + age_b + HGT_HEIGHT_M_COM+bmi_b, data = ch)
confint(x)
```

A matrix: 7 × 2 of type dbl

|  | 2.5 % | 97.5 % |
| --- | --- | --- |
| (Intercept) | -0.548683812 | 0.23404411 |
| lp | 0.228361442 | 0.30506366 |
| sex | -0.008933154 | 0.07598639 |
| age\_b | 0.013221211 | 0.01616499 |
| HGT\_HEIGHT\_M\_COM | 0.145717164 | 0.58855228 |
| bmi\_bObese | 0.063808384 | 0.13970603 |
| bmi\_bOverweight | 0.031543251 | 0.10200970 |

In [48]:

```
#Request crude estimates Reviewer 1

summary(lm(F1MAT ~ lp, data = ch))
confint(lm(F1MAT ~ lp, data = ch))
summary(lm(F1REY1 ~ lp, data = ch))
confint(lm(F1REY1 ~ lp, data = ch))
summary(lm(F1REY2 ~ lp, data = ch))
confint(lm(F1REY2 ~ lp, data = ch))
summary(lm(F1FAS ~ lp, data = ch))
confint(lm(F1FAS ~ lp, data = ch))
summary(lm(F1PMT ~ lp, data = ch))
confint(lm(F1PMT ~ lp, data = ch))
summary(lm(F1VERBAL1 ~ lp, data = ch))
confint(lm(F1VERBAL1 ~ lp, data = ch))

summary(lm(F1MAT ~ nonferm_dairy, data = ch))
confint(lm(F1MAT ~ nonferm_dairy, data = ch))
summary(lm(F1REY1 ~ nonferm_dairy, data = ch))
confint(lm(F1REY1 ~ nonferm_dairy, data = ch))
summary(lm(F1REY2 ~ nonferm_dairy, data = ch))
confint(lm(F1REY2 ~ nonferm_dairy, data = ch))
summary(lm(F1FAS ~ nonferm_dairy, data = ch))
confint(lm(F1FAS ~ nonferm_dairy, data = ch))
summary(lm(F1PMT ~ nonferm_dairy, data = ch))
confint(lm(F1PMT ~ nonferm_dairy, data = ch))
summary(lm(F1VERBAL1 ~ nonferm_dairy, data = ch))
confint(lm(F1VERBAL1 ~ nonferm_dairy, data = ch))

summary(lm(F1MAT ~ total_dairy, data = ch))
confint(lm(F1MAT ~ total_dairy, data = ch))
summary(lm(F1REY1 ~ total_dairy, data = ch))
confint(lm(F1REY1 ~ total_dairy, data = ch))
summary(lm(F1REY2 ~ total_dairy, data = ch))
confint(lm(F1REY2 ~ total_dairy, data = ch))
summary(lm(F1FAS ~ total_dairy, data = ch))
confint(lm(F1FAS ~ total_dairy, data = ch))
summary(lm(F1PMT ~ total_dairy, data = ch))
confint(lm(F1PMT ~ total_dairy, data = ch))
summary(lm(F1VERBAL1 ~ total_dairy, data = ch))
confint(lm(F1VERBAL1 ~ total_dairy, data = ch))
```

```
Call:
lm(formula = F1MAT ~ lp, data = ch)

Residuals:
     Min       1Q   Median       3Q      Max 
-25.6686  -5.6686   0.3314   5.3314  25.1054 

Coefficients:
            Estimate Std. Error t value Pr(>|t|)    
(Intercept)  25.8946     0.1308 197.942  < 2e-16 ***
lp            0.7740     0.1439   5.381  7.5e-08 ***
---
Signif. codes:  0 '***' 0.001 '**' 0.01 '*' 0.05 '.' 0.1 ' ' 1

Residual standard error: 7.678 on 19915 degrees of freedom
  (9129 Beobachtungen als fehlend gelöscht)
Multiple R-squared:  0.001452,	Adjusted R-squared:  0.001402 
F-statistic: 28.95 on 1 and 19915 DF,  p-value: 7.502e-08
```

A matrix: 2 × 2 of type dbl

|  | 2.5 % | 97.5 % |
| --- | --- | --- |
| (Intercept) | 25.6382129 | 26.151047 |
| lp | 0.4920612 | 1.055979 |

```
Call:
lm(formula = F1REY1 ~ lp, data = ch)

Residuals:
    Min      1Q  Median      3Q     Max 
-6.6188 -1.6188  0.3812  1.3812  8.5710 

Coefficients:
            Estimate Std. Error t value Pr(>|t|)    
(Intercept)  6.42897    0.03685 174.468  < 2e-16 ***
lp           0.18978    0.04056   4.679 2.91e-06 ***
---
Signif. codes:  0 '***' 0.001 '**' 0.01 '*' 0.05 '.' 0.1 ' ' 1

Residual standard error: 2.21 on 20576 degrees of freedom
  (8468 Beobachtungen als fehlend gelöscht)
Multiple R-squared:  0.001063,	Adjusted R-squared:  0.001014 
F-statistic: 21.89 on 1 and 20576 DF,  p-value: 2.908e-06
```

A matrix: 2 × 2 of type dbl

|  | 2.5 % | 97.5 % |
| --- | --- | --- |
| (Intercept) | 6.3567416 | 6.5011955 |
| lp | 0.1102722 | 0.2692913 |

```
Call:
lm(formula = F1REY2 ~ lp, data = ch)

Residuals:
    Min      1Q  Median      3Q     Max 
-4.7182 -1.7182  0.2818  1.2818 10.4170 

Coefficients:
            Estimate Std. Error t value Pr(>|t|)    
(Intercept)  4.58303    0.04025 113.863  < 2e-16 ***
lp           0.13520    0.04431   3.051  0.00228 ** 
---
Signif. codes:  0 '***' 0.001 '**' 0.01 '*' 0.05 '.' 0.1 ' ' 1

Residual standard error: 2.405 on 20434 degrees of freedom
  (8610 Beobachtungen als fehlend gelöscht)
Multiple R-squared:  0.0004555,	Adjusted R-squared:  0.0004066 
F-statistic: 9.312 on 1 and 20434 DF,  p-value: 0.00228
```

A matrix: 2 × 2 of type dbl

|  | 2.5 % | 97.5 % |
| --- | --- | --- |
| (Intercept) | 4.50413623 | 4.6619237 |
| lp | 0.04835758 | 0.2220486 |

```
Call:
lm(formula = F1FAS ~ lp, data = ch)

Residuals:
     Min       1Q   Median       3Q      Max 
-13.3105  -3.3105  -0.3105   2.6895  26.6895 

Coefficients:
            Estimate Std. Error t value Pr(>|t|)    
(Intercept) 13.69956    0.07822 175.146  < 2e-16 ***
lp           0.61097    0.08611   7.095 1.33e-12 ***
---
Signif. codes:  0 '***' 0.001 '**' 0.01 '*' 0.05 '.' 0.1 ' ' 1

Residual standard error: 4.867 on 22128 degrees of freedom
  (6916 Beobachtungen als fehlend gelöscht)
Multiple R-squared:  0.00227,	Adjusted R-squared:  0.002225 
F-statistic: 50.34 on 1 and 22128 DF,  p-value: 1.331e-12
```

A matrix: 2 × 2 of type dbl

|  | 2.5 % | 97.5 % |
| --- | --- | --- |
| (Intercept) | 13.5462483 | 13.8528733 |
| lp | 0.4421875 | 0.7797544 |

```
Call:
lm(formula = F1PMT ~ lp, data = ch)

Residuals:
    Min      1Q  Median      3Q     Max 
-8.6080  0.3920  0.3920  0.3920  0.5588 

Coefficients:
            Estimate Std. Error t value Pr(>|t|)    
(Intercept)  8.44118    0.02006 420.692  < 2e-16 ***
lp           0.16683    0.02210   7.548 4.59e-14 ***
---
Signif. codes:  0 '***' 0.001 '**' 0.01 '*' 0.05 '.' 0.1 ' ' 1

Residual standard error: 1.249 on 22040 degrees of freedom
  (7004 Beobachtungen als fehlend gelöscht)
Multiple R-squared:  0.002578,	Adjusted R-squared:  0.002533 
F-statistic: 56.97 on 1 and 22040 DF,  p-value: 4.59e-14
```

A matrix: 2 × 2 of type dbl

|  | 2.5 % | 97.5 % |
| --- | --- | --- |
| (Intercept) | 8.4018477 | 8.4805052 |
| lp | 0.1235057 | 0.2101493 |

```
Call:
lm(formula = F1VERBAL1 ~ lp, data = ch)

Residuals:
     Min       1Q   Median       3Q      Max 
-18.8445  -3.8445   0.1555   3.1555  24.1555 

Coefficients:
            Estimate Std. Error t value Pr(>|t|)    
(Intercept) 19.08858    0.08927 213.824  < 2e-16 ***
lp           0.75597    0.09832   7.689 1.55e-14 ***
---
Signif. codes:  0 '***' 0.001 '**' 0.01 '*' 0.05 '.' 0.1 ' ' 1

Residual standard error: 5.382 on 20706 degrees of freedom
  (8338 Beobachtungen als fehlend gelöscht)
Multiple R-squared:  0.002847,	Adjusted R-squared:  0.002799 
F-statistic: 59.12 on 1 and 20706 DF,  p-value: 1.548e-14
```

A matrix: 2 × 2 of type dbl

|  | 2.5 % | 97.5 % |
| --- | --- | --- |
| (Intercept) | 18.9136027 | 19.2635638 |
| lp | 0.5632568 | 0.9486765 |

```
Call:
lm(formula = F1MAT ~ nonferm_dairy, data = ch)

Residuals:
     Min       1Q   Median       3Q      Max 
-25.8219  -5.3560   0.1809   5.1809  25.2743 

Coefficients:
              Estimate Std. Error t value Pr(>|t|)    
(Intercept)   26.94546    0.08848 304.529  < 2e-16 ***
nonferm_dairy -0.29475    0.04359  -6.762  1.4e-11 ***
---
Signif. codes:  0 '***' 0.001 '**' 0.01 '*' 0.05 '.' 0.1 ' ' 1

Residual standard error: 7.683 on 22317 degrees of freedom
  (6727 Beobachtungen als fehlend gelöscht)
Multiple R-squared:  0.002044,	Adjusted R-squared:  0.002 
F-statistic: 45.72 on 1 and 22317 DF,  p-value: 1.399e-11
```

A matrix: 2 × 2 of type dbl

|  | 2.5 % | 97.5 % |
| --- | --- | --- |
| (Intercept) | 26.772031 | 27.1188949 |
| nonferm\_dairy | -0.380193 | -0.2093053 |

```
Call:
lm(formula = F1REY1 ~ nonferm_dairy, data = ch)

Residuals:
    Min      1Q  Median      3Q     Max 
-6.7852 -1.5764  0.2148  1.3933  8.6252 

Coefficients:
              Estimate Std. Error t value Pr(>|t|)    
(Intercept)    6.78516    0.02494  272.01   <2e-16 ***
nonferm_dairy -0.12489    0.01230  -10.16   <2e-16 ***
---
Signif. codes:  0 '***' 0.001 '**' 0.01 '*' 0.05 '.' 0.1 ' ' 1

Residual standard error: 2.205 on 23099 degrees of freedom
  (5945 Beobachtungen als fehlend gelöscht)
Multiple R-squared:  0.004445,	Adjusted R-squared:  0.004402 
F-statistic: 103.1 on 1 and 23099 DF,  p-value: < 2.2e-16
```

A matrix: 2 × 2 of type dbl

|  | 2.5 % | 97.5 % |
| --- | --- | --- |
| (Intercept) | 6.7362692 | 6.8340546 |
| nonferm\_dairy | -0.1489939 | -0.1007856 |

```
Call:
lm(formula = F1REY2 ~ nonferm_dairy, data = ch)

Residuals:
    Min      1Q  Median      3Q     Max 
-4.9407 -1.7017  0.0913  1.3787 10.5590 

Coefficients:
              Estimate Std. Error t value Pr(>|t|)    
(Intercept)    4.94068    0.02719  181.69   <2e-16 ***
nonferm_dairy -0.15206    0.01341  -11.34   <2e-16 ***
---
Signif. codes:  0 '***' 0.001 '**' 0.01 '*' 0.05 '.' 0.1 ' ' 1

Residual standard error: 2.396 on 22940 degrees of freedom
  (6104 Beobachtungen als fehlend gelöscht)
Multiple R-squared:  0.00557,	Adjusted R-squared:  0.005527 
F-statistic: 128.5 on 1 and 22940 DF,  p-value: < 2.2e-16
```

A matrix: 2 × 2 of type dbl

|  | 2.5 % | 97.5 % |
| --- | --- | --- |
| (Intercept) | 4.8873767 | 4.9939775 |
| nonferm\_dairy | -0.1783549 | -0.1257691 |

```
Call:
lm(formula = F1FAS ~ nonferm_dairy, data = ch)

Residuals:
     Min       1Q   Median       3Q      Max 
-13.4129  -3.2671  -0.2422   2.9012  26.7531 

Coefficients:
              Estimate Std. Error t value Pr(>|t|)    
(Intercept)   14.41292    0.05292 272.343  < 2e-16 ***
nonferm_dairy -0.14112    0.02574  -5.482 4.24e-08 ***
---
Signif. codes:  0 '***' 0.001 '**' 0.01 '*' 0.05 '.' 0.1 ' ' 1

Residual standard error: 4.863 on 24765 degrees of freedom
  (4279 Beobachtungen als fehlend gelöscht)
Multiple R-squared:  0.001212,	Adjusted R-squared:  0.001172 
F-statistic: 30.06 on 1 and 24765 DF,  p-value: 4.239e-08
```

A matrix: 2 × 2 of type dbl

|  | 2.5 % | 97.5 % |
| --- | --- | --- |
| (Intercept) | 14.3091908 | 14.51665106 |
| nonferm\_dairy | -0.1915763 | -0.09066758 |

```
Call:
lm(formula = F1PMT ~ nonferm_dairy, data = ch)

Residuals:
    Min      1Q  Median      3Q     Max 
-8.5899  0.4139  0.4235  0.4327  0.6084 

Coefficients:
               Estimate Std. Error t value Pr(>|t|)    
(Intercept)    8.591600   0.013779 623.539   <2e-16 ***
nonferm_dairy -0.011763   0.006703  -1.755   0.0793 .  
---
Signif. codes:  0 '***' 0.001 '**' 0.01 '*' 0.05 '.' 0.1 ' ' 1

Residual standard error: 1.262 on 24675 degrees of freedom
  (4369 Beobachtungen als fehlend gelöscht)
Multiple R-squared:  0.0001248,	Adjusted R-squared:  8.429e-05 
F-statistic:  3.08 on 1 and 24675 DF,  p-value: 0.07927
```

A matrix: 2 × 2 of type dbl

|  | 2.5 % | 97.5 % |
| --- | --- | --- |
| (Intercept) | 8.56459282 | 8.618607266 |
| nonferm\_dairy | -0.02490047 | 0.001374173 |

```
Call:
lm(formula = F1VERBAL1 ~ nonferm_dairy, data = ch)

Residuals:
     Min       1Q   Median       3Q      Max 
-18.4219  -3.7058  -0.2568   3.3638  24.4280 

Coefficients:
              Estimate Std. Error t value Pr(>|t|)    
(Intercept)   19.92007    0.06068 328.304  < 2e-16 ***
nonferm_dairy -0.16244    0.02987  -5.439 5.43e-08 ***
---
Signif. codes:  0 '***' 0.001 '**' 0.01 '*' 0.05 '.' 0.1 ' ' 1

Residual standard error: 5.379 on 23246 degrees of freedom
  (5798 Beobachtungen als fehlend gelöscht)
Multiple R-squared:  0.001271,	Adjusted R-squared:  0.001228 
F-statistic: 29.58 on 1 and 23246 DF,  p-value: 5.425e-08
```

A matrix: 2 × 2 of type dbl

|  | 2.5 % | 97.5 % |
| --- | --- | --- |
| (Intercept) | 19.8011400 | 20.0389964 |
| nonferm\_dairy | -0.2209832 | -0.1038963 |

```
Call:
lm(formula = F1MAT ~ total_dairy, data = ch)

Residuals:
     Min       1Q   Median       3Q      Max 
-25.7418  -5.4070   0.2536   5.1995  25.1346 

Coefficients:
            Estimate Std. Error t value Pr(>|t|)    
(Intercept) 27.00268    0.11053 244.310  < 2e-16 ***
total_dairy -0.18954    0.03408  -5.562  2.7e-08 ***
---
Signif. codes:  0 '***' 0.001 '**' 0.01 '*' 0.05 '.' 0.1 ' ' 1

Residual standard error: 7.686 on 22317 degrees of freedom
  (6727 Beobachtungen als fehlend gelöscht)
Multiple R-squared:  0.001384,	Adjusted R-squared:  0.001339 
F-statistic: 30.93 on 1 and 22317 DF,  p-value: 2.703e-08
```

A matrix: 2 × 2 of type dbl

|  | 2.5 % | 97.5 % |
| --- | --- | --- |
| (Intercept) | 26.7860415 | 27.219319 |
| total\_dairy | -0.2563387 | -0.122741 |

```
Call:
lm(formula = F1REY1 ~ total_dairy, data = ch)

Residuals:
    Min      1Q  Median      3Q     Max 
-6.7378 -1.5778  0.2738  1.4125  8.5311 

Coefficients:
             Estimate Std. Error t value Pr(>|t|)    
(Intercept)  6.737786   0.031246 215.638  < 2e-16 ***
total_dairy -0.055356   0.009648  -5.737 9.73e-09 ***
---
Signif. codes:  0 '***' 0.001 '**' 0.01 '*' 0.05 '.' 0.1 ' ' 1

Residual standard error: 2.208 on 23099 degrees of freedom
  (5945 Beobachtungen als fehlend gelöscht)
Multiple R-squared:  0.001423,	Adjusted R-squared:  0.00138 
F-statistic: 32.92 on 1 and 23099 DF,  p-value: 9.729e-09
```

A matrix: 2 × 2 of type dbl

|  | 2.5 % | 97.5 % |
| --- | --- | --- |
| (Intercept) | 6.6765420 | 6.79902968 |
| total\_dairy | -0.0742663 | -0.03644476 |

```
Call:
lm(formula = F1REY2 ~ total_dairy, data = ch)

Residuals:
    Min      1Q  Median      3Q     Max 
-4.8902 -1.7005  0.1843  1.3594 10.5131 

Coefficients:
            Estimate Std. Error t value Pr(>|t|)    
(Intercept)  4.89023    0.03408 143.487  < 2e-16 ***
total_dairy -0.06989    0.01053  -6.638 3.26e-11 ***
---
Signif. codes:  0 '***' 0.001 '**' 0.01 '*' 0.05 '.' 0.1 ' ' 1

Residual standard error: 2.401 on 22940 degrees of freedom
  (6104 Beobachtungen als fehlend gelöscht)
Multiple R-squared:  0.001917,	Adjusted R-squared:  0.001873 
F-statistic: 44.06 on 1 and 22940 DF,  p-value: 3.255e-11
```

A matrix: 2 × 2 of type dbl

|  | 2.5 % | 97.5 % |
| --- | --- | --- |
| (Intercept) | 4.82342936 | 4.95703272 |
| total\_dairy | -0.09053069 | -0.04925387 |

```
Call:
lm(formula = F1FAS ~ total_dairy, data = ch)

Residuals:
     Min       1Q   Median       3Q      Max 
-13.3134  -3.2529  -0.2166   2.8923  26.7285 

Coefficients:
            Estimate Std. Error t value Pr(>|t|)    
(Intercept) 14.42232    0.06593 218.747  < 2e-16 ***
total_dairy -0.08470    0.02014  -4.206 2.61e-05 ***
---
Signif. codes:  0 '***' 0.001 '**' 0.01 '*' 0.05 '.' 0.1 ' ' 1

Residual standard error: 4.864 on 24765 degrees of freedom
  (4279 Beobachtungen als fehlend gelöscht)
Multiple R-squared:  0.0007138,	Adjusted R-squared:  0.0006735 
F-statistic: 17.69 on 1 and 24765 DF,  p-value: 2.609e-05
```

A matrix: 2 × 2 of type dbl

|  | 2.5 % | 97.5 % |
| --- | --- | --- |
| (Intercept) | 14.2930918 | 14.55155062 |
| total\_dairy | -0.1241762 | -0.04522955 |

```
Call:
lm(formula = F1PMT ~ total_dairy, data = ch)

Residuals:
    Min      1Q  Median      3Q     Max 
-8.5921  0.4141  0.4242  0.4331  0.6470 

Coefficients:
             Estimate Std. Error t value Pr(>|t|)    
(Intercept)  8.599537   0.017152  501.36   <2e-16 ***
total_dairy -0.009533   0.005237   -1.82   0.0687 .  
---
Signif. codes:  0 '***' 0.001 '**' 0.01 '*' 0.05 '.' 0.1 ' ' 1

Residual standard error: 1.262 on 24675 degrees of freedom
  (4369 Beobachtungen als fehlend gelöscht)
Multiple R-squared:  0.0001343,	Adjusted R-squared:  9.373e-05 
F-statistic: 3.313 on 1 and 24675 DF,  p-value: 0.06874
```

A matrix: 2 × 2 of type dbl

|  | 2.5 % | 97.5 % |
| --- | --- | --- |
| (Intercept) | 8.56591733 | 8.6331568595 |
| total\_dairy | -0.01979863 | 0.0007325389 |

```
Call:
lm(formula = F1VERBAL1 ~ total_dairy, data = ch)

Residuals:
     Min       1Q   Median       3Q      Max 
-18.4944  -3.6794  -0.3873   3.3605  24.3830 

Coefficients:
            Estimate Std. Error t value Pr(>|t|)    
(Intercept) 19.91831    0.07589  262.46  < 2e-16 ***
total_dairy -0.09293    0.02341   -3.97 7.21e-05 ***
---
Signif. codes:  0 '***' 0.001 '**' 0.01 '*' 0.05 '.' 0.1 ' ' 1

Residual standard error: 5.38 on 23246 degrees of freedom
  (5798 Beobachtungen als fehlend gelöscht)
Multiple R-squared:  0.0006775,	Adjusted R-squared:  0.0006345 
F-statistic: 15.76 on 1 and 23246 DF,  p-value: 7.214e-05
```

A matrix: 2 × 2 of type dbl

|  | 2.5 % | 97.5 % |
| --- | --- | --- |
| (Intercept) | 19.7695603 | 20.06706710 |
| total\_dairy | -0.1388063 | -0.04704435 |

In [49]:

```
o <- lm(nonferm_dairy ~ lp, data = ch)
summary(o)
confint(o)

o <- lm(nonferm_dairy ~ lp +sex + age_b + HGT_HEIGHT_M_COM+bmi_b, data = ch)
summary(o)
confint(o)

a<- lm(F1MAT ~ lp+ sex + age_b + HGT_HEIGHT_M_COM+bmi_b, data = ch)
b<- lm(F1REY1 ~ lp+ sex + age_b + HGT_HEIGHT_M_COM+bmi_b, data = ch)
c<- lm(F1REY2 ~ lp+ sex + age_b + HGT_HEIGHT_M_COM+bmi_b, data = ch)
d<- lm(F1FAS ~ lp+ sex + age_b + HGT_HEIGHT_M_COM+bmi_b, data = ch)
e<- lm(F1PMT ~ lp+ sex + age_b + HGT_HEIGHT_M_COM+bmi_b, data = ch)
f<- lm(F1TMT ~ lp+ sex + age_b + HGT_HEIGHT_M_COM+bmi_b, data = ch)
g<- lm(F1VERBAL1 ~ lp+ sex + age_b + HGT_HEIGHT_M_COM+bmi_b, data = ch)
h<- lm(F1VERBAL2 ~ lp+ sex + age_b + HGT_HEIGHT_M_COM+bmi_b, data = ch)


k <- lm(nonferm_dairy ~ apoe +sex + age_b + HGT_HEIGHT_M_COM+bmi_b, data = ch)
k

i<- lm(F1MAT ~ apoe+ sex + age_b + HGT_HEIGHT_M_COM+bmi_b, data = ch)
j<- lm(F1REY1 ~ apoe+ sex + age_b + HGT_HEIGHT_M_COM+bmi_b, data = ch)
k<- lm(F1REY2 ~ apoe+ sex + age_b + HGT_HEIGHT_M_COM+bmi_b, data = ch)
l<- lm(F1FAS ~ apoe+ sex + age_b + HGT_HEIGHT_M_COM+bmi_b, data = ch)
m<- lm(F1PMT ~ apoe+ sex + age_b + HGT_HEIGHT_M_COM+bmi_b, data = ch)
n<- lm(F1TMT ~ apoe+ sex + age_b + HGT_HEIGHT_M_COM+bmi_b, data = ch)
o<- lm(F1VERBAL1 ~ apoe+ sex + age_b + HGT_HEIGHT_M_COM+bmi_b, data = ch)
p<- lm(F1VERBAL2 ~ apoe+ sex + age_b + HGT_HEIGHT_M_COM+bmi_b, data = ch)

table = data.frame(test = c("MAT", "REYI", "REYII", "FAS", "PMT", "TMT","VERBAL1", "VERBAL2"),
                  estimate_apoe = c(i$coefficients[2],j$coefficients[2],k$coefficients[2],l$coefficients[2],
                                    m$coefficients[2],n$coefficients[2],o$coefficients[2],p$coefficients[2]),
                  lb_apoe = c(confint(i)[2,1],confint(j)[2,1],confint(k)[2,1],confint(l)[2,1],confint(m)[2,1],
                             confint(n)[2,1],confint(o)[2,1],confint(p)[2,1]),
                   ub_apoe = c(confint(i)[2,2],confint(j)[2,2],confint(k)[2,2],confint(l)[2,2],confint(m)[2,2],
                             confint(n)[2,2],confint(o)[2,2],confint(p)[2,2]))
table
```

```
Call:
lm(formula = nonferm_dairy ~ lp, data = ch)

Residuals:
    Min      1Q  Median      3Q     Max 
-1.7282 -0.7282 -0.1996  0.5575 15.2718 

Coefficients:
            Estimate Std. Error t value Pr(>|t|)    
(Intercept)  1.44247    0.01775   81.25   <2e-16 ***
lp           0.28570    0.01955   14.61   <2e-16 ***
---
Signif. codes:  0 '***' 0.001 '**' 0.01 '*' 0.05 '.' 0.1 ' ' 1

Residual standard error: 1.194 on 25704 degrees of freedom
  (3340 Beobachtungen als fehlend gelöscht)
Multiple R-squared:  0.008236,	Adjusted R-squared:  0.008198 
F-statistic: 213.5 on 1 and 25704 DF,  p-value: < 2.2e-16
```

A matrix: 2 × 2 of type dbl

|  | 2.5 % | 97.5 % |
| --- | --- | --- |
| (Intercept) | 1.4076790 | 1.4772708 |
| lp | 0.2473746 | 0.3240313 |

```
Call:
lm(formula = nonferm_dairy ~ lp + sex + age_b + HGT_HEIGHT_M_COM + 
    bmi_b, data = ch)

Residuals:
    Min      1Q  Median      3Q     Max 
-2.0899 -0.7506 -0.1905  0.5059 15.1234 

Coefficients:
                   Estimate Std. Error t value Pr(>|t|)    
(Intercept)      -0.1573198  0.1996697  -0.788 0.430762    
lp                0.2667125  0.0195663  13.631  < 2e-16 ***
sex               0.0335266  0.0216625   1.548 0.121712    
age_b             0.0146931  0.0007509  19.566  < 2e-16 ***
HGT_HEIGHT_M_COM  0.3671347  0.1129649   3.250 0.001156 ** 
bmi_bObese        0.1017572  0.0193611   5.256 1.49e-07 ***
bmi_bOverweight   0.0667765  0.0179756   3.715 0.000204 ***
---
Signif. codes:  0 '***' 0.001 '**' 0.01 '*' 0.05 '.' 0.1 ' ' 1

Residual standard error: 1.184 on 25611 degrees of freedom
  (3428 Beobachtungen als fehlend gelöscht)
Multiple R-squared:  0.02573,	Adjusted R-squared:  0.0255 
F-statistic: 112.7 on 6 and 25611 DF,  p-value: < 2.2e-16
```

A matrix: 7 × 2 of type dbl

|  | 2.5 % | 97.5 % |
| --- | --- | --- |
| (Intercept) | -0.548683812 | 0.23404411 |
| lp | 0.228361442 | 0.30506366 |
| sex | -0.008933154 | 0.07598639 |
| age\_b | 0.013221211 | 0.01616499 |
| HGT\_HEIGHT\_M\_COM | 0.145717164 | 0.58855228 |
| bmi\_bObese | 0.063808384 | 0.13970603 |
| bmi\_bOverweight | 0.031543251 | 0.10200970 |

```
Call:
lm(formula = nonferm_dairy ~ apoe + sex + age_b + HGT_HEIGHT_M_COM + 
    bmi_b, data = ch)

Coefficients:
     (Intercept)              apoe               sex             age_b  
       -0.251284          0.039638          0.006452          0.015356  
HGT_HEIGHT_M_COM        bmi_bObese   bmi_bOverweight  
        0.528183          0.110953          0.070012
```

A data.frame: 8 × 4

| test | estimate\_apoe | lb\_apoe | ub\_apoe |
| --- | --- | --- | --- |
| <chr> | <dbl> | <dbl> | <dbl> |
| MAT | -0.42774536 | -0.65628101 | -0.19920970 |
| REYI | -0.09519707 | -0.15646365 | -0.03393049 |
| REYII | -0.15688643 | -0.22278241 | -0.09099045 |
| FAS | 0.18735154 | 0.04341869 | 0.33128439 |
| PMT | -0.07772569 | -0.11405643 | -0.04139494 |
| TMT | -0.08139920 | -0.11242012 | -0.05037829 |
| VERBAL1 | -0.06136949 | -0.21518239 | 0.09244342 |
| VERBAL2 | -0.04204646 | -0.21766551 | 0.13357258 |

In [50]:

```
length(fitted(i))
```

19863

## 2SLS with BMI¶

In [51]:

```
ch = mutate(ch, milkpred = predict(o, newdata = ch))

a2<- ivreg(F1MAT ~ sex + age_b + HGT_HEIGHT_M_COM+bmi_b|nonferm_dairy|lp+sex+age_b+HGT_HEIGHT_M_COM+bmi_b,data = ch)
b2<- ivreg(F1REY1 ~ sex + age_b +HGT_HEIGHT_M_COM+bmi_b|nonferm_dairy|lp+sex+age_b+HGT_HEIGHT_M_COM+bmi_b,data = ch)
c2<- ivreg(F1REY2 ~ sex + age_b +HGT_HEIGHT_M_COM+bmi_b|nonferm_dairy|lp+sex+age_b+HGT_HEIGHT_M_COM+bmi_b,data = ch)
d2<- ivreg(F1FAS ~ sex + age_b + HGT_HEIGHT_M_COM+bmi_b|nonferm_dairy|lp+sex+age_b+HGT_HEIGHT_M_COM+bmi_b,data = ch)
e2<- ivreg(F1PMT ~ sex + age_b + HGT_HEIGHT_M_COM+bmi_b|nonferm_dairy|lp+sex+age_b+HGT_HEIGHT_M_COM+bmi_b,data = ch)
f2<- ivreg(F1TMT ~ sex + age_b + HGT_HEIGHT_M_COM+bmi_b|nonferm_dairy|lp+sex+age_b+HGT_HEIGHT_M_COM+bmi_b,data = ch)
g2<- ivreg(F1VERBAL1 ~sex+age_b+ HGT_HEIGHT_M_COM+bmi_b|nonferm_dairy|lp+sex+age_b+HGT_HEIGHT_M_COM+bmi_b,data = ch)
h2<- ivreg(F1VERBAL2 ~sex+age_b+ HGT_HEIGHT_M_COM+bmi_b|nonferm_dairy|lp+sex+age_b+HGT_HEIGHT_M_COM+bmi_b,data = ch)

summary(a2)
```

```
Call:
ivreg(formula = F1MAT ~ sex + age_b + HGT_HEIGHT_M_COM + bmi_b | 
    nonferm_dairy | lp + sex + age_b + HGT_HEIGHT_M_COM + bmi_b, 
    data = ch)

Residuals:
     Min       1Q   Median       3Q      Max 
-57.3020  -5.4122   0.1687   5.5330  31.2723 

Coefficients:
                 Estimate Std. Error t value Pr(>|t|)    
(Intercept)      22.75427    1.61803  14.063  < 2e-16 ***
nonferm_dairy     3.49527    0.63214   5.529 3.26e-08 ***
sex              -0.12419    0.17269  -0.719    0.472    
age_b            -0.27760    0.01114 -24.920  < 2e-16 ***
HGT_HEIGHT_M_COM  9.45975    0.97716   9.681  < 2e-16 ***
bmi_bObese       -1.14710    0.17156  -6.686 2.35e-11 ***
bmi_bOverweight  -0.70469    0.15104  -4.665 3.10e-06 ***

Diagnostic tests:
                   df1   df2 statistic  p-value    
Weak instruments     1 19856    128.69  < 2e-16 ***
Wu-Hausman           1 19855     44.11 3.19e-11 ***
Sargan               0    NA        NA       NA    
---
Signif. codes:  0 '***' 0.001 '**' 0.01 '*' 0.05 '.' 0.1 ' ' 1

Residual standard error: 8.334 on 19856 degrees of freedom
Multiple R-Squared: -0.1769,	Adjusted R-squared: -0.1773 
Wald test: 350.2 on 6 and 19856 DF,  p-value: < 2.2e-16
```

In [52]:

```
data.frame(test = c("MAT", "REYI", "REYII", "FAS", "PMT", "TMT","VERBAL1", "VERBAL2"),
           estimate_ratio = c(a$coefficients[2]/o$coefficients[2],b$coefficients[2]/o$coefficients[2],
                       c$coefficients[2]/o$coefficients[2],d$coefficients[2]/o$coefficients[2],
                       e$coefficients[2]/o$coefficients[2],f$coefficients[2]/o$coefficients[2],
                       g$coefficients[2]/o$coefficients[2],h$coefficients[2]/o$coefficients[2]),
          estimate_2sls = c(a2$coefficients[2],b2$coefficients[2],c2$coefficients[2],d2$coefficients[2],
                            e2$coefficients[2],f2$coefficients[2],g2$coefficients[2],h2$coefficients[2]),
          lb_2sls = c(confint(a2)[2,1],confint(b2)[2,1],confint(c2)[2,1],confint(d2)[2,1],confint(e2)[2,1],
                     confint(f2)[2,1],confint(g2)[2,1],confint(h2)[2,1]),
          ub_2sls = c(confint(a2)[2,2],confint(b2)[2,2],confint(c2)[2,2],confint(d2)[2,2],confint(e2)[2,2],
                     confint(f2)[2,2],confint(g2)[2,2],confint(h2)[2,2]))
```

A data.frame: 8 × 5

| test | estimate\_ratio | estimate\_2sls | lb\_2sls | ub\_2sls |
| --- | --- | --- | --- | --- |
| <chr> | <dbl> | <dbl> | <dbl> | <dbl> |
| MAT | -14.1843230 | 3.4952697 | 2.25621606 | 4.7343234 |
| REYI | -3.5800791 | 0.8910732 | 0.56306720 | 1.2190792 |
| REYII | -3.0194297 | 0.7542191 | 0.41500025 | 1.0934379 |
| FAS | -9.0963914 | 2.1748855 | 1.43024115 | 2.9195299 |
| PMT | -2.9747044 | 0.7064649 | 0.50962618 | 0.9033037 |
| TMT | -0.5474878 | 0.1281836 | -0.01050922 | 0.2668765 |
| VERBAL1 | -12.5169605 | 3.0758300 | 2.19394048 | 3.9577196 |
| VERBAL2 | -14.0847178 | 3.4646591 | 2.45867106 | 4.4706471 |

### Total dairy¶

In [53]:

```
a2<- ivreg(F1MAT ~ sex + age_b + HGT_HEIGHT_M_COM+bmi_b|total_dairy|lp+sex+age_b+HGT_HEIGHT_M_COM+bmi_b,data = ch)
b2<- ivreg(F1REY1 ~ sex + age_b +HGT_HEIGHT_M_COM+bmi_b|total_dairy|lp+sex+age_b+HGT_HEIGHT_M_COM+bmi_b,data = ch)
c2<- ivreg(F1REY2 ~ sex + age_b +HGT_HEIGHT_M_COM+bmi_b|total_dairy|lp+sex+age_b+HGT_HEIGHT_M_COM+bmi_b,data = ch)
d2<- ivreg(F1FAS ~ sex + age_b + HGT_HEIGHT_M_COM+bmi_b|total_dairy|lp+sex+age_b+HGT_HEIGHT_M_COM+bmi_b,data = ch)
e2<- ivreg(F1PMT ~ sex + age_b + HGT_HEIGHT_M_COM+bmi_b|total_dairy|lp+sex+age_b+HGT_HEIGHT_M_COM+bmi_b,data = ch)
f2<- ivreg(F1TMT ~ sex + age_b + HGT_HEIGHT_M_COM+bmi_b|total_dairy|lp+sex+age_b+HGT_HEIGHT_M_COM+bmi_b,data = ch)
g2<- ivreg(F1VERBAL1 ~sex+age_b+ HGT_HEIGHT_M_COM+bmi_b|total_dairy|lp+sex+age_b+HGT_HEIGHT_M_COM+bmi_b,data = ch)
h2<- ivreg(F1VERBAL2 ~sex+age_b+ HGT_HEIGHT_M_COM+bmi_b|total_dairy|lp+sex+age_b+HGT_HEIGHT_M_COM+bmi_b,data = ch)

data.frame(test = c("MAT", "REYI", "REYII", "FAS", "PMT", "TMT","VERBAL1", "VERBAL2"),
           estimate_ratio = c(a$coefficients[2]/x$coefficients[2],b$coefficients[2]/x$coefficients[2],
                       c$coefficients[2]/x$coefficients[2],d$coefficients[2]/x$coefficients[2],
                       e$coefficients[2]/x$coefficients[2],f$coefficients[2]/x$coefficients[2],
                       g$coefficients[2]/x$coefficients[2],h$coefficients[2]/x$coefficients[2]),
           estimate_2sls = c(a2$coefficients[2],b2$coefficients[2],c2$coefficients[2],d2$coefficients[2],
                            e2$coefficients[2],f2$coefficients[2],g2$coefficients[2],h2$coefficients[2]),
           lb_2sls = c(confint(a2)[2,1],confint(b2)[2,1],confint(c2)[2,1],confint(d2)[2,1],confint(e2)[2,1],
                     confint(f2)[2,1],confint(g2)[2,1],confint(h2)[2,1]),
           ub_2sls = c(confint(a2)[2,2],confint(b2)[2,2],confint(c2)[2,2],confint(d2)[2,2],confint(e2)[2,2],
                     confint(f2)[2,2],confint(g2)[2,2],confint(h2)[2,2]))
```

A data.frame: 8 × 5

| test | estimate\_ratio | estimate\_2sls | lb\_2sls | ub\_2sls |
| --- | --- | --- | --- | --- |
| <chr> | <dbl> | <dbl> | <dbl> | <dbl> |
| MAT | 3.2637558 | 3.3808299 | 2.11306203 | 4.6485978 |
| REYI | 0.8237618 | 0.8704631 | 0.53211159 | 1.2088146 |
| REYII | 0.6947586 | 0.7401899 | 0.39413753 | 1.0862424 |
| FAS | 2.0930431 | 2.0297506 | 1.30171509 | 2.7577861 |
| PMT | 0.6844675 | 0.6733431 | 0.47064204 | 0.8760442 |
| TMT | 0.1259747 | 0.1225483 | -0.01087867 | 0.2559752 |
| VERBAL1 | 2.8801023 | 3.0471075 | 2.09107785 | 4.0031372 |
| VERBAL2 | 3.2408370 | 3.4323217 | 2.34427767 | 4.5203657 |

## 2SLS without BMI¶

In [54]:

```
a2<- ivreg(F1MAT ~ sex + age_b + HGT_HEIGHT_M_COM|nonferm_dairy|lp+sex+age_b+HGT_HEIGHT_M_COM,data = ch)
b2<- ivreg(F1REY1 ~ sex + age_b +HGT_HEIGHT_M_COM|nonferm_dairy|lp+sex+age_b+HGT_HEIGHT_M_COM,data = ch)
c2<- ivreg(F1REY2 ~ sex + age_b +HGT_HEIGHT_M_COM|nonferm_dairy|lp+sex+age_b+HGT_HEIGHT_M_COM,data = ch)
d2<- ivreg(F1FAS ~ sex + age_b + HGT_HEIGHT_M_COM|nonferm_dairy|lp+sex+age_b+HGT_HEIGHT_M_COM,data = ch)
e2<- ivreg(F1PMT ~ sex + age_b + HGT_HEIGHT_M_COM|nonferm_dairy|lp+sex+age_b+HGT_HEIGHT_M_COM,data = ch)
f2<- ivreg(F1TMT ~ sex + age_b + HGT_HEIGHT_M_COM|nonferm_dairy|lp+sex+age_b+HGT_HEIGHT_M_COM,data = ch)
g2<- ivreg(F1VERBAL1 ~sex+age_b+ HGT_HEIGHT_M_COM|nonferm_dairy|lp+sex+age_b+HGT_HEIGHT_M_COM,data = ch)
h2<- ivreg(F1VERBAL2 ~sex+age_b+ HGT_HEIGHT_M_COM|nonferm_dairy|lp+sex+age_b+HGT_HEIGHT_M_COM,data = ch)

data.frame(test = c("MAT", "REYI", "REYII", "FAS", "PMT", "TMT","VERBAL1", "VERBAL2"),
           estimate_ratio = c(a$coefficients[2]/o$coefficients[2],b$coefficients[2]/o$coefficients[2],
                       c$coefficients[2]/o$coefficients[2],d$coefficients[2]/o$coefficients[2],
                       e$coefficients[2]/o$coefficients[2],f$coefficients[2]/o$coefficients[2],
                       g$coefficients[2]/o$coefficients[2],h$coefficients[2]/o$coefficients[2]),
          estimate_2sls = c(a2$coefficients[2],b2$coefficients[2],c2$coefficients[2],d2$coefficients[2],
                            e2$coefficients[2],f2$coefficients[2],g2$coefficients[2],h2$coefficients[2]),
          lb_2sls = c(confint(a2)[2,1],confint(b2)[2,1],confint(c2)[2,1],confint(d2)[2,1],confint(e2)[2,1],
                     confint(f2)[2,1],confint(g2)[2,1],confint(h2)[2,1]),
          ub_2sls = c(confint(a2)[2,2],confint(b2)[2,2],confint(c2)[2,2],confint(d2)[2,2],confint(e2)[2,2],
                     confint(f2)[2,2],confint(g2)[2,2],confint(h2)[2,2]))
```

A data.frame: 8 × 5

| test | estimate\_ratio | estimate\_2sls | lb\_2sls | ub\_2sls |
| --- | --- | --- | --- | --- |
| <chr> | <dbl> | <dbl> | <dbl> | <dbl> |
| MAT | -14.1843230 | 3.3551394 | 2.14375420 | 4.5665247 |
| REYI | -3.5800791 | 0.8440954 | 0.52391765 | 1.1642731 |
| REYII | -3.0194297 | 0.7027622 | 0.37117127 | 1.0343532 |
| FAS | -9.0963914 | 2.0801331 | 1.35117287 | 2.8090934 |
| PMT | -2.9747044 | 0.7027375 | 0.50890807 | 0.8965669 |
| TMT | -0.5474878 | 0.1207010 | -0.01607791 | 0.2574799 |
| VERBAL1 | -12.5169605 | 3.0222891 | 2.15622401 | 3.8883541 |
| VERBAL2 | -14.0847178 | 3.4024319 | 2.41461384 | 4.3902501 |

### Total dairy¶

In [55]:

```
a2<- ivreg(F1MAT ~ sex + age_b + HGT_HEIGHT_M_COM|total_dairy|lp+sex+age_b+HGT_HEIGHT_M_COM,data = ch)
b2<- ivreg(F1REY1 ~ sex + age_b +HGT_HEIGHT_M_COM|total_dairy|lp+sex+age_b+HGT_HEIGHT_M_COM,data = ch)
c2<- ivreg(F1REY2 ~ sex + age_b +HGT_HEIGHT_M_COM|total_dairy|lp+sex+age_b+HGT_HEIGHT_M_COM,data = ch)
d2<- ivreg(F1FAS ~ sex + age_b + HGT_HEIGHT_M_COM|total_dairy|lp+sex+age_b+HGT_HEIGHT_M_COM,data = ch)
e2<- ivreg(F1PMT ~ sex + age_b + HGT_HEIGHT_M_COM|total_dairy|lp+sex+age_b+HGT_HEIGHT_M_COM,data = ch)
f2<- ivreg(F1TMT ~ sex + age_b + HGT_HEIGHT_M_COM|total_dairy|lp+sex+age_b+HGT_HEIGHT_M_COM,data = ch)
g2<- ivreg(F1VERBAL1 ~sex+age_b+ HGT_HEIGHT_M_COM|total_dairy|lp+sex+age_b+HGT_HEIGHT_M_COM,data = ch)
h2<- ivreg(F1VERBAL2 ~sex+age_b+ HGT_HEIGHT_M_COM|total_dairy|lp+sex+age_b+HGT_HEIGHT_M_COM,data = ch)

data.frame(test = c("MAT", "REYI", "REYII", "FAS", "PMT", "TMT","VERBAL1", "VERBAL2"),
           estimate_ratio = c(a$coefficients[2]/o$coefficients[2],b$coefficients[2]/o$coefficients[2],
                       c$coefficients[2]/o$coefficients[2],d$coefficients[2]/o$coefficients[2],
                       e$coefficients[2]/o$coefficients[2],f$coefficients[2]/o$coefficients[2],
                       g$coefficients[2]/o$coefficients[2],h$coefficients[2]/o$coefficients[2]),
          estimate_2sls = c(a2$coefficients[2],b2$coefficients[2],c2$coefficients[2],d2$coefficients[2],
                            e2$coefficients[2],f2$coefficients[2],g2$coefficients[2],h2$coefficients[2]),
          lb_2sls = c(confint(a2)[2,1],confint(b2)[2,1],confint(c2)[2,1],confint(d2)[2,1],confint(e2)[2,1],
                     confint(f2)[2,1],confint(g2)[2,1],confint(h2)[2,1]),
          ub_2sls = c(confint(a2)[2,2],confint(b2)[2,2],confint(c2)[2,2],confint(d2)[2,2],confint(e2)[2,2],
                     confint(f2)[2,2],confint(g2)[2,2],confint(h2)[2,2]))
```

A data.frame: 8 × 5

| test | estimate\_ratio | estimate\_2sls | lb\_2sls | ub\_2sls |
| --- | --- | --- | --- | --- |
| <chr> | <dbl> | <dbl> | <dbl> | <dbl> |
| MAT | -14.1843230 | 3.2736023 | 2.02411519 | 4.5230894 |
| REYI | -3.5800791 | 0.8309823 | 0.49882556 | 1.1631391 |
| REYII | -3.0194297 | 0.6949802 | 0.35508552 | 1.0348750 |
| FAS | -9.0963914 | 1.9611413 | 1.24127499 | 2.6810077 |
| PMT | -2.9747044 | 0.6757905 | 0.47371381 | 0.8778671 |
| TMT | -0.5474878 | 0.1163964 | -0.01628278 | 0.2490756 |
| VERBAL1 | -12.5169605 | 3.0167817 | 2.06923636 | 3.9643271 |
| VERBAL2 | -14.0847178 | 3.3964558 | 2.31818408 | 4.4747275 |

## 2SLS no butter with BMI¶

In [56]:

```
a2<- ivreg(F1MAT ~ sex + age_b + HGT_HEIGHT_M_COM+bmi_b|nobutternonferm_dairy|lp+sex+age_b+HGT_HEIGHT_M_COM+bmi_b,data = ch)
b2<- ivreg(F1REY1 ~ sex + age_b +HGT_HEIGHT_M_COM+bmi_b|nobutternonferm_dairy|lp+sex+age_b+HGT_HEIGHT_M_COM+bmi_b,data = ch)
c2<- ivreg(F1REY2 ~ sex + age_b +HGT_HEIGHT_M_COM+bmi_b|nobutternonferm_dairy|lp+sex+age_b+HGT_HEIGHT_M_COM+bmi_b,data = ch)
d2<- ivreg(F1FAS ~ sex + age_b + HGT_HEIGHT_M_COM+bmi_b|nobutternonferm_dairy|lp+sex+age_b+HGT_HEIGHT_M_COM+bmi_b,data = ch)
e2<- ivreg(F1PMT ~ sex + age_b + HGT_HEIGHT_M_COM+bmi_b|nobutternonferm_dairy|lp+sex+age_b+HGT_HEIGHT_M_COM+bmi_b,data = ch)
f2<- ivreg(F1TMT ~ sex + age_b + HGT_HEIGHT_M_COM+bmi_b|nobutternonferm_dairy|lp+sex+age_b+HGT_HEIGHT_M_COM+bmi_b,data = ch)
g2<- ivreg(F1VERBAL1 ~sex+age_b+ HGT_HEIGHT_M_COM+bmi_b|nobutternonferm_dairy|lp+sex+age_b+HGT_HEIGHT_M_COM+bmi_b,data = ch)
h2<- ivreg(F1VERBAL2 ~sex+age_b+ HGT_HEIGHT_M_COM+bmi_b|nobutternonferm_dairy|lp+sex+age_b+HGT_HEIGHT_M_COM+bmi_b,data = ch)

data.frame(test = c("MAT", "REYI", "REYII", "FAS", "PMT", "TMT","VERBAL1", "VERBAL2"),
           estimate_ratio = c(a$coefficients[2]/o$coefficients[2],b$coefficients[2]/o$coefficients[2],
                       c$coefficients[2]/o$coefficients[2],d$coefficients[2]/o$coefficients[2],
                       e$coefficients[2]/o$coefficients[2],f$coefficients[2]/o$coefficients[2],
                       g$coefficients[2]/o$coefficients[2],h$coefficients[2]/o$coefficients[2]),
          estimate_2sls = c(a2$coefficients[2],b2$coefficients[2],c2$coefficients[2],d2$coefficients[2],
                            e2$coefficients[2],f2$coefficients[2],g2$coefficients[2],h2$coefficients[2]),
          lb_2sls = c(confint(a2)[2,1],confint(b2)[2,1],confint(c2)[2,1],confint(d2)[2,1],confint(e2)[2,1],
                     confint(f2)[2,1],confint(g2)[2,1],confint(h2)[2,1]),
          ub_2sls = c(confint(a2)[2,2],confint(b2)[2,2],confint(c2)[2,2],confint(d2)[2,2],confint(e2)[2,2],
                     confint(f2)[2,2],confint(g2)[2,2],confint(h2)[2,2]))
```

A data.frame: 8 × 5

| test | estimate\_ratio | estimate\_2sls | lb\_2sls | ub\_2sls |
| --- | --- | --- | --- | --- |
| <chr> | <dbl> | <dbl> | <dbl> | <dbl> |
| MAT | -14.1843230 | 4.5950920 | 2.95635620 | 6.2338278 |
| REYI | -3.5800791 | 1.1839610 | 0.74423678 | 1.6236853 |
| REYII | -3.0194297 | 0.9968775 | 0.54514323 | 1.4486118 |
| FAS | -9.0963914 | 2.8379701 | 1.86323803 | 3.8127021 |
| PMT | -2.9747044 | 0.9251957 | 0.66533723 | 1.1850541 |
| TMT | -0.5474878 | 0.1665426 | -0.01348864 | 0.3465739 |
| VERBAL1 | -12.5169605 | 4.1111191 | 2.91016336 | 5.3120748 |
| VERBAL2 | -14.0847178 | 4.6312619 | 3.26208579 | 6.0004380 |
